# Calcium Impregnated Silica Gel in the Domino Reaction Involving Irreversible Aldol Addition, Dehydration, and Michael Addition

**DOI:** 10.1021/acs.joc.4c02340

**Published:** 2025-04-16

**Authors:** Jih Ru Hwu, Khagendra Prasad Bohara, Animesh Roy, Wen-Chieh Huang, Kuo-Chu Hwang, Chun-Cheng Lin, Kao Shu Chuang, Shu-Yu Lin, Shwu-Chen Tsay

**Affiliations:** † Department of Chemistry and Frontier Research Center on Fundamental and Applied Sciences of Matters, National Tsing Hua University, Hsinchu 30013, Taiwan; ‡ Institute of Biotechnology and Pharmaceutical Research, National Health Research Institutes, Miaoli County 350401, Taiwan; § Department of Green Material Technology, Green Technology Research Institute, CPC Corporation, Kaohsiung City 81126, Taiwan

## Abstract

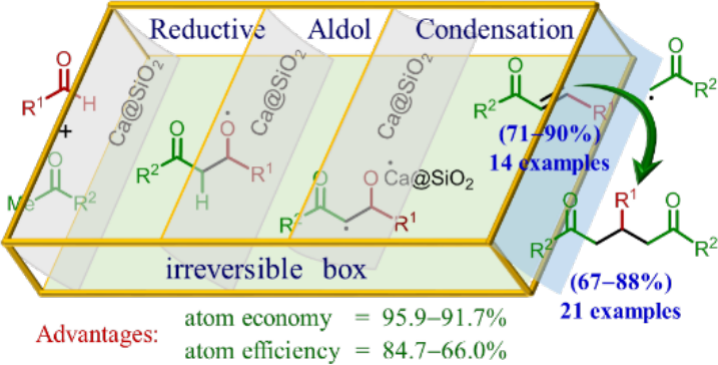

An innovative method was developed for the performance of aldol additions in an irreversible fashion by the use of calcium metal impregnated silica gel (Ca@SiO_2_) as a remarkable reducing reagent. In this approach, Ca@SiO_2_ drove the reaction forward, prevented reversibility, and ensured the formation of the desired products. Thus, in the presence of Ca@SiO_2_ (3.0 equiv), aldehydes (1.0 equiv) condensed with ketones (1.0 equiv) in 2-MeTHF to yield α,β-unsaturated enones in 71–90% yields at 25 °C. Additionally, a domino reaction involving successive aldol addition, dehydration, and Michael addition was developed for the preparation of 1,5-diketones. Accordingly, when aldehydes (1.0 equiv) were allowed to react with ketones (2.2 equiv) and Ca@SiO_2_(4.0 equiv), 1,5-diketones were produced in 67–88% yields. These reactions involved radical processes, where Ca@SiO_2_ abstracted two α hydrogen atoms from ketones and the oxygen atom from aldehydes to form CaH_2_@SiO_2_ and CaO@SiO_2_, respectively. These species were confirmed by powder X-ray diffraction analysis. The resultant impregnated silica gel species were solid and insoluble in the reaction mixtures, which made the addition reactions irreversible. This method represents a significant advancement in aldol condensation reactions and offers the advantages of both atom economy and atom efficiency.

## Introduction

The aldol condensation^1−3^ of two carbonyl compounds is considered one of the most popular reactions for the formation of carbon–carbon bonds, which plays a crucial role in synthetic chemistry. Nevertheless, traditional aldol condensation has several limitations that restrict its broader applications. First, aldol condensations involving the use of acid or base catalysts are often reversible as the β-hydroxy carbonyl intermediates may revert to the starting aldehydes or ketones.^4^ Without these catalysts, aldol intermediates tend to be more stable. Second, during cross-coupling between different carbonyl compounds, undesirable self-condensation may occur.^4^ Third, competing polycondensation could generate unwanted polyols as byproducts.^4^ Fourth, the desired α,β-unsaturated enone products may undergo Michael reactions with enolate anions, leading to complex mixtures.^4,5^

Calcium metal is known to react vigorously with water to liberate hydrogen gas and form calcium hydroxide at room temperature.^11^ Finely divided calcium spontaneously ignites in air.^12^ Moreover, it can reduce aldehydes and ketones to alcohols.^12−14^

We planned to develop a conceptually different and irreversible process using a new calcium-based reagent. This method would obviate the use of acids or bases as catalysts as well as water generation.^6−10^ Thus, it could facilitate the production of α,β-unsaturated enones in good yields. This method could also convert mixtures of aldehydes and ketones to 1,5-diketones, which are valuable building blocks^15^ for the synthesis of fused rings found in alkaloids, steroids, terpenes, and more.^16^ Some 1,5-diketones exhibit various biological activities, including antidiabetic, anti-infective, anti-inflammatory, and antitumor properties.^17^

**Scheme 1:**
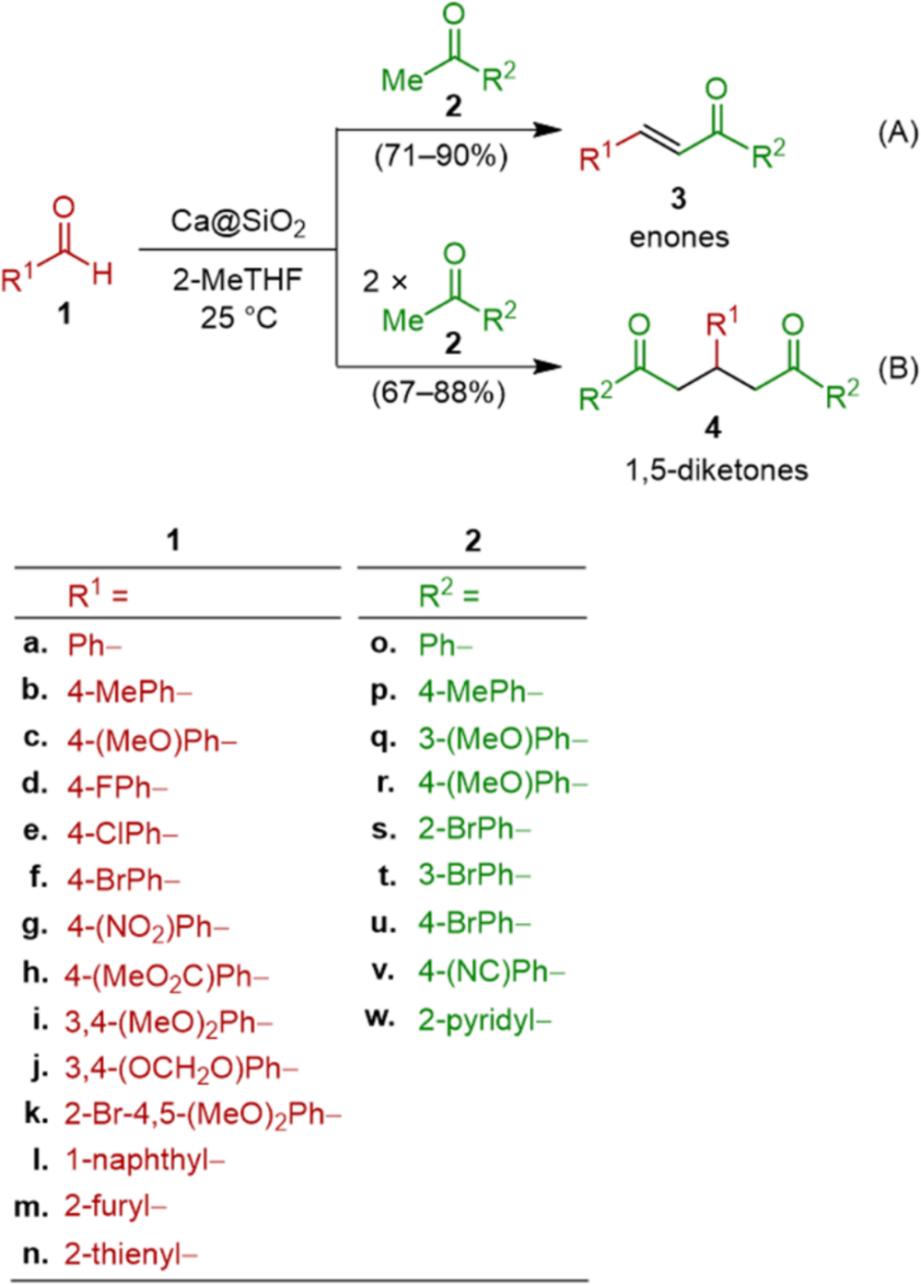
Formation of Enones **3** and 1,5-Diketones **4** through Pathway A and Pathway B, Respectively, with Ca@SiO_2_

Herein, we report our findings on impregnating silica gel with calcium metal to form fine Ca@SiO_2_ powders, which mitigate the violent reactivity of the calcium metal. It is able to control the condensation of aldehydes with ketones. Consequently, enones **3** and 1,5-diketones **4** can be generated through a different type of aldol condensation between aldehydes **1** and ketones **2**, as depicted in Scheme 1A,B. These reactions proceed through a domino process involving reductive radical intermediates and are distinct from all previously reported aldol reactions.

## Results

### Ca@SiO_2_ in Aldol Condensation

We introduced Ca@SiO_2_ gray powders (containing 40.0 wt % calcium metal) into a solution containing benzaldehyde (**1a**, 1.0 equiv) and acetophenone (**2o**, 1.0 equiv) at 25 °C (Scheme 1A). Different solvents, including tetrahydrofuran (THF), 2-methyltetrahydrofuran (2-MeTHF), and acetonitrile (CH_3_CN), were utilized separately as the reaction media (see Table 1 herein and Table S1 in the Supporting Information). After the reaction mixture was stirred under dry nitrogen for 18 h, the inorganic residue in the heterogeneous solution was filtered. The filtrate was subsequently concentrated and purified by silica gel column chromatography. Consequently, the pure α,β-unsaturated enone **3ao** was isolated, and the yields were found to be correlated with the equivalents of Ca@SiO_2_ utilized. Optimal results were achieved by the use of Ca@SiO_2_ (3.0 equiv) in 2-MeTHF (entry 6 in Table 1). An increment of the Ca@SiO_2_ amount did not result in higher yields for the desired product **3ao**. At 40.0 wt % in Ca@SiO_2_, the Ca was optimally adsorbed or encapsulated within the silica matrix, which resulted in optimal performance as shown in entry 6. However, when the Ca content was increased to 50.0 wt % (entry 7), the reducing power dropped, likely due to insufficient protection of the excess Ca by the silica gel. Consequently, the reducing efficiency of Ca@SiO_2_ decreased. Additionally, anhydrous conditions were imperative for the success of this reaction.

**Table 1:** Optimization of Reaction Conditions^a^ for the Reactions **1a** + **2o** → **3ao** and **1a** + **2** × **2o** → **4ao** in the Presence of Ca@SiO_2_

			Ca in Ca@SiO_2_		
entry	product	solvent	equiv	wt %	yield^b^ (%)
1	**3ao**	THF	0	40.0	0
2	**3ao**	THF	2.0	40.0	48
3	**3ao**	THF	3.0	40.0	83
4	**3ao**	THF	3.5	40.0	83
5	**3ao**	2-MeTHF	3.0	30.0	65
6	**3ao**	2-MeTHF	3.0	40.0	85
7	**3ao**	2-MeTHF	3.0	50.0	60
8	**3ao**	CH_3_CN	3.0	40.0	82
9	**4ao**	THF	0	40.0	0
10	**4ao**	THF	3.0	40.0	62
11	**4ao**	THF	4.0	40.0	81
12	**4ao**	THF	4.5	40.0	81
13	**4ao**	2-MeTHF	4.0	30.0	61
14	**4ao**	2-MeTHF	4.0	40.0	82
15	**4ao**	2-MeTHF	4.0	50.0	58
16	**4ao**	CH_3_CN	4.0	40.0	80

a Conditions for the reactions: **1a** (1.0 equiv), **2o** (1.0 equiv for **3ao** or 2.2 equiv for **4ao**), Ca@SiO_2_ containing 30.0–50.0 wt % of Ca metal, reaction time 18 h for **3ao** and 20 h for **4ao** at 25 °C.

b Isolated yield.

In radical chemistry, 2,2,6,6-tetramethyl-1-piperidinyl-N-oxide (TEMPO)^18^ is commonly used as a radical trap. It reacts rapidly with radical species at rates ranging from approximately 5 × 10^7^ to 2 × 10^9^ M^–1^s^–1^. In the reaction of **1a** + **2o** + Ca@SiO_2_ in 2-MeTHF (entry 6, Table 1), the addition of TEMPO hindered the formation of enone **3ao**. Separation of the resultant mixtures to obtain the pure products was challenging, as multiple radical species could form and become trapped in a domino process.

The scope of this innovative condensation reaction illustrated in Scheme 1A was explored with regard to the starting materials of **1** and **2** bearing various functional groups. These included −Br, −OMe, −OCH_2_O–, −C=O, −CO_2_R, phenyl, furyl, thienyl, and pyridyl groups. The use of Ca@SiO_2_ (3.0 equiv) enabled the synthesis of 14 different α,β-unsaturated enones **3** (71–90% yields) as shown in Table 2. These functional groups withstood the reducing activity of Ca@SiO_2_ and remained intact during the formation of the products **3**.

**Table 2:**
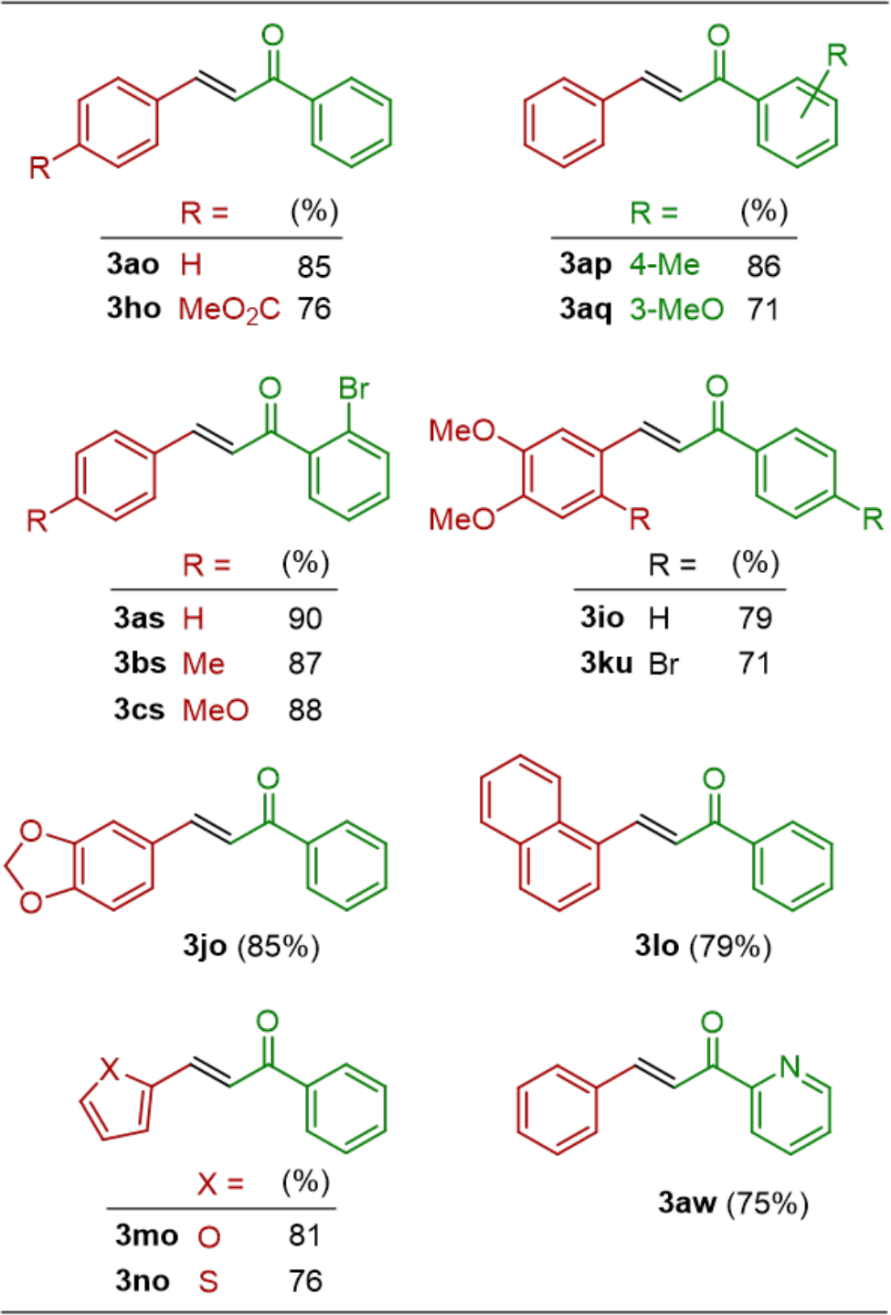
Structures and Yields of Enones **3** Generated from the Reaction^a^ (A) Shown in Scheme 1

a Conditions for the reaction: **1** (1.0 equiv) + **2** (1.0 equiv) + Ca@SiO_2_ (3.0 equiv) → **3** at 25 °C for 18 h, and isolated yield.

### Ca@SiO_2_ for the Formation of 1,5-Diketones

With an excess of ketones, the above aldol reaction may potentially be followed by a sequential Michael addition.^4^ To investigate this feasibility by using Ca@SiO_2_, we treated a solution containing benzaldehyde (**1a**, 1.0 equiv) and ketone **2o** (2.2 equiv) with various equivalents of Ca@SiO_2_ at 25 °C for 20 h. Additionally, the reactions were conducted in different solvents, as listed in Tables 1 and S2 in the Supporting Information. The results revealed that the desired 1,5-diketone **4ao** was produced in its highest yield (82%) when Ca@SiO_2_ (4.0 equiv) was used in 2-MeTHF (entry 14 in Table 1). Consequently, 21 examples of 1,5-diketones **4** were obtained in 67–88% yields (Table 3) under these optimal conditions. The structures of all enones **3** and 1,5-diketones **4** were identified on the basis of their spectroscopic characteristics, as described in the Supporting Information.

**Table 3:**
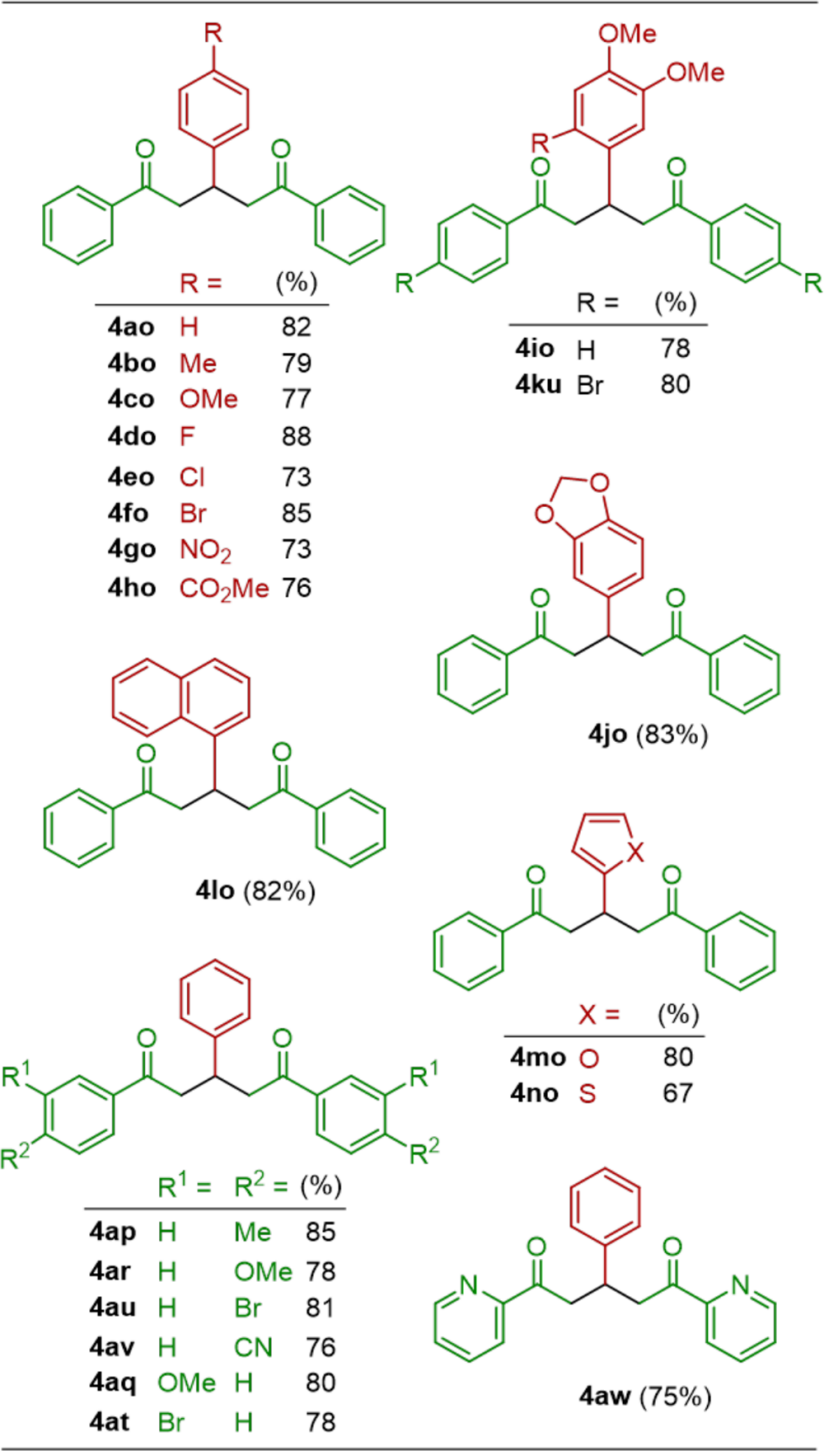
Structures and Yields of 1,5-Diketones **4** Generated from the Reaction^a^ (B) Shown in Scheme 1

a Conditions for the reaction: **1** (1.0 equiv) + **2** (2.2 equiv) + Ca@SiO_2_ (4.0 equiv) → **4** at 25 °C for 20 h, and isolated yield.

### Preparation of Ca@SiO_2_ and Evidence on the Formation of Ca@SiO_2_, CaH_2_@SiO_2_, and CaO@SiO_2_

**Figure 1:**
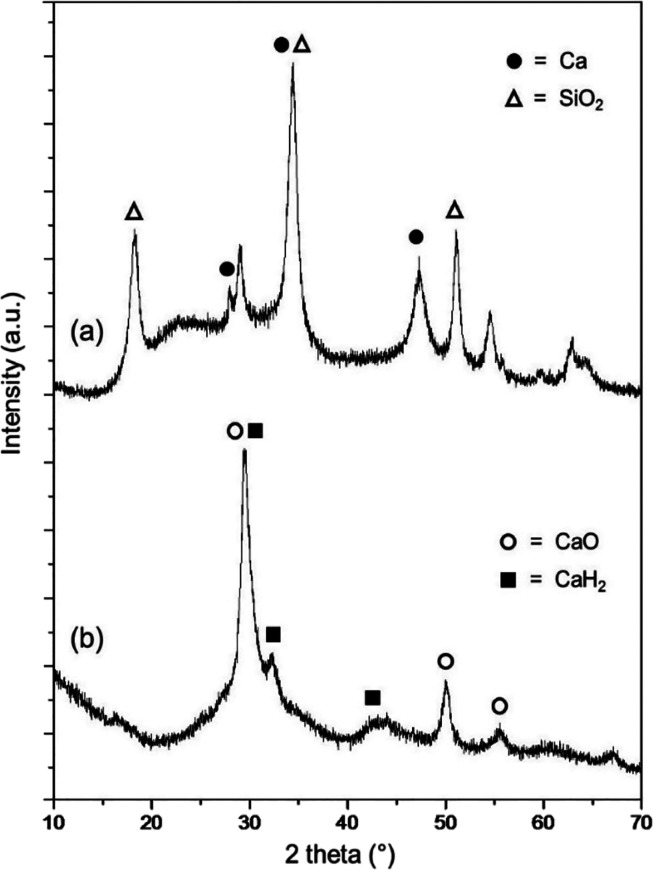
XRD patterns of (a) the Ca@SiO_2_ powders before their application to the aldol condensation and (b) the solid residues after the aldol addition followed by dehydration and Michael addition.

We developed a method to prepare the reagent Ca@SiO_2_ by impregnating silica gel powders (particle size 40–63 μm, 230–400 mesh) with dissolved Ca granules in liquid ammonia at –78 °C. The entire procedure must be carefully conducted under anhydrous conditions and in an argon atmosphere in a hood. The preparation of this calcium-based reagent and the necessary precautions are fully described in the Supporting Information. Its chemical composition was determined by X-ray powder diffraction (XRD) on the basis of the characteristic diffuse scattering peaks.^19^ Our sample exhibited a set of three diffraction peaks at 2θ of 27.8, 33.4, and 47.2° in Figure 1a, corresponding to Ca metal (indicated by •). These data are consistent with those reported in the Joint Committee on Powder Diffraction Standards (JCPDS) file No. 23-0430.^20a,21a^ Additionally, another set of three diffraction peaks in the same Figure 1a at 2θ of 18.2, 34.2, and 51.0° (indicated by Δ) were indexed to the SiO_2_ component in JCPDS file No. 46-1045.^20b,21b^ A slight shift in the diffraction angle associated with these peaks resulted from minor lattice distortion during impregnation.^22^

After the completion of the sequential aldol addition/dehydration/Michael addition as depicted in Scheme 1B, the solid residues in the reaction mixture were also analyzed by means of XRD. It exhibited two sets of peaks, as shown in Figure 1b. One set, with three peaks at 2θ of 30.8, 32.3, and 42.1° (indicated by ^■^), aligned well with those of CaH_2_ reported in JCPDS file No. 65-2384.^20c,21c^ The other set displayed three peaks at 2θ of 29.4, 50.1, and 55.5° (indicated by ○), which corresponded closely with those of CaO reported in JCPDS file No. 17-0912.^20d,21d^ These results clearly indicate the formation of CaH_2_@SiO_2_ and CaO@SiO_2_ in the aldol addition/dehydration/Michael addition by the use of Ca@SiO_2_ as the reducing agent. The absence of SiO_2_ peaks in Figure 1b suggests that the SiO_2_ in the solid residues was in amorphous forms or possessed structures with very short-range crystalline order.^23^

## Discussion

### Mechanism and Roles of the Reducing Agent Ca@SiO_2_ in the Aldol Addition and the Sequential Dehydration/Michael Addition

Conventional methods for aldol condensation and Michael reactions typically do not involve the use of a reducing agent. Nevertheless, our findings indicate that the utilization of the reducing agent Ca@SiO_2_ enabled the direct removal of hydrogen atoms and an oxygen atom from the ketones and aldehydes, respectively, during the aldol condensation. According to Le Chatelier’s principle,^24^ the formation of insoluble solids CaH_2_@SiO_2_ and CaO@SiO_2_ (instead of H_2_O) in the reaction mixture prevents the aldol condensation from being reversible. Therefore, the generation of enones **3** and 1,5-diketones **4** in good-to-high yields is primarily due to the role played by the reducing agent Ca@SiO_2_.

The condensation of benzaldehydes **1** with ketones **2** to afford either enones **3** or 1,5-diketones **4** depended upon the applied amounts of **2** and Ca@SiO_2_. Use of ketones **2** (1.0 equiv) along with Ca@SiO_2_ (3.0 equiv) afforded enones **3** as the exclusive products. Use of ketones **2** (2.2 equiv) and Ca@SiO_2_ (4.0 equiv) to react with benzaldehydes **1** gave 1,5-diketones **4** as the final products through a domino process involving aldol addition, dehydration, and a Michael 1,4-addition reaction.

**Scheme 2:**
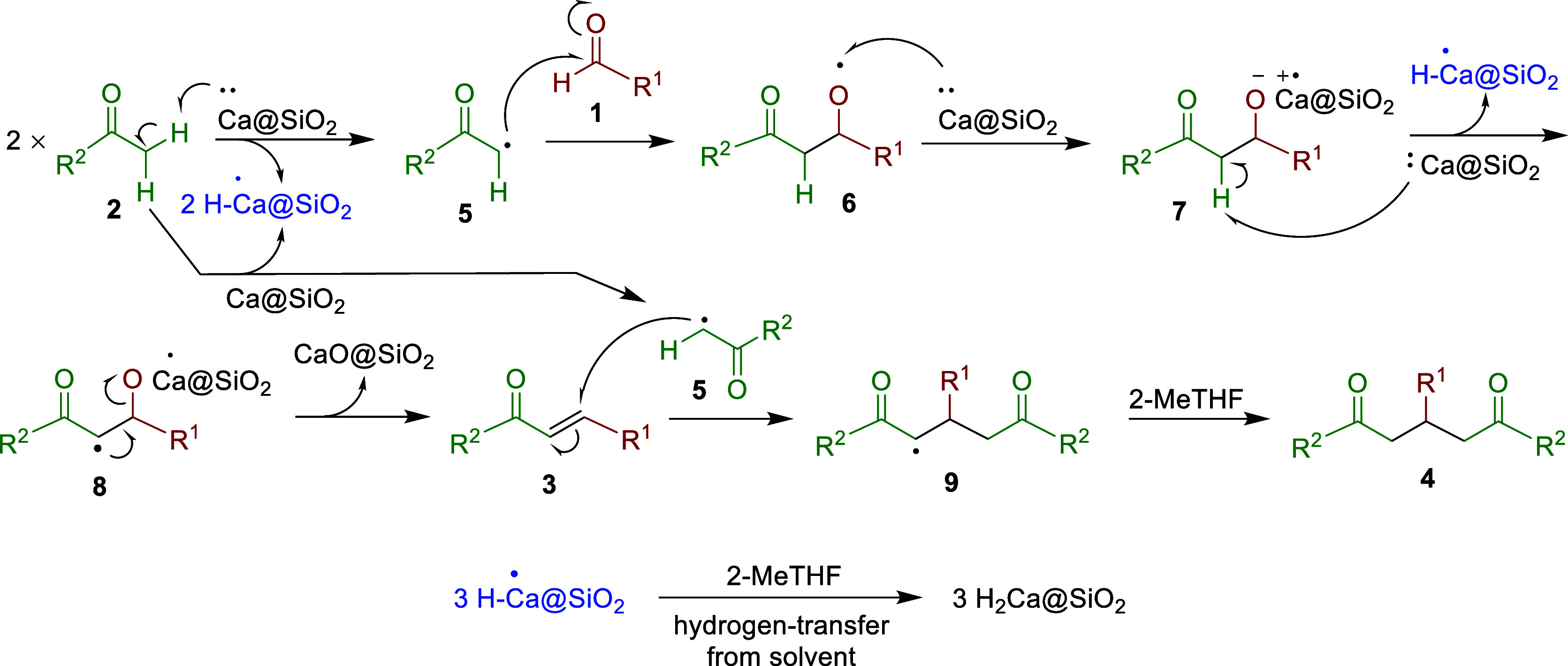
Plausible Mechanism for the Formation of α,β-Unsaturated Enones **3** and 1,5-diketones **4** from Aldehydes **1** and Ketones **2** in the Presence of Ca@SiO_2_

The mechanism depicted in Scheme 2 elucidates our experimental findings. The initial equivalent of noncohesive Ca@SiO_2_ powders abstracts a hydrogen atom from ketones **2**(25) to generate the α-ketonic carboradicals^26^**5** and H–^•^Ca@SiO_2_. Subsequently, these carboradicals **5** add to aldehydes **1**, leading to the formation of aldol radicals **6**.^27^ The second equivalent of Ca@SiO_2_ donates one electron to alkoxy radicals **6**,^28^ resulting in the generation of calcium alkoxides **7**. Then, the third equivalent of Ca@SiO_2_ abstracts an α-hydrogen atom^25^ from intermediates **7** to form the carboradicals **8**, which undergo elimination^29^ to yield CaO@SiO_2_ and the aldol condensation products **3**.

During the process of **2** + 3 × Ca@SiO_2_ + **1** → → → → → **3** + 2 × H–^•^Ca@SiO_2_ + CaO@SiO_2_, the radical intermediate H–^•^Ca@SiO_2_ can further trap an α-hydrogen atom from the solvent 2-MeTHF.^30,31^ Consequently, CaH_2_@SiO_2_ is generated in the reaction residue, as confirmed by our XRD analysis shown in Figure 1b.

When two equivalents of acetophenones **2** are used, two equivalents of the α-ketonic carboradicals **5** could be generated. The second equivalent of **5**, generated from the hydrogen abstraction by the fourth equivalent of Ca@SiO_2_, would add to the enones **3**(32) to form diketonic radicals **9**.^33^ Trap a hydrogen atom from the solvent 2-MeTHF^31^ by the radicals **9** yields the Michael addition products **4**. The entire process of **1** + **2** → **3** → **4** involves various radical intermediates, which are generated by Ca@SiO_2_. The products **3** and **4**, bearing more than ten different reducible functional groups, as shown in Scheme 1A,B, were isolated in good-to-high yields. These results indicate that both the reducing agent Ca@SiO_2_ and the reaction conditions were mild.

Our developed procedure included straightforward manipulation, mild conditions, and easy purification. Notably, it did not necessitate the presence of acids or bases as catalysts. Consequently, an aqueous workup of the reaction mixture was unnecessary. Additionally, common issues in organic synthesis, such as possible self-condensations of carbonyl compounds and undesirable polycondensation, were not observed as side reactions when Ca@SiO_2_ was employed. Moreover, the ability to control the stoichiometric ratio of the reagent Ca@SiO_2_ and ketones facilitated the generation of the desired α,β-unsaturated enones or Michael adducts separately in impressive yields.

### Atom Economy and Atom Efficiency

To assess the green chemistry characteristics of our developed processes shown in Scheme 1, we calculated their atom economy^34^ and atom efficiency.^35^ The detailed results are provided in Tables S3 and S4 of the Supporting Information. For enone formation, as shown in Scheme 1A, the highest atom economy was 95.9% for **3ku**, whereas the lowest was 91.7% for **3mo**. In terms of atom efficiency, the highest was 84.7% for **3as**, and the lowest was 66.0% for **3aq**. For 1,5-diketone formation, as shown in Scheme 1B, the highest atom economy was 97.2% for **4ku**, whereas the lowest was 94.6% for **4mo**. In terms of atom efficiency, the highest efficiency was 83.7% for **4do**, while the lowest was 59.8% for **4no**. These impressive results associated with the two irreversible aldol condensation reactions meet the principles of green chemistry.^36,37^ Nevertheless, the preparation of Ca@SiO_2_ was conducted in liquid ammonia at –78 °C, an energy-intensive process that required a substantial amount of liquid ammonia. Additionally, the Ca@SiO_2_ reagent was consumed in excess during a single-batch reaction. These unfavorable characteristics need to be improved to make the new reagent suitable for “green” applications in the future.

## Conclusions

A conceptually progressive approach was developed to perform the aldol condensation by use of the innovative reducing reagent Ca@SiO_2_, which contains 40.0 wt % of calcium. This calcium-based reagent was designed to prevent the formation of H_2_O as a byproduct. Upon reaction with aldehydes and ketones in the aldol condensation, Ca@SiO_2_ underwent oxidation to form stable CaH_2_@SiO_2_ and CaO@SiO_2_ as insoluble powders. Consequently, the reversible process became unattainable, leading to the effective production of the desired products: α,β-unsaturated enones and 1,5-diketones. To the best of our knowledge, *these findings represent pioneering examples of reductive aldol condensation* and the Michael reaction. These outcomes were achieved through a domino reaction that facilitates the formation of C–C single and double bonds.

## Data Availability

The data underlying this study are available in the published article and its Supporting Information.

## Supporting Information Available

Experimental details, spectroscopic data of all new compounds, references, and spectra (PDF)

## References

[ref1] Carey F. A.; Sundberg R. J. Advanced Organic Chemistry Part B: Reactions and Synthesis, 6th ed.; Plenum Publishers: New York, 2001.

[ref2] Nielsen A. T.; Houlihan W. J. The aldol condensation. Org. React. 2011, 16, 1–438. 10.1002/0471264180.or016.01.

[ref3] Perrin C. L.; Chang K.-L. The complete mechanism of an aldol condensation. J. Org. Chem. 2016, 81, 5631–5635. 10.1021/acs.joc.6b00959. 27281298

[ref4] Mukaiyama T. The directed aldol reaction. Org. React. 1982, 28, 203–331. 10.1002/0471264180.or028.03.

[ref5] Saito S.; Yamamoto H. Directed aldol condensation. Chem.—Eur. J. 1999, 5, 1959–1962. 10.1002/(SICI)1521-3765(19990702)5:7<1959::AID-CHEM1959>3.3.CO;2-Z.

[ref11] Vrana L. M. Calcium and Calcium Alloys. Kirk-Othmer Encyclopedia of Chemical Technology; John Wiley & Sons, Inc., 2011; pp. 1–10.

[ref12] Hluchan S. E.; Pomerantz K. Calcium and Calcium Alloys. In Ullmann’s Encyclopedia of Industrial Chemistry; Wiley-VCH Verlag KGaA: Weinheim, 2006; pp. 483–494.

[ref13] Hwu J. R.; King K.-Y. Calcium in Organic Synthesis. In Main Group Metals in Organic Synthesis; Yamamoto H.; Oshima K., Eds.; Wiley-VCH Verlag KGaA: Weinheim, 2004; pp. 155–174.

[ref14] Hwu J. R.; Wein Y. S.; Leu Y.-J. Calcium metal in liquid ammonia for selective reduction of organic compounds. J. Org. Chem. 1996, 61, 1493–1499. 10.1021/jo951219c.

[ref6] Ito Y.; Hirao T.; Saegusa T. Synthesis of α,β-unsaturated carbonyl compounds by palladium(II)-catalyzed dehydrosilylation of silyl enol ethers. J. Org. Chem. 1978, 43, 1011–1013. 10.1021/jo00399a052.

[ref7] Nicolaou K. C.; Zhong Y.-L.; Baran P. S. A new method for the one-step synthesis of α,β-unsaturated carbonyl systems from saturated alcohols and carbonyl compounds. J. Am. Chem. Soc. 2000, 122, 7596–7597. 10.1021/ja001825b.

[ref8] Diao T.; Wadzinski T. J.; Stahl S. S. Direct aerobic α,β-dehydrogenation of aldehydes and ketones with a Pd(TFA)_2_/4,5-diazafluorenone catalyst. Chem. Sci. 2012, 3, 887–891. 10.1039/C1SC00724F. 22690316 PMC3370690

[ref9] Gao W.; He Z.; Qian Y.; Zhao J.; Huang Y. General palladium-catalyzed aerobic dehydrogenation to generate double bonds. Chem. Sci. 2012, 3, 883–886. 10.1039/C1SC00661D.

[ref10] Wei Y.; Tang J.; Cong X.; Zeng X. Practical metal-free synthesis of chalcone derivatives via a tandem cross-dehydrogenative-coupling/elimination reaction. Green Chem. 2013, 15, 3165–3169. 10.1039/c3gc41403e.

[ref15] Zhang S.; Neumann H.; Beller M. Synthesis of α,β-unsaturated carbonyl compounds by carbonylation reactions. Chem. Soc. Rev. 2020, 49, 3187–3210. 10.1039/C9CS00615J. 32255444

[ref16] Goh K. K. K.; Kim S.; Zard S. Z. Free-radical variant for the synthesis of functionalized 1,5-diketones. Org. Lett. 2013, 15, 4818–4821. and the references cited therein 10.1021/ol402213k. 24011200

[ref17] Liu L.; Feng S.; Li C. A green synthesis of highly substituted 1,5-diketones. RSC Adv. 2015, 5, 56949–56953. and the references cited therein 10.1039/C5RA08682E.

[ref18] Vogler T.; Studer A. Applications of TEMPO in synthesis. Synthesis 2008, 2008, 1979–1993. 10.1055/s-2008-1078445.

[ref19] Molinder R.; Comyn T. P.; Hondow N.; Parker J. E.; Dupont V. In situ X-ray diffraction of CaO based CO_2_ sorbents. Energy Environ. Sci. 2012, 5, 8958–8969. 10.1039/c2ee21779a.

[ref20] aFile no. 23-0430 for Ca metal.

[ref21] aTas A. C. Calcium metal to synthesize amorphous or cryptocrystalline calcium phosphates. Mater. Sci. Eng., C 2012, 32, 1097–1106. 10.1016/j.msec.2012.01.024.

[ref22] Belekbir S.; El Azzouzi M.; El Hamidi A.; Rodríguez-Lorenzo L.; Santaballa J. A.; Canle M. Improved photocatalyzed degradation of phenol, as a model pollutant, over metal-impregnated nanosized TiO2. Nanomaterials 2020, 10, 996 10.3390/nano10050996. 32455949 PMC7279559

[ref23] Lawrinenko M.; Jing D.; Banik C.; Laird D. A. Aluminum and iron biomass pretreatment impacts on biochar anion exchange capacity. Carbon 2017, 118, 422–430. 10.1016/j.carbon.2017.03.056.

[ref24] Zumdahl S. S. Chemical Principles, 3rd ed.; Houghton Mifflin: New York, 1998.

[ref25] Kapoor M.; Hwu J. R. Na@SiO_2_-mediated addition of organohalides to carbonyl compounds for the formation of alcohols and epoxides. Sci. Rep. 2016, 6, 36225 10.1038/srep36225. 27853277 PMC5113255

[ref26] Zard S. Z. The xanthate route to ketones: when the radical is better than the enolate. Acc. Chem. Res. 2018, 51, 1722–1733. 10.1021/acs.accounts.8b00201. 29932322

[ref27] Liu D.; Liu C.; Lei A. Carbon-centered radical addition to C = X bonds for C–X bond formation. Chem.—Asian J. 2015, 10, 2040–2054. and references cited therein 10.1002/asia.201500326. 26011433

[ref28] Zimmerman H. E. A mechanistic analysis of the birch reduction. Acc. Chem. Res. 2012, 45, 164–170. 10.1021/ar2000698. 21923089

[ref29] Van Dort P. C.; Fuchs P. L. Free radical self-immolative 1,2-elimination and reductive desulfonylation of aryl sulfones promoted by intramolecular reactions with ortho-attached carbon-centered radicals. J. Org. Chem. 1997, 62, 7142–7147. 10.1021/jo970445e. 11671818

[ref30] Woźnica M.; Chaoui N.; Taabache S.; Blechert S. THF: an efficient electron donor in continuous flow radical cyclization photocatalyzed by graphitic carbon nitride. Chem.—Eur. J. 2014, 20, 14624–14628. 10.1002/chem.201404440. 25252017

[ref31] Kobayashi S.; Tamura T.; Yoshimoto S.; Kawakami T.; Masuyama A. 4-Methyltetrahydropyran (4-MeTHP): application as an organic reaction solvent. Chem.—Asian J. 2019, 14, 3921–3937. 10.1002/asia.201901169. 31549485 PMC6916367

[ref32] Lee S.; Lim C. J.; Kim S.; Subramaniam R.; Zimmerman J.; Sibi M. P. Enantioselective conjugate radical addition to α‘-hydroxy enones. Org. Lett. 2006, 8, 4311–4313. 10.1021/ol061634z. 16956214

[ref33] Le Saux E.; Ma D.; Bonilla P.; Holden C. M.; Lustosa D.; Melchiorre P. A general organocatalytic system for enantioselective radical conjugate additions to enals. Angew. Chem., Int. Ed. 2021, 60, 5357–5362. and references cited therein 10.1002/anie.202014876. PMC7986922 33283919

[ref34] Li C.-J.; Trost B. M. Green chemistry for chemical synthesis. Proc. Natl. Acad. Sci. U.S.A. 2008, 105, 13197–13202. 10.1073/pnas.0804348105. 18768813 PMC2533168

[ref35] Sheldon R. A. Atom efficiency and catalysis in organic synthesis. Pure Appl. Chem. 2000, 72, 1233–1246. 10.1351/pac200072071233.

[ref36] Trost B. M.; Oi S. Atom economy: aldol-type products by vanadium catalyzed additions of propargyl alcohols and aldehydes. J. Am. Chem. Soc. 2001, 123, 1230–1231. 10.1021/ja003629a. 11456678

[ref37] Anastas P.; Eghbali N. Green chemistry: principles and practice. Chem. Soc. Rev. 2010, 39, 301–312. 10.1039/B918763B. 20023854

