# Supplementary material for: Calcium Impregnated Silica Gel in the Domino Reaction Involving Irreversible Aldol Addition, Dehydration, and Michael Addition
Source: J Org Chem. 2025 Apr 16;90(16):5343–8. doi: 10.1021/acs.joc.4c02340 (PMC12038831; doi:10.1021/acs.joc.4c02340)
Supplement: Supplementary file 1 — jo4c02340_si_001.pdf [file jo4c02340_si_001.pdf]

Supporting Information

Calcium Impregnated Silica Gel in the Domino Reaction Involving Irreversible Aldol Addition, Dehydration, and Michael Addition

Jih Ru Hwu,*[,a] Khagendra Prasad Bohara,[a] Animesh Roy,[a] Wen-Chieh Huang,[a,b] Kuo-Chu Hwang,[a] Chun-Cheng Lin,[a] Kao Shu Chuang,[c] Shu-Yu Lin,[b] and Shwu-Chen Tsay[a]

[a]Department of Chemistry, and Frontier Research Center on Fundamental and Applied Sciences of Matters, National Tsing Hua University, Hsinchu 300, Taiwan;

[b]Institute of Biotechnology and Pharmaceutical Research, National Health Research Institutes, Miaoli County 350401, Taiwan;

[c]Department of Green Material Technology, Green Technology Research Institute, CPC Corporation, Kaohsiung City 81126, Taiwan

Jih Ru Hwu,*[,a] E-mail: jrhwu@mx.nthu.edu.tw

Contents

General Information S2

Preparation of Calcium Metal Impregnated Silica gel (Ca@SiO_2) S3

The Optimization of Reaction Conditions (Tables S1 and S2) S4

The Standard Procedure 1 for the Synthesis of Enones S5

The Standard Procedure 2 for the Synthesis of 1,5-Diketones S6

Experimental Data of α,β-Unsaturated Enones S6

Experimental Data of 1,5-Diketones S14

Calculation of Atom Economy and Atom Efficiency (Tables S3 and S4) S26

References S28

Spectra of Compounds S31

General Information

All reactions were carried out in oven-dried glassware (120 °C) under an atmosphere of nitrogen unless as indicated otherwise. Acetonitrile, ethyl acetate, and hexanes from Mallinckrodt Chemical Co. were dried and distilled from CaH_2. Diethyl ether (Et_2O) and tetrahydrofuran (THF) from Mallinckrodt Chemical Co. were dried by distillation from sodium and benzophenone under an atmosphere of nitrogen. 2-Methyltetrahydrofuran (2-MeTHF) from Sigma-Aldrich was dried by distillation from sodium and benzophenone under an atmosphere of nitrogen. The reagents purchased from Alfa Aesar included benzaldehyde, 4-chlorobenzaldehyde, 4-fluorobenzaldehyde, 4-methoxybenzaldehyde, methyl 4-formylbenzoate, and 4-nitrobenzaldehyde. The reagents purchased from Sigma-Aldrich included acetophenone, 4-acetylbenzonitrile, 2-acetylpyridine, 1,3-benzodioxole-5-carboxaldehyde, 2′-bromoacetophenone, 4′-bromoacetophenone, 3,4-dimethoxybenzaldehyde, 2-furancarboxaldehyde, 3′-methoxyacetophenone, 4′-methoxyacetophenone, 4′-methylacetophenone, 4-methylbenzaldehyde, 1-naphthaldehyde, and 2-thiophenecarboxaldehyde. The reagents purchased from Tokyo Chemical Industry Co. included 3′-bromoacetophenone, 4-bromobenzaldehyde, and 2-bromo-4,5-dimethoxybenzaldehyde. Calcium metal was purchased from Ferak Berlin.

Analytical thin-layer chromatography (TLC) was performed on precoated plates (silica gel 60 F-254). Purification by gravity column chromatography was carried out by use of Silicycle ultrapure silica gel (particle size 40–63 μm, 230–400 mesh).

Infrared (IR) spectra were recorded on a Fourier transform infrared (FT-IR) spectrometer. Absorption intensities are recorded by the following abbreviations: s, strong; m, medium; and w, weak. Proton NMR spectra were obtained on a 400 MHz spectrometer by use of chloroform-*d* ($CDCl_3$) as the solvent. Proton NMR chemical shifts were referenced to the residual protonated solvent (δ 7.24 ppm for chloroform). Carbon-13 NMR spectra were

obtained on a 100 MHz spectrometer by use of chloroform-*d* ($CDCl_3$) as the solvent. Carbon-13 chemical shifts were referenced to the center of the $CDCl_3$ triplet (δ 77.0 ppm). Multiplicities are recorded by the following abbreviations: s, singlet; d, doublet; dd, doublet of doublet; t, triplet; q, quartet; m, multiplet; and *J*, coupling constant (hertz). High-resolution mass spectra (HRMS) were measured on an instrument by use of a time-of-flight (TOF) mass analyzer with electrospray ionization (ESI).

Wide angle X-ray diffraction (XRD) was performed on a Bruker D8 Advance Eco X-ray powder diffractometer. A Cu-Kα radiation of 40kV, 20 mA was used with a scan speed of 0.5 °/min for a 2θ range from 10–70°.

Preparation of Calcium Metal Impregnated Silica gel (Ca@SiO_2). This calcium-based reagent was prepared in liquid ammonia at –78 °C. It differs from the powder mixture containing calcium metal and silica, which is prepared by mechanical ball milling inside a vial reactor at a high temperature (727–1727 °C).[1–3] Thus, calcium granules (8.21 g) and the oven-dried (110 °C for 4.0 h) silica gel (12.3 g, Geduran Si 60, particle size 40–63 μm, 230–400 mesh) were taken in a three-necked flask equipped with a magnetic stirring bar and rubber septa under argon atmosphere. To this flask was fitted a Dewar condenser equipped with a drying tube loaded with KOH pellets. The condenser and the flask were cooled to –78 °C by use of liquid nitrogen–acetone bath. Ammonia (g) was passed and condensed into the flask to dissolve calcium granules. After ammonia (~95 mL) was condensed, a blue slurry formed was stirred at –78°C for 30 min. The slurry was allowed to warm to room temperature with the simultaneous evaporation of ammonia under argon atmosphere. The traces of ammonia were then removed under reduced pressure (overnight) to afford grey colored free-flowing powders, which contained 40.0 wt% of calcium. This reagent was stored at room temperature for up to six months with reducing activity in an air-tight glass bottle. The same procedure was followed for the preparation of similar reagents containing different weight % of calcium in silica gel.

Caution: The entire procedure must be carefully performed under anhydrous conditions and in an argon atmosphere in a fume hood; otherwise, a fire might result.

The Optimization of Reaction Conditions

Table S1. Optimization of the reaction conditions[a] for 1a + 2o → 3ao in the presence of Ca@SiO$_2$.

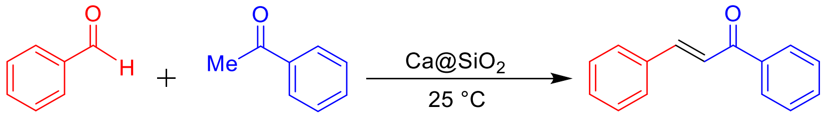

1a **2o** **3ao**

entry	solvent	Ca in Ca@SiO$_2$ (equiv)	Ca in Ca@SiO$_2$ (wt%)	yield[b] (%)
1	THF	0.0	40.0	0.0
2	THF	2.0	40.0	48
3	THF	2.5	40.0	70
4	THF	3.0	40.0	83
5	THF	3.5	40.0	83
6	2-MeTHF	2.0	40.0	49
7	2-MeTHF	2.5	40.0	71
8	2-MeTHF	3.0	30.0	65
9	2-MeTHF	3.0	40.0	85
10	2-MeTHF	3.0	50.0	60
11	2-MeTHF	3.5	40.0	84
12	CH_3CN	2.0	40.0	46
13	CH_3CN	2.5	40.0	68
14	CH_3CN	3.0	40.0	82
15	CH_3CN	3.5	40.0	82

[a]Conditions for the reaction: **1a** (1.0 equiv), **2o** (1.0 equiv), Ca@SiO$_2$ containing 30.0–50.0 wt% of Ca metal, reaction time for 18 h at 25 °C. [b]Isolated yield.

Table S2. Optimization of the reaction conditions[a] for 1a + 2o → 4ao in the presence of Ca@SiO

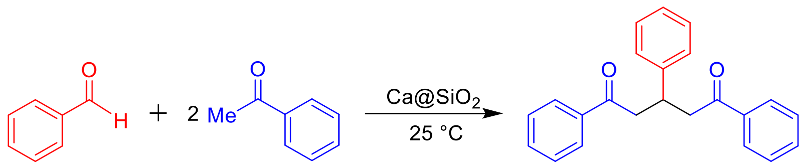

entry	solvent	Ca in $Ca@SiO_2$ (equiv)	Ca in $Ca@SiO_2$ (wt%)	yield[b] (%)
1	THF	0.0	40.0	0.0
2	THF	3.0	40.0	62
3	THF	3.5	40.0	73
4	THF	4.0	40.0	81
5	THF	4.5	40.0	81
6	2-MeTHF	3.0	40.0	64
7	2-MeTHF	3.5	40.0	74
8	2-MeTHF	4.0	30.0	61
9	2-MeTHF	4.0	40.0	82
10	2-MeTHF	4.5	40.0	82
11	2-MeTHF	4.0	50.0	58
12	CH_3CN	3.0	40.0	61
13	CH_3CN	3.5	40.0	71
14	CH_3CN	4.0	40.0	80
15	CH_3CN	4.5	40.0	79

[a]Conditions for the reaction: **1a** (1.0 equiv), **2o** (2.2 equiv), $Ca@SiO_2$ containing 30.0–50.0 wt% of Ca metal, reaction time for 20 h at 25 °C. [b]Isolated yield.

The Standard Procedure 1 for the Synthesis of Enones. A reaction flask was charged with $Ca@SiO_2$ (containing Ca 40.0 wt%, 3.0–3.2 equiv) under nitrogen atmosphere. To this reaction

mass was added 2-MeTHF (1.0–2.0 mL) via syringe at room temperature. Then a solution of an aldehyde **1** (1.0 equiv) and a ketone **2** (1.0 equiv) in 2-MeTHF (0.50–1.0 mL) was injected into the reaction flask. After the reaction mixture was stirred at 25 °C for 16–18 h, the inorganic residue was filtered and washed with EtOAc (3 × 3.0 mL). The combined filtrates were concentrated under reduced pressure. The crude product was then purified by use of column chromatography on silica gel with EtOAc in hexanes to give enones **3**.

The Standard Procedure 2 for the Synthesis of 1,5-Diketones. A reaction flask was charged with Ca@SiO_2 (containing Ca 40.0 wt%, 4.0–4.2 equiv) under nitrogen atmosphere. To this reaction mass was added 2-MeTHF (1.0–2.0 mL) via syringe at room temperature. Then a solution of an aldehyde **1** (1.0 equiv) and a ketone **2** (2.2 equiv) in 2-MeTHF (0.50–1.0 mL) was injected into the reaction flask. After the reaction mixture was stirred at 25 °C for 18–20 h, the inorganic residue was filtered and washed with EtOAc (3 × 3.0 mL). The combined filtrates were concentrated under reduced pressure. The crude product was then purified by use of column chromatography on silica gel with EtOAc in hexanes to give 1,5-diketones **4**.

Experimental Data of α,β-Unsaturated Enones

(*E*)-Chalcone (3ao). The standard procedure 1 was followed by use of Ca@SiO_2 (39.7 mg, 40.0 wt%, for Ca: 15.9 mg, 3.0 equiv) in 2-MeTHF (1.0 mL), benzaldehyde (**1a**, 13.9 mg, 0.131 mmol, 1.0 equiv), and acetophenone (**2o**, 15.7 mg, 0.131 mmol, 1.0 equiv) in 2-MeTHF (1.0 mL). After the reaction mixture was stirred at 25 °C for 18 h, the crude product was purified by use of column chromatography (2.0% EtOAc in hexanes as the eluent) to give the desired enone **3ao** (23.2 mg, 0.111 mmol) in 85% yield as pale yellow solids: mp (recrystallized from ethanol) 55.1–57.3 °C; TLC R_f 0.21 (5.0% EtOAc in hexanes as the eluent); ^{1}H NMR ($CDCl_3$, 400 MHz) δ 8.01 (d, J = 7.6 Hz, 2 H), 7.80 (d, J = 15.6 Hz, 1 H), 7.60–7.58

(m, 2 H), 7.55–7.43 (m, 4 H), 7.37–7.35 (m, 3 H); HRMS (ESI-TOF) *m/z:* $[M + H]^+$ Calcd for $C_{15}H_{13}O$ 209.0966; Found 209.0961. The spectroscopic data are in accordance with literature data.[4]

Gram-Scale Synthesis of 3ao.

The Standard Procedure 1 was followed by use of Ca@SiO_2 (2.98 g, 40.0 wt%, for Ca: 1.19 g, 3.0 equiv) in 2-MeTHF (45 mL), benzaldehyde (**1a**, 1.05 g, 9.89 mmol, 1.0 equiv), and acetophenone (**2o**, 1.19 g, 9.89 mmol, 1.0 equiv) in 2-MeTHF (45 mL). After the reaction mixture was stirred at 25 °C for 18 h, the crude product was purified by use of column chromatography (2.0% EtOAc in hexanes as the eluent) to give the desired enone **3ao** (1.71 g, 8.21 mmol) in 83% isolated yield.

Control Experiment in the Presence of TEMPO

A reaction flask was charged with Ca@SiO_2 (41.2 mg, 40.0 wt%, for Ca: 16.48 mg, 3.0 equiv) under nitrogen atmosphere. To this reaction mass was added 2-MeTHF (2.0 mL) via syringe at room temperature. After the reaction mass was stirred at 25 °C, (2,2,6,6-tetramethylpiperidin-1-yl)oxyl (TEMPO) (63.8 mg, 0.408 mmol, 3.0 equiv) in 2-MeTHF (1.0 mL) was injected into the reaction flask. Then a solution of benzaldehyde (**1a**, 14.5 mg, 0.136 mmol, 1.0 equiv), and acetophenone (**2o**, 16.4 mg, 0.136 mmol, 1.0 equiv) in 2-MeTHF (1.0 mL) was injected into the reaction flask. After the reaction mixture was stirred at 25 °C for 18 h, the inorganic residue was filtered and washed with EtOAc (3 × 3.0 mL). The combined filtrates were concentrated under reduced pressure. The TLC was monitored (5.0% EtOAc in hexanes as the eluent) to detect the completion of reaction. The resultant mixture contained many components as checked by GC. Nevertheless, desired enone **3ao** was not detected.

(*E*)-Methyl 4-(3-Oxo-3-phenylprop-1-en-1-yl)benzoate (3ho). The standard procedure 1 was followed by use of Ca@SiO_2 (51.6 mg, 40.0 wt%, for Ca: 20.6 mg, 3.2 equiv) in 2-MeTHF (1.5 mL), methyl 4-formylbenzoate (**1h**, 26.5 mg, 0.161 mmol, 1.0 equiv), and acetophenone (**2o**, 19.4 mg, 0.161 mmol, 1.0 equiv) in 2-MeTHF (1.0 mL). After the reaction mixture was stirred at 25 °C for 17 h, the crude product was purified by use of column chromatography (10% EtOAc in hexanes as the eluent) to give the desired enone **3ho** (32.6 mg, 0.122 mmol) in 76% yield as light yellow solids: mp (recrystallized from EtOAc/hexanes) 127.1–128.4 °C; TLC R_f 0.45 (20% EtOAc in hexanes as the eluent); ^{1}H NMR ($CDCl_3$, 400 MHz) δ 8.06 (d, J = 8.0 Hz, 2 H), 8.01 (d, J = 7.2 Hz, 2 H), 7.79 (d, J = 15.6 Hz, 1 H), 7.68 (d, J = 8.4 Hz, 2 H), 7.60–7.56 (m, 2 H), 7.50 (t, J = 7.6 Hz, 2 H), 3.92 (s, 3 H); HRMS (ESI-TOF) *m/z:* $[M + H]^+$ Calcd for $C_{17}H_{15}O_3$ 267.1021; Found 267.1020. The spectroscopic data are in accordance with literature data.[5]

(*E*)-1-(4-Methylphenyl)-3-phenylprop-2-en-1-one (3ap). The standard procedure 1 was followed by use of Ca@SiO_2 (41.3 mg, 40.0 wt%, for Ca: 16.5 mg, 3.1 equiv) in 2-MeTHF (1.0 mL), benzaldehyde (**1a**, 14.1 mg, 0.133 mmol, 1.0 equiv), and 4′-methylacetophenone (**2p**, 17.8 mg, 0.133 mmol, 1.0 equiv) in 2-MeTHF (1.0 mL). After the reaction mixture was stirred at 25 °C for 17 h, the crude product was purified by use of column chromatography (5.0% EtOAc in hexanes as the eluent) to give the desired enone **3ap** (25.4 mg, 0.114 mmol) in 86% yield as a colorless oil: TLC R_f 0.34 (10% EtOAc in hexanes as the eluent); ^{1}H NMR ($CDCl_3$, 400 MHz) δ 7.92 (d, J = 8.0 Hz, 2 H), 7.78 (d, J = 15.6 Hz, 1 H), 7.64–7.61 (m, 2 H), 7.52 (d, J = 15.6 Hz, 1 H), 7.40–7.39 (m, 3 H), 7.29 (d, J = 8.4 Hz, 2 H), 2.42 (s, 3 H); HRMS (ESI-TOF) *m/z:* $[M + H]^+$ Calcd for $C_{16}H_{15}O$ 223.1122; Found 223.1122. The spectroscopic data are in accordance with literature data.[6]

(*E*)-1-(3-Methoxyphenyl)-3-phenylprop-2-en-1-one (3aq). The standard procedure 1 was followed by use of Ca@SiO_2 (54.4 mg, 40.0 wt%, for Ca: 21.8 mg, 3.1 equiv) in 2-MeTHF (1.5 mL), benzaldehyde (**1a**, 18.6 mg, 0.175 mmol, 1.0 equiv), and 3′-methoxyacetophenone (**2q**, 26.3 mg, 0.175 mmol, 1.0 equiv) in 2-MeTHF (1.0 mL). After the reaction mixture was stirred at 25 °C for 16 h, the crude product was purified by use of column chromatography (7.0% EtOAc in hexanes as the eluent) to give the desired enone **3aq** (29.8 mg, 0.125 mmol) in 71% yield as a colorless oil: TLC R_f 0.32 (20% EtOAc in hexanes as the eluent); ^{1}H NMR ($CDCl_3$, 400 MHz) δ 7.79 (d, J = 15.6 Hz, 1 H), 7.64–7.57 (m, 3 H), 7.53–7.47 (m, 2 H), 7.41–7.37 (m, 4 H), 7.13–7.10 (m, 1 H), 3.87 (s, 3 H); HRMS (ESI-TOF) *m/z:* $[M + H]^+$ Calcd for $C_{16}H_{15}O_2$ 239.1072; Found 239.1073. The spectroscopic data are in accordance with literature data.[7]

(*E*)-1-(2-Bromophenyl)-3-phenylprop-2-en-1-one (3as). The standard procedure 1 was followed by use of Ca@SiO_2 (66.8 mg, 40.0 wt%, for Ca: 26.7 mg, 3.1 equiv) in 2-MeTHF (1.0 mL), benzaldehyde (**1a**, 22.8 mg, 0.215 mmol, 1.0 equiv), and 2′-bromoacetophenone (**2s**, 42.8 mg, 0.215 mmol, 1.0 equiv) in 2-MeTHF (1.0 mL). After the reaction mixture was stirred at 25 °C for 16 h, the crude product was purified by use of column chromatography (10% EtOAc in hexanes as the eluent) to give the desired enone **3as** (55.5 mg, 0.193 mmol) in 90% yield as a pale yellow oil: TLC R_f 0.35 (10% EtOAc in hexanes as the eluent); ^{1}H NMR ($CDCl_3$, 400 MHz) δ 7.62 (d, J = 8.0 Hz, 1 H), 7.54–7.52 (m, 2 H), 7.43–7.29 (m, 7 H), 7.08 (d, J = 16.4 Hz, 1 H); HRMS (ESI-TOF) *m/z:* $[M + H]^+$ Calcd for $C_{15}H_{12}BrO$ 287.0071; Found 287.0075. The spectroscopic data are in accordance with literature data.[8]

(*E*)-1-(2-Bromophenyl)-3-(*p*-tolyl)prop-2-en-1-one (3bs). The standard procedure 1 was followed by use of Ca@SiO_2 (76.3 mg, 40.0 wt%, for Ca: 30.5 mg, 3.0 equiv) in 2-MeTHF

(1.5 mL), 4-methylbenzaldehyde (**1b**, 30.3 mg, 0.252 mmol, 1.0 equiv), and 2′-bromoacetophenone (**2s**, 50.2 mg, 0.252 mmol, 1.0 equiv) in 2-MeTHF (1.0 mL). After the reaction mixture was stirred at 25 °C for 18 h, the crude product was purified by use of column chromatography (12% EtOAc in hexanes as the eluent) to give the desired enone **3bs** (65.9 mg, 0.219 mmol) in 87% yield as a colorless sticky oil: TLC R_f 0.42 (15% EtOAc in hexanes as the eluent); ^{1}H NMR ($CDCl_3$, 400 MHz) δ 7.62 (d, J = 8.0 Hz, 1 H), 7.44–7.35 (m, 5 H), 7.33–7.28 (m, 1 H), 7.18 (d, J = 7.6 Hz, 2 H), 7.03 (d, J = 16.0 Hz, 1 H), 2.36 (s, 3 H); HRMS (ESI-TOF) *m/z:* $[M + H]^+$ Calcd for $C_{16}H_{14}BrO$ 301.0228; Found 301.0227. The spectroscopic data are in accordance with literature data.[9]

(*E*)-1-(2-Bromophenyl)-3-(4-methoxyphenyl)prop-2-en-1-one (3cs). The standard procedure 1 was followed by use of Ca@SiO_2 (59.4 mg, 40.0 wt%, for Ca: 23.8 mg, 3.2 equiv) in 2-MeTHF (1.0 mL), 4-methoxybenzaldehyde (**1c**, 25.2 mg, 0.185 mmol, 1.0 equiv), and 2′-bromoacetophenone (**2s**, 36.9 mg, 0.185 mmol, 1.0 equiv) in 2-MeTHF (1.0 mL). After the reaction mixture was stirred at 25 °C for 18 h, the crude product was purified by use of column chromatography (10% EtOAc in hexanes as the eluent) to give the desired enone **3cs** (51.8 mg, 0.163 mmol) in 88% yield as pale yellow solids: mp (recrystallized from ethanol) 89.1–90.3 °C; TLC R_f 0.29 (15% EtOAc in hexanes as the eluent); ^{1}H NMR ($CDCl_3$, 400 MHz) δ 7.61 (d, J = 8.0 Hz, 1 H), 7.49 (d, J = 8.8 Hz, 2 H), 7.39–7.27 (m, 4 H), 6.94 (d, J = 16.0 Hz, 1 H), 6.89 (d, J = 8.8 Hz, 2 H), 3.82 (s, 3 H); HRMS (ESI-TOF) *m/z:* $[M + H]^+$ Calcd for $C_{16}H_{14}BrO_2$ 317.0177; Found 317.0172. The spectroscopic data are in accordance with literature data.[10]

(*E*)-3-(3,4-Dimethoxyphenyl)-1-phenylprop-2-en-1-one (3io). The standard procedure 1 was followed by use of Ca@SiO_2 (67.3 mg, 40.0 wt%, for Ca: 26.9 mg, 3.1 equiv) in 2-MeTHF (2.0 mL), 3,4-dimethoxybenzaldehyde (**1i**, 35.8 mg, 0.215 mmol, 1.0 equiv), and acetophenone

(**2o**, 25.8 mg, 0.215 mmol, 1.0 equiv) in 2-MeTHF (1.0 mL). After the reaction mixture was stirred at 25 °C for 18 h, the crude product was purified by use of column chromatography (20% EtOAc in hexanes as the eluent) to give the desired enone **3io** (45.4 mg, 0.169 mmol) in 79% yield as pale yellow solids: mp (recrystallized from EtOH) 94.1–95.4 °C; TLC R_f 0.37 (40% EtOAc in hexanes as the eluent); ^{1}H NMR ($CDCl_3$, 400 MHz) δ 7.98 (d, J = 7.2 Hz, 2 H), 7.74 (d, J = 15.6 Hz, 1 H), 7.55 (t, J = 7.0 Hz, 1 H), 7.47 (t, J = 7.4 Hz, 2 H), 7.36 (d, J = 15.6 Hz, 1 H), 7.21 (d, J = 8.0 Hz, 1 H), 7.14 (s, 1 H), 6.87 (d, J = 8.0 Hz, 1 H), 3.92 (s, 3 H), 3.90 (s, 3 H); HRMS (ESI-TOF) *m/z:* $[M + H]^+$ Calcd for $C_{17}H_{17}O_3$ 269.1177; Found 269.1174. The spectroscopic data are in accordance with literature data.[11]

(*E*)-3-(2-Bromo-4,5-dimethoxyphenyl)-1-(4-bromophenyl)prop-2-en-1-one (3ku). The standard procedure 1 was followed by use of Ca@SiO_2 (46.1 mg, 40.0 wt%, for Ca: 18.4 mg, 3.0 equiv) in 2-MeTHF (2.0 mL), 2-bromo-4,5-dimethoxybenzaldehyde (**1k**, 37.3 mg, 0.152 mmol, 1.0 equiv), and 4′-bromoacetophenone (**2u**, 30.3 mg, 0.152 mmol, 1.0 equiv) in 2-MeTHF (1.0 mL). After the reaction mixture was stirred at 25 °C for 17 h, the crude product was purified by use of column chromatography (22% EtOAc in hexanes as the eluent) to give the desired enone **3ku** (46.2 mg, 0.108 mmol) in 71% yield as a pale yellow viscous oil: TLC R_f 0.32 (40% EtOAc in hexanes as the eluent); ^{1}H NMR ($CDCl_3$, 400 MHz) δ 8.01 (d, J = 15.6 Hz, 1 H), 7.80 (d, J = 8.4 Hz, 2 H), 7.57 (d, J = 8.4 Hz, 2 H), 7.19 (d, J = 15.6 Hz, 1 H), 7.12 (s, 1 H), 7.02 (s, 1 H), 3.90 (s, 3 H), 3.86 (s, 3 H); HRMS (ESI-TOF) *m/z:* $[M + Na]^+$ Calcd for $C_{17}H_{14}Br_2O_3Na$ 446.9207; Found 446.9205. The spectroscopic data are in accordance with literature data.[12]

(*E*)-3-(1,3-Benzodioxol-5-yl)-1-phenylprop-2-en-1-one (3jo). The standard procedure 1 was followed by use of Ca@SiO_2 (78.6 mg, 40.0 wt%, for Ca: 31.4 mg, 3.2 equiv) in 2-MeTHF

(1.5 mL), 1,3-benzodioxole-5-carboxaldehyde (**1j**, 36.8 mg, 0.245 mmol, 1.0 equiv), and acetophenone (**2o**, 29.4 mg, 0.245 mmol, 1.0 equiv) in 2-MeTHF (1.0 mL). After the reaction mixture was stirred at 25 °C for 18 h, the crude product was purified by use of column chromatography (12% EtOAc in hexanes as the eluent) to give the desired enone **3jo** (52.8 mg, 0.209 mmol) in 85% yield as pale yellow solids: mp (recrystallized from EtOAc/hexanes) 106.2–107.4 °C; TLC R_f 0.47 (25% EtOAc in hexanes as the eluent); ^{1}H NMR ($CDCl_3$, 400 MHz) δ 7.97 (d, J = 7.2 Hz, 2 H), 7.70 (d, J = 15.6 Hz, 1 H), 7.54 (t, J = 7.4 Hz, 1 H), 7.46 (t, J = 7.4 Hz, 2 H), 7.34 (d, J = 15.6 Hz, 1 H), 7.13 (s, 1 H), 7.08 (d, J = 8.0 Hz, 1 H), 6.80 (d, J = 8.0 Hz, 1 H), 5.98 (s, 2 H); HRMS (ESI-TOF) *m/z:* $[M + H]^+$ Calcd for $C_{16}H_{13}O_3$ 253.0864; Found 253.0866. The spectroscopic data are in accordance with literature data.[13]

(*E*)-3-(Naphthalen-1-yl)-1-phenylprop-2-en-1-one (3lo). The standard procedure 1 was followed by use of Ca@SiO_2 (94.5 mg, 40.0 wt%, for Ca: 37.8 mg, 3.1 equiv) in 2-MeTHF (2.0 mL), 1-naphthaldehyde (**1l**, 47.2 mg, 0.302 mmol, 1.0 equiv), and acetophenone (**2o**, 36.3 mg, 0.302 mmol, 1.0 equiv) in 2-MeTHF (0.50 mL). After the reaction mixture was stirred at 25 °C for 16 h, the crude product was purified by use of column chromatography (7.0% EtOAc in hexanes as the eluent) to give the desired enone **3lo** (61.8 mg, 0.239 mmol) in 79% yield as yellow solids: mp (recrystallized from EtOH) 78.2–80.3 °C; TLC R_f 0.56 (10% EtOAc in hexanes as the eluent); ^{1}H NMR ($CDCl_3$, 400 MHz) δ 8.67 (d, J = 15.6 Hz, 1 H), 8.24 (d, J = 8.4 Hz, 1 H), 8.07 (d, J = 7.2 Hz, 2 H), 7.91–7.86 (m, 3 H), 7.63–7.48 (m, 7 H); HRMS (ESI-TOF) *m/z:* $[M + H]^+$ Calcd for $C_{19}H_{15}O$ 259.1122; Found 259.1127. The spectroscopic data are in accordance with literature data.[14]

(*E*)-3-(Furan-2-yl)-1-phenylprop-2-en-1-one (3mo). The standard procedure 1 was followed by use of Ca@SiO_2 (106 mg, 40.0 wt%, for Ca: 42.4 mg, 3.2 equiv) in 2-MeTHF (1.0 mL), 2-

furancarboxaldehyde (**1m**, 31.9 mg, 0.332 mmol, 1.0 equiv), and acetophenone (**2o**, 39.9 mg, 0.332 mmol, 1.0 equiv) in 2-MeTHF (1.0 mL). After the reaction mixture was stirred at 25 °C for 16 h, the crude product was purified by use of column chromatography (8.0% EtOAc in hexanes as the eluent) to give the desired enone **3mo** (53.4 mg, 0.269 mmol) in 81% yield as a pale yellow oil: TLC R_f 0.67 (15% EtOAc in hexanes as the eluent); ^{1}H NMR ($CDCl_3$, 400 MHz) δ 8.01 (d, J = 8.0 Hz, 2 H), 7.59–7.46 (m, 6 H), 6.70 (d, J = 3.2 Hz, 1 H), 6.49 (t, J = 1.6 Hz, 1 H); HRMS (ESI-TOF) *m/z:* $[M + H]^+$ Calcd for $C_{13}H_{11}O_2$ 199.0759; Found 199.0758. The spectroscopic data are in accordance with literature data.[8]

(*E*)-1-Phenyl-3-(thiophen-2-yl)prop-2-en-1-one (3no). The standard procedure 1 was followed by use of Ca@SiO_2 (94.1 mg, 40.0 wt%, for Ca: 37.6 mg, 3.1 equiv) in 2-MeTHF (2.0 mL), 2-thiophenecarboxaldehyde (**1n**, 33.8 mg, 0.301 mmol, 1.0 equiv), and acetophenone (**2o**, 36.2 mg, 0.301 mmol, 1.0 equiv) in 2-MeTHF (1.0 mL). After the reaction mixture was stirred at 25 °C for 17 h, the crude product was purified by use of column chromatography (6.0% EtOAc in hexanes as the eluent) to give the desired enone **3no** (49.1 mg, 0.229 mmol) in 76% yield as yellow solids: mp (recrystallized from EtOAc/hexanes) 42.6–44.2 °C; TLC R_f 0.50 (10% EtOAc in hexanes as the eluent); ^{1}H NMR ($CDCl_3$, 400 MHz) δ 7.98 (d, J = 7.6 Hz, 2 H), 7.92 (d, J = 15.6 Hz, 1 H), 7.55 (t, J = 7.4 Hz, 1 H), 7.47 (t, J = 7.4 Hz, 2 H), 7.39 (d, J = 4.8 Hz, 1 H), 7.33–7.29 (m, 2 H), 7.07–7.05 (m, 1 H); HRMS (ESI-TOF) *m/z:* $[M + H]^+$ Calcd for $C_{13}H_{11}OS$ 215.0530; Found 215.0537. The spectroscopic data are in accordance with literature data.[8]

(*E*)-3-Phenyl-1-(pyridine-2-yl)prop-2-en-1-one (3aw). The standard procedure 1 was followed by use of Ca@SiO_2 (94.4 mg, 40.0 wt%, for Ca: 37.8 mg, 3.0 equiv) in 2-MeTHF (2.0 mL), benzaldehyde (**1a**, 33.1 mg, 0.312 mmol, 1.0 equiv), and 2-acetylpyridine (**2w**, 37.8

mg, 0.312 mmol, 1.0 equiv) in 2-MeTHF (0.50 mL). After the reaction mixture was stirred at 25 °C for 18 h, the crude product was purified by use of column chromatography (15% EtOAc in hexanes as the eluent) to give the desired enone **3aw** (49.2 mg, 0.235 mmol) in 75% yield as yellow solids: mp (recrystallized from EtOAc/hexanes) 69.6–71.8 °C; TLC R_f 0.44 (20% EtOAc in hexanes as the eluent); ^{1}H NMR ($CDCl_3$, 400 MHz) δ 8.72 (d, J = 4.4 Hz, 1 H), 8.29 (d, J = 16.0 Hz, 1 H), 8.17 (d, J = 8.0 Hz, 1 H), 7.92 (d, J = 16.0 Hz, 1 H), 7.85 (t, J = 7.8 Hz, 1 H), 7.72–7.70 (m, 2 H), 7.48–7.39 (m, 4 H); HRMS (ESI-TOF) *m/z:* $[M + H]^+$ Calcd for $C_{14}H_{12}NO$ 210.0918; Found 210.0913. The spectroscopic data are in accordance with literature data.[14]

Experimental Data of 1,5-Diones

1,3,5-Triphenylpentane-1,5-dione (4ao). The standard procedure 2 was followed by use of Ca@SiO_2 (76.2 mg, 40.0 wt%, for Ca: 30.5 mg, 4.0 equiv) in 2-MeTHF (1.0 mL), benzaldehyde (**1a**, 20.1 mg, 0.189 mmol, 1.0 equiv), and acetophenone (**2o**, 50.1 mg, 0.417 mmol, 2.2 equiv) in 2-MeTHF (1.0 mL). After the reaction mixture was stirred at 25 °C for 20 h, the crude product was purified by use of column chromatography (8.0% EtOAc in hexanes as the eluent) to give the desired 1,5-diketone **4ao** (51.1 mg, 0.155 mmol) in 82% yield as colorless solids: mp (recrystallized from EtOAc/hexanes) 86.2–88.7 °C; TLC R_f 0.51 (20% EtOAc in hexanes as the eluent); ^{1}H NMR ($CDCl_3$, 400 MHz) δ 7.93 (d, J = 7.6 Hz, 4 H), 7.52 (t, J = 7.4 Hz, 2 H), 7.44–7.40 (m, 4 H), 7.27–7.22 (m, 4 H), 7.18–7.14 (m, 1 H), 4.10–4.03 (m, 1 H), 3.48 (dd, J = 16.8 Hz, 7.2 Hz, 2 H), 3.34 (dd, J = 16.8 Hz, 7.2 Hz, 2 H); HRMS (ESI-TOF) *m/z:* $[M + Na]^+$ Calcd for $C_{23}H_{20}O_2Na$ 351.1361; Found 351.1361. The spectroscopic data are in accordance with literature data.[15]

Gram-Scale Synthesis of 4ao.

The Standard Procedure 2 was followed by use of Ca@SiO_2 (4.05 g, 40.0 wt%, for Ca: 1.62 g, 4.0 equiv) in 2-MeTHF (50 mL), benzaldehyde (**1a**, 1.06 g, 9.98 mmol, 1.0 equiv), and acetophenone (**2o**, 2.64 g, 21.9 mmol, 2.2 equiv) in 2-MeTHF (50 mL). After the reaction mixture was stirred at 25 °C for 20 h, the crude product was purified by use of column chromatography (8.0% EtOAc in hexanes as the eluent) to give the desired 1,5-diketone **4ao** (2.73 g, 8.31 mmol) in 83% isolated yield.

1,5-Diphenyl-3-*p*-tolylpentane-1,5-dione (4bo). The standard procedure 2 was followed by use of Ca@SiO_2 (85.3 mg, 40.0 wt%, for Ca: 34.1 mg, 4.2 equiv) in 2-MeTHF (2.0 mL), 4-methylbenzaldehyde (**1b**, 24.2 mg, 0.201 mmol, 1.0 equiv), and acetophenone (**2o**, 53.3 mg, 0.444 mmol, 2.2 equiv) in 2-MeTHF (0.50 mL). After the reaction mixture was stirred at 25 °C for 20 h, the crude product was purified by use of column chromatography (6.0% EtOAc in hexanes as the eluent) to give the desired 1,5-diketone **4bo** (54.5 mg, 0.159 mmol) in 79% yield as white solids: mp (recrystallized from EtOH) 101.2–102.3 °C; TLC R_f 0.42 (20% EtOAc in hexanes as the eluent); ^1H NMR ($CDCl_3$, 400 MHz) δ 7.95 (d, J = 7.2 Hz, 4 H), 7.53 (t, J = 7.4 Hz, 2 H), 7.43 (t, J = 7.8 Hz, 4 H), 7.17 (d, J = 8.4 Hz, 2 H), 7.08 (d, J = 8.0 Hz, 2 H), 4.07–4.00 (m, 1 H), 3.48 (dd, J = 16.8 Hz, 7.2 Hz, 2 H), 3.32 (dd, J = 16.8 Hz, 7.2 Hz, 2 H), 2.28 (s, 3 H); HRMS (ESI-TOF) *m/z:* $[M + Na]^+$ Calcd for $C_{24}H_{22}O_2Na$ 365.1517; Found 365.1522. The spectroscopic data are in accordance with literature data.[16]

3-(4-Methoxyphenyl)-1,5-diphenylpentane-1,5-dione (4co). The standard procedure 2 was followed by use of Ca@SiO_2 (83.8 mg, 40.0 wt%, for Ca: 33.5 mg, 4.1 equiv) in 2-MeTHF (1.5 mL), 4-methoxybenzaldehyde (**1c**, 27.7 mg, 0.203 mmol, 1.0 equiv), and acetophenone (**2o**, 53.8 mg, 0.448 mmol, 2.2 equiv) in 2-MeTHF (0.05 mL). After the reaction mixture was

stirred at 25 °C for 20 h, the crude product was purified by use of column chromatography (12% EtOAc in hexanes as the eluent) to give the desired 1,5-diketone **4co** (56.3 mg, 0.157 mmol) in 77% yield as white solids: mp (recrystallized from EtOH) 98.6–99.8 °C; TLC R_f 0.34 (20% EtOAc in hexanes as the eluent); ^{1}H NMR ($CDCl_3$, 400 MHz) δ 7.93 (d, J = 8.0 Hz, 4 H), 7.52 (t, J = 7.2 Hz, 2 H), 7.42 (t, J = 7.8 Hz, 4 H), 7.17 (d, J = 8.4 Hz, 2 H), 6.79 (d, J = 8.0 Hz, 2 H), 4.04–3.97 (m, 1 H), 3.73 (s, 3 H), 3.45 (dd, J = 16.8 Hz, 7.2 Hz, 2 H), 3.29 (dd, J = 16.4 Hz, 7.2 Hz, 2 H); HRMS (ESI-TOF) *m/z:* $[M + H]^+$ Calcd for $C_{24}H_{23}O_3$ 359.1647; Found 359.1642. The spectroscopic data are in accordance with literature data.[16]

3-(4-Fluorophenyl)-1,5-diphenylpentane-1,5-dione (4do). The standard procedure 2 was followed by use of Ca@SiO_2 (83.5 mg, 40.0 wt%, for Ca: 33.4 mg, 4.2 equiv) in 2-MeTHF (1.0 mL), 4-fluorobenzaldehyde (**1d**, 24.4 mg, 0.197 mmol, 1.0 equiv), and acetophenone (**2o**, 52.1 mg, 0.434 mmol, 2.2 equiv) in 2-MeTHF (1.0 mL). After the reaction mixture was stirred at 25 °C for 18 h, the crude product was purified by use of column chromatography (7.0% EtOAc in hexanes as the eluent) to give the desired 1,5-diketone **4do** (60.1 mg, 0.174 mmol) in 88% yield as white solids: mp (recrystallized from EtOAc/hexanes) 67.6–68.8 °C; TLC R_f 0.32 (10% EtOAc in hexanes as the eluent); ^{1}H NMR ($CDCl_3$, 400 MHz) δ 7.92 (d, J = 7.6 Hz, 4 H), 7.53 (t, J = 7.4 Hz, 2 H), 7.42 (t, J = 7.6 Hz, 4 H), 7.24–7.21 (m, 2 H), 6.93 (t, J = 8.6 Hz, 2 H), 4.08–4.01 (m, 1 H), 3.45 (dd, J = 16.8 Hz, 6.8 Hz, 2 H), 3.30 (dd, J = 16.8 Hz, 7.2 Hz, 2 H); HRMS (ESI-TOF) *m/z:* $[M + H]^+$ Calcd for $C_{23}H_{20}FO_2$ 347.1447; Found 347.1439. The spectroscopic data are in accordance with literature data.[17]

3-(4-Chlorophenyl)-1,5-diphenylpentane-1,5-dione (4eo). The standard procedure 2 was followed by use of Ca@SiO_2 (77.8 mg, 40.0 wt%, for Ca: 31.1 mg, 4.1 equiv) in 2-MeTHF (1.5 mL), 4-chlorobenzaldehyde (**1e**, 26.4 mg, 0.188 mmol, 1.0 equiv), and acetophenone (**2o**,

49.8 mg, 0.414 mmol, 2.2 equiv) in 2-MeTHF (1.0 mL). After the reaction mixture was stirred at 25 °C for 19 h, the crude product was purified by use of column chromatography (8.0% EtOAc in hexanes as the eluent) to give the desired 1,5-diketone **4eo** (49.8 mg, 0.137 mmol) in 73% yield as white solids: mp (recrystallized from EtOAc/hexanes) 96.3–97.6 °C; TLC R_f 0.57 (20% EtOAc in hexanes as the eluent); ^{1}H NMR ($CDCl_3$, 400 MHz) δ 7.91 (d, J = 7.2 Hz, 4 H), 7.53 (t, J = 7.4 Hz, 2 H), 7.42 (t, J = 7.6 Hz, 4 H), 7.22 (d, J = 10.8 Hz, 4 H), 4.07–4.00 (m, 1 H), 3.46 (dd, J = 16.8 Hz, 6.8 Hz, 2 H), 3.30 (dd, J = 16.8 Hz, 7.2 Hz, 2 H); HRMS (ESI-TOF) *m/z:* $[M + H]^+$ Calcd for $C_{23}H_{20}ClO_2$ 363.1151; Found 363.1141. The spectroscopic data are in accordance with literature data.[15]

3-(4-Bromophenyl)-1,5-diphenylpentane-1,5-dione (4fo). The standard procedure 2 was followed by use of Ca@SiO_2 (51.8 mg, 40.0 wt%, for Ca: 20.7 mg, 4.2 equiv) in 2-MeTHF (2.0 mL), 4-bromobenzaldehyde (**1f**, 22.6 mg, 0.122 mmol, 1.0 equiv), and acetophenone (**2o**, 32.6 mg, 0.271 mmol, 2.2 equiv) in 2-MeTHF (1.0 mL). After the reaction mixture was stirred at 25 °C for 19 h, the crude product was purified by use of column chromatography (10% EtOAc in hexanes as the eluent) to give the desired 1,5-diketone **4fo** (42.4 mg, 0.104 mmol) in 85% yield as white solids: mp (recrystallized from EtOAc/hexanes) 92.2–93.4 °C; TLC R_f 0.52 (20% EtOAc in hexanes as the eluent); ^{1}H NMR ($CDCl_3$, 400 MHz) δ 7.91 (d, J = 7.2 Hz, 4 H), 7.53 (t, J = 7.4 Hz, 2 H), 7.42 (t, J = 7.6 Hz, 4 H), 7.37 (d, J = 8.4 Hz, 2 H), 7.15 (d, J = 8.4 Hz, 2 H), 4.06–3.99 (m, 1 H), 3.46 (dd, J = 16.8 Hz, 6.8 Hz, 2 H), 3.30 (dd, J = 16.8 Hz, 7.2 Hz, 2 H); HRMS (ESI-TOF) *m/z:* $[M + H]^+$ Calcd for $C_{23}H_{20}BrO_2$ 407.0646; Found 407.0648. The spectroscopic data are in accordance with literature data.[16]

3-(4-Nitrophenyl)-1,5-diphenylpentane-1,5-dione (4go). The standard procedure 2 was followed by use of Ca@SiO_2 (67.8 mg, 40.0 wt%, for Ca: 27.1 mg, 4.1 equiv) in 2-MeTHF

(2.0 mL), 4-nitrobenzaldehyde (**1g**, 24.8 mg, 0.164 mmol, 1.0 equiv), and acetophenone (**2o**, 43.8 mg, 0.365 mmol, 2.2 equiv) in 2-MeTHF (1.0 mL). After the reaction mixture was stirred at 25 °C for 18 h, the crude product was purified by use of column chromatography (20% EtOAc in hexanes as the eluent) to give the desired 1,5-diketone **4go** (45.2 mg, 0.121 mmol) in 73% yield as a light yellow oil: TLC R_f 0.24 (30% EtOAc in hexanes as the eluent); ^{1}H NMR ($CDCl_3$, 400 MHz) δ 8.12 (d, J = 8.8 Hz, 2 H), 7.91 (d, J = 8.8 Hz, 4 H), 7.55 (t, J = 7.4 Hz, 2 H), 7.48–7.42 (m, 6 H), 4.22–4.15 (m, 1 H), 3.53 (dd, J = 17.2 Hz, 6.8 Hz, 2 H), 3.38 (dd, J = 17.2 Hz, 7.6 Hz, 2 H); HRMS (ESI-TOF) *m/z:* $[M + H]^+$ Calcd for $C_{23}H_{20}NO_4$ 374.1392; Found 374.1389. The spectroscopic data are in accordance with literature data.[18]

Methyl 4-(1,5-Dioxo-1,5-diphenylpentan-3-yl)benzoate (4ho). The standard procedure 2 was followed by use of Ca@SiO_2 (58.5 mg, 40.0 wt%, for Ca: 23.4 mg, 4.2 equiv) in 2-MeTHF (1.5 mL), methyl 4-formylbenzoate (**1h**, 22.6 mg, 0.138 mmol, 1.0 equiv), and acetophenone (**2o**, 36.6 mg, 0.305 mmol, 2.2 equiv) in 2-MeTHF (1.0 mL). After the reaction mixture was stirred at 25 °C for 18 h, the crude product was purified by use of column chromatography (15% EtOAc in hexanes as the eluent) to give the desired 1,5-diketone **4ho** (40.6 mg, 0.105 mmol) in 76% yield as white solids: mp (recrystallized from EtOAc/hexanes) 95.4–96.8 °C; TLC R_f 0.19 (20% EtOAc in hexanes as the eluent); ^{1}H NMR ($CDCl_3$, 400 MHz) δ 7.92 (t, J = 7.2 Hz, 6 H), 7.53 (t, J = 7.2 Hz, 2 H), 7.42 (t, J = 7.6 Hz, 4 H), 7.35 (d, J = 8.0 Hz, 2 H), 4.16–4.09 (m, 1 H), 3.85 (s, 3 H), 3.49 (dd, J = 16.8 Hz, 6.8 Hz, 2 H), 3.35 (dd, J = 16.8 Hz, 7.2 Hz, 2 H); HRMS (ESI-TOF) *m/z:* $[M + H]^+$ Calcd for $C_{25}H_{23}O_4$ 387.1596; Found 387.1594. The spectroscopic data are in accordance with literature data.[17]

Veratraldiacetophenone (4io). The standard procedure 2 was followed by use of Ca@SiO_2 (68.1 mg, 40.0 wt%, for Ca: 27.2 mg, 4.2 equiv) in 2-MeTHF (2.0 mL), 3,4-

dimethoxybenzaldehyde (**1i**, 26.8 mg, 0.161 mmol, 1.0 equiv), and acetophenone (**2o**, 42.9 mg, 0.357 mmol, 2.2 equiv) in 2-MeTHF (1.0 mL). After the reaction mixture was stirred at 25 °C for 18 h, the crude product was purified by use of column chromatography (20% EtOAc in hexanes as the eluent) to give the desired 1,5-diketone **4io** (48.6 mg, 0.125 mmol) in 78% yield as a colorless oil: TLC R_f 0.33 (50% EtOAc in hexanes as the eluent); ^{1}H NMR ($CDCl_3$, 400 MHz) δ 7.92 (d, J = 7.6 Hz, 4 H), 7.52 (t, J = 6.8 Hz, 2 H), 7.42 (t, J = 7.4 Hz, 4 H), 6.80–6.73 (m, 3 H), 4.03–3.96 (m, 1 H), 3.79 (s, 6 H), 3.45 (dd, J = 16.4 Hz, 6.8 Hz, 2 H), 3.30 (dd, J = 16.4 Hz, 7.2 Hz, 2 H); $^{13}C\{^1H\}$ NMR ($CDCl_3$, 100 MHz) δ 198.6, 148.7, 147.5, 136.8, 136.2, 132.9, 128.5, 128.0, 118.9, 111.2, 111.1, 55.7, 55.7, 45.0, 36.9; IR (neat) 3060 (w), 1682 (s, C=O), 1596 (m), 1517 (s), 1448 (m), 1260 (s), 1027 (m), 757 (m) cm^{-1}; HRMS (ESI-TOF) *m/z*: $[M + H]^+$ Calcd for $C_{25}H_{25}O_4$ 389.1752; Found 389.1748. The compound is reported in literature.[19]

3-(2-Bromo-4,5-dimethoxyphenyl)-1,5-bis(4-bromophenyl)pentane-1,5-dione (4ku). The standard procedure 2 was followed by use of Ca@SiO_2 (54.3 mg, 40.0 wt%, for Ca: 21.7 mg, 4.2 equiv) in 2-MeTHF (2.0 mL), 2-bromo-4,5-dimethoxybenzaldehyde (**1k**, 31.5 mg, 0.129 mmol, 1.0 equiv), and 4′-bromoacetophenone (**2u**, 56.5 mg, 0.284 mmol, 2.2 equiv) in 2-MeTHF (1.0 mL). After the reaction mixture was stirred at 25 °C for 18 h, the crude product was purified by use of column chromatography (25% EtOAc in hexanes as the eluent) to give the desired 1,5-diketone **4ku** (64.4 mg, 0.103 mmol) in 80% yield as pale yellow solids: mp (recrystallized from EtOH) 144.5–146.4 °C; TLC R_f 0.39 (40% EtOAc in hexanes as the eluent); ^{1}H NMR ($CDCl_3$, 400 MHz) δ 7.80 (d, J = 8.4 Hz, 4 H), 7.56 (d, J = 8.4 Hz, 4 H), 6.95 (s, 1 H), 6.76 (s, 1 H), 4.39–4.32 (m, 1 H), 3.79 (s, 3 H), 3.78 (s, 3 H), 3.44 (dd, J = 16.6 Hz, 6.8 Hz, 2 H), 3.30 (dd, J = 16.6 Hz, 6.8 Hz, 2 H); $^{13}C\{^1H\}$ NMR ($CDCl_3$, 100 MHz) δ 197.4, 148.4, 148.2, 135.3, 133.5, 131.8, 129.6, 128.3, 115.8, 113.9, 111.2, 56.1, 56.0, 43.2, 36.4; IR

(neat) 3060 (w), 1682 (s, C=O), 1584 (s), 1505 (s), 1396 (m), 1214 (s), 1070 (m), 813 (m) cm^{-1}; HRMS (ESI-TOF) *m/z:* $[M + Na]^+$ Calcd for $C_{25}H_{21}Br_3O_4Na$ 644.8887; Found 644.8880.

3-(Benzo[*d*][1,3]dioxol-5-yl)-1,5-diphenylpentane-1,5-dione (4jo). The standard procedure 2 was followed by use of Ca@SiO_2 (59.3 mg, 40.0 wt%, for Ca: 23.7 mg, 4.1 equiv) in 2-MeTHF (2.0 mL), 1,3-benzodioxole-5-carboxaldehyde (**1j**, 21.4 mg, 0.143 mmol, 1.0 equiv), and acetophenone (**2o**, 37.9 mg, 0.315 mmol, 2.2 equiv) in 2-MeTHF (1.0 mL). After the reaction mixture was stirred at 25 °C for 19 h, the crude product was purified by use of column chromatography (25% EtOAc in hexanes as the eluent) to give the desired 1,5-diketone **4jo** (44.3 mg, 0.119 mmol) in 83% yield as white solids: mp (recrystallized from EtOH) 114.4–115.8 °C; TLC R_f 0.47 (50% EtOAc in hexanes as the eluent); ^1H NMR ($CDCl_3$, 400 MHz) δ 7.93 (d, J = 7.6 Hz, 4 H), 7.52 (t, J = 7.2 Hz, 2 H), 7.42 (t, J = 7.6 Hz, 4 H), 6.76 (s, 1 H), 6.71–6.66 (m, 2 H), 5.86 (s, 2 H), 4.01–3.94 (m, 1 H), 3.42 (dd, J = 16.8 Hz, 7.2 Hz, 2 H), 3.27 (dd, J = 16.8 Hz, 7.2 Hz, 2 H); HRMS (ESI-TOF) *m/z:* $[M + H]^+$ Calcd for $C_{24}H_{21}O_4$ 373.1439; Found 373.1435. The spectroscopic data are in accordance with literature data.[18]

3-(Naphthalen-1-yl)-1,5-diphenylpentane-1,5-dione (4lo). The standard procedure 2 was followed by use of Ca@SiO_2 (88.6 mg, 40.0 wt%, for Ca: 35.4 mg, 4.1 equiv) in 2-MeTHF (2.0 mL), 1-naphthaldehyde (**1l**, 33.4 mg, 0.214 mmol, 1.0 equiv), and acetophenone (**2o**, 57.2 mg, 0.476 mmol, 2.2 equiv) in 2-MeTHF (1.0 mL). After the reaction mixture was stirred at 25 °C for 19 h, the crude product was purified by use of column chromatography (10% EtOAc in hexanes as the eluent) to give the desired 1,5-diketone **4lo** (66.3 mg, 0.175 mmol) in 82% yield as a colorless oil: TLC R_f 0.34 (20% EtOAc in hexanes as the eluent); ^1H NMR ($CDCl_3$, 400 MHz) δ 8.20 (d, J = 8.4 Hz, 1 H), 7.92 (d, J = 8.0 Hz, 4 H), 7.83 (d, J = 7.6 Hz, 1 H), 7.69 (d, J = 8.0 Hz, 1 H), 7.53–7.36 (m, 10 H), 5.04–4.97 (m, 1 H), 3.61 (dd, J = 17.2 Hz, 7.2 Hz,

2 H), 3.51 (dd, J = 17.2 Hz, 6.4 Hz, 2 H); HRMS (ESI-TOF) *m/z:* $[M + H]^+$ Calcd for $C_{27}H_{23}O_2$ 379.1698; Found 379.1685. The spectroscopic data are in accordance with literature data.[20]

3-(2-Furyl)-1,5-diphenylpentane-1,5-dione (4mo). The standard procedure 2 was followed by use of Ca@SiO_2 (69.6 mg, 40.0 wt%, for Ca: 27.8 mg, 4.2 equiv) in 2-MeTHF (2.0 mL), 2-furancarboxaldehyde (**1m**, 15.8 mg, 0.164 mmol, 1.0 equiv), and acetophenone (**2o**, 43.4 mg, 0.361 mmol, 2.2 equiv) in 2-MeTHF (0.50 mL). After the reaction mixture was stirred at 25 °C for 20 h, the crude product was purified by use of column chromatography (6.0% EtOAc in hexanes as the eluent) to give the desired 1,5-diketone **4mo** (42.1 mg, 0.132 mmol) in 80% yield as white solids: mp (recrystallized from EtOAc/hexanes) 92.6–94.1 °C; TLC R_f 0.47 (15% EtOAc in hexanes as the eluent); ^1H NMR ($CDCl_3$, 400 MHz) δ 7.95 (d, J = 7.6 Hz, 4 H), 7.53 (t, J = 7.4 Hz, 2 H), 7.43 (t, J = 7.6 Hz, 4 H), 7.25 (d, J = 1.2 Hz, 1 H), 6.21 (t, J = 2.4 Hz, 1 H), 6.03 (d, J = 3.2 Hz, 1 H), 4.21–4.14 (m, 1 H), 3.45 (dd, J = 16.8 Hz, 6.8 Hz, 2 H), 3.39 (dd, J = 16.8 Hz, 6.8 Hz, 2 H); HRMS (ESI-TOF) *m/z:* $[M + H]^+$ Calcd for $C_{21}H_{19}O_3$ 319.1334; Found 319.1340. The spectroscopic data are in accordance with literature data.[21]

1,5-Diphenyl-3-(thiophen-2-yl)pentane-1,5-dione (4no). The standard procedure 2 was followed by use of Ca@SiO_2 (82.8 mg, 40.0 wt%, for Ca: 33.1 mg, 4.1 equiv) in 2-MeTHF (1.5 mL), 2-thiophenecarboxaldehyde (**1n**, 22.3 mg, 0.199 mmol, 1.0 equiv), and acetophenone (**2o**, 53.2 mg, 0.443 mmol, 2.2 equiv) in 2-MeTHF (1.0 mL). After the reaction mixture was stirred at 25 °C for 20 h, the crude product was purified by use of column chromatography (5.0% EtOAc in hexanes as the eluent) to give the desired 1,5-diketone **4no** (44.9 mg, 0.134 mmol) in 67% yield as pale yellow solids: mp (recrystallized from EtOAc/hexanes) 87.6–89.8 °C; TLC R_f 0.32 (15% EtOAc in hexanes as the eluent); ^1H NMR ($CDCl_3$, 400 MHz) δ 7.94 (d, J = 7.6 Hz, 4 H), 7.53 (t, J = 7.4 Hz, 2 H), 7.43 (t, J = 7.6 Hz, 4 H), 7.09 (d, J = 4.8 Hz, 1

H), 6.87–6.84 (m, 2 H), 4.44–4.38 (m, 1 H), 3.51 (dd, J = 16.8 Hz, 6.4 Hz, 2 H), 3.41 (dd, J = 16.8 Hz, 6.8 Hz, 2 H); HRMS (ESI-TOF) *m/z:* $[M + Na]^+$ Calcd for $C_{21}H_{18}O_2SNa$ 357.0925; Found 357.0919. The spectroscopic data are in accordance with literature data.[16]

1,5-Bis(4-methylphenyl)-3-phenylpentane-1,5-dione (4ap). The standard procedure 2 was followed by use of Ca@SiO_2 (77.8 mg, 40.0 wt%, for Ca: 31.1 mg, 4.2 equiv) in 2-MeTHF (1.5 mL), benzaldehyde (**1a**, 19.4 mg, 0.183 mmol, 1.0 equiv), and 4′-methylacetophenone (**2p**, 55.2 mg, 0.411 mmol, 2.2 equiv) in 2-MeTHF (1.0 mL). After the reaction mixture was stirred at 25 °C for 19 h, the crude product was purified by use of column chromatography (10% EtOAc in hexanes as the eluent) to give the desired 1,5-diketone **4ap** (55.3 mg, 0.155 mmol) in 85% yield as white solids: mp (recrystallized from EtOAc/hexanes) 104.2–106.6 °C; TLC R_f 0.40 (15% EtOAc in hexanes); ^1H NMR ($CDCl_3$, 400 MHz) δ 7.83 (d, J = 8.0 Hz, 4 H), 7.27–7.13 (m, 9 H), 4.08–4.01 (m, 1 H), 3.44 (dd, J = 16.4 Hz, 6.8 Hz, 2 H), 3.30 (dd, J = 16.4 Hz, 6.8 Hz, 2 H), 2.37 (s, 6 H); HRMS (ESI-TOF) *m/z:* $[M + H]^+$ Calcd for $C_{25}H_{25}O_2$ 357.1854; Found 357.1852. The spectroscopic data are in accordance with literature data.[16]

1,5-Bis(4-methoxyphenyl)-3-phenylpentane-1,5-dione (4ar). The standard procedure 2 was followed by use of Ca@SiO_2 (91.3 mg, 40.0 wt%, for Ca: 36.5 mg, 4.2 equiv) in 2-MeTHF (1.5 mL), benzaldehyde (**1a**, 22.8 mg, 0.215 mmol, 1.0 equiv), and 4′-methoxyacetophenone (**2r**, 71.1 mg, 0.473 mmol, 2.2 equiv) in 2-MeTHF (0.50 mL). After the reaction mixture was stirred at 25 °C for 20 h, the crude product was purified by use of column chromatography (15% EtOAc in hexanes as the eluent) to give the desired 1,5-diketone **4ar** (65.3 mg, 0.168 mmol) in 78% yield as a colorless oil: TLC R_f 0.31 (40% EtOAc in hexanes as the eluent); ^1H NMR ($CDCl_3$, 400 MHz) δ 7.91 (d, J = 8.8 Hz, 4 H), 7.24–7.13 (m, 5 H), 6.88 (d, J = 8.8 Hz, 4 H), 4.04–3.97 (m, 1 H), 3.83 (s, 6 H), 3.40 (dd, J = 16.4 Hz, 7.2 Hz, 2 H), 3.25 (dd, J = 16.4

Hz, 7.2 Hz, 2 H); HRMS (ESI-TOF) *m/z:* $[M + H]^+$ Calcd for $C_{25}H_{25}O_4$ 389.1752; Found 389.1753. The spectroscopic data are in accordance with literature data.[16]

1,5-Bis(4-bromophenyl)-3-phenylpentane-1,5-dione (4au). The standard procedure 2 was followed by use of Ca@SiO_2 (81.6 mg, 40.0 wt%, for Ca: 32.6 mg, 4.1 equiv) in 2-MeTHF (1.5 mL), benzaldehyde (**1a**, 20.9 mg, 0.197 mmol, 1.0 equiv), and 4′-bromoacetophenone (**2u**, 86.6 mg, 0.435 mmol, 2.2 equiv) in 2-MeTHF (1.0 mL). After the reaction mixture was stirred at 25 °C for 18 h, the crude product was purified by use of column chromatography (7.0% EtOAc in hexanes as the eluent) to give the desired 1,5-diketone **4au** (77.4 mg, 0.159 mmol) in 81% yield as white soilds: mp (recrystallized from EtOAc/hexanes) 128.2–129.6 °C; TLC R_f 0.32 (15% EtOAc in hexanes as the eluent); ^{1}H NMR ($CDCl_3$, 400 MHz) δ 7.78 (d, J = 8.8 Hz, 4 H), 7.56 (d, J = 8.8 Hz, 4 H), 7.28–7.15 (m, 5 H), 4.03–3.96 (m, 1 H), 3.43 (dd, J = 16.4 Hz, 6.8 Hz, 2 H), 3.28 (dd, J = 16.8 Hz, 6.8 Hz, 2 H); HRMS (ESI-TOF) *m/z:* $[M + H]^+$ Calcd for $C_{23}H_{19}Br_2O_2$ 484.9751; Found 484.9759. The spectroscopic data are in accordance with literature data.[22]

1,5-Bis(4-cyanophenyl)-3-phenylpentane-1,5-dione (4av). The standard procedure 2 was followed by use of Ca@SiO_2 (69.1 mg, 40.0 wt%, for Ca: 27.6 mg, 4.2 equiv) in 2-MeTHF (1.5 mL), benzaldehyde (**1a**, 17.3 mg, 0.163 mmol, 1.0 equiv), and 4-acetylbenzonitrile (**2v**, 52.6 mg, 0.362 mmol, 2.2 equiv) in 2-MeTHF (1.0 mL). After the reaction mixture was stirred at 25 °C for 18 h, the crude product was purified by use of column chromatography (20% EtOAc in hexanes as the eluent) to give the desired 1,5-diketone **4av** (47.1 mg, 0.124 mmol) in 76% yield as a colorless oil: TLC Rf 0.36 (25% EtOAc in hexanes as the eluent); ^{1}H NMR ($CDCl_3$, 400 MHz) δ 7.99 (d, J = 8.4 Hz, 4 H), 7.73 (d, J = 8.0 Hz, 4 H), 7.29–7.18 (m, 5 H), 4.03–3.96 (m, 1 H), 3.49 (dd, J = 17.2 Hz, 7.2 Hz, 2 H), 3.34 (dd, J = 17.2 Hz, 6.8 Hz, 2 H);

$^{13}C\{^{1}H\}$ NMR ($CDCl_3$, 100 MHz) δ 197.1, 142.7, 139.6, 132.5, 128.8, 128.5, 127.2, 127.1, 117.8, 116.5, 44.9, 36.9; IR (neat) 2924 (w), 2230 (m, C≡N), 1690 (s, C=O), 1404 (m), 1211 (w), 992 (w), 831 (w), 701 (m) cm^{-1}; HRMS (ESI-TOF) *m/z:* $[M + Na]^+$ Calcd for $C_{25}H_{18}N_2O_2Na$ 401.1266; Found 401.1269.

1,5-Bis(3-methoxyphenyl)-3-phenylpentane-1,5-dione (4aq). The standard procedure 2 was followed by use of Ca@SiO_2 (63.1 mg, 40.0 wt%, for Ca: 25.2 mg, 4.2 equiv) in 2-MeTHF (1.5 mL), benzaldehyde (**1a**, 15.8 mg, 0.149 mmol, 1.0 equiv), and 3′-methoxyacetophenone (**2q**, 49.7 mg, 0.331 mmol, 2.2 equiv) in 2-MeTHF (1.0 mL). After the reaction mixture was stirred at 25 °C for 19 h, the crude product was purified by use of column chromatography (18% EtOAc in hexanes as the eluent) to give the desired 1,5-diketone **4aq** (46.3 mg, 0.119 mmol) in 80% yield as a colorless oil: TLC R_f 0.22 (20% EtOAc in hexanes as the eluent); ^{1}H NMR ($CDCl_3$, 400 MHz) δ 7.52 (d, J = 7.6 Hz, 2 H), 7.44 (s, 2 H), 7.33 (t, J = 7.8 Hz, 2 H), 7.26 (d, J = 4.0 Hz, 4 H), 7.19–7.14 (m, 1 H), 7.08–7.06 (m, 2 H), 4.08–4.01 (m, 1 H), 3.82 (s, 6 H), 3.45 (dd, J = 16.8 Hz, 7.2 Hz, 2 H), 3.31 (dd, J = 16.8 Hz, 7.2 Hz, 2 H); $^{13}C\{^{1}H\}$ NMR ($CDCl_3$, 100 MHz) δ 198.0, 159.5, 143.5, 138.0, 129.4, 128.4, 127.3, 126.5, 120.6, 119.5, 112.1, 55.4, 45.0, 37.3; IR (neat) 3062 (w), 1684 (s, C=O), 1596 (m), 1430 (m), 1258 (s), 1040 (m), 873 (w), 700 (m) cm^{-1}; HRMS (ESI-TOF) *m/z:* $[M + H]^+$ Calcd for $C_{25}H_{25}O_4$ 389.1752; Found 389.1751.

1,5-Bis(3-Bromophenyl)-3-phenylpentane-1,5-dione (4at). The standard procedure 2 was followed by use of Ca@SiO_2 (81.3 mg, 40.0 wt%, for Ca: 32.5 mg, 4.1 equiv) in 2-MeTHF (1.5 mL), benzaldehyde (**1a**, 20.8 mg, 0.196 mmol, 1.0 equiv), and 3′-bromoacetophenone (**2t**, 86.1 mg, 0.433 mmol, 2.2 equiv) in 2-MeTHF (0.50 mL). After the reaction mixture was stirred at 25 °C for 20 h, the crude product was purified by use of column chromatography (10%

EtOAc in hexanes as the eluent) to give the desired 1,5-diketone **4at** (74.4 mg, 0.153 mmol) in 78% yield as a colorless oil: TLC R_f 0.26 (15% EtOAc in hexanes as the eluent); ^{1}H NMR ($CDCl_3$, 400 MHz) δ 8.02 (s, 2 H), 7.84 (d, J = 8.0 Hz, 2 H), 7.65 (d, J = 8.0 Hz, 2 H), 7.32–7.25 (m, 6 H), 7.19–7.16 (m, 1 H), 4.04–3.97 (m, 1 H), 3.43 (dd, J = 16.8 Hz, 6.8 Hz, 2 H), 3.30 (dd, J = 16.8 Hz, 6.8 Hz, 2 H); ^{13}C{^{1}H} NMR ($CDCl_3$, 100 MHz) δ 196.7, 143.1, 138.3, 135.8, 131.0, 130.0, 128.6, 127.2, 126.7, 126.5, 122.8, 44.8, 36.9; IR (neat) 3062 (w), 1686 (s, C=O), 1566 (m), 1419 (m), 1204 (m), 995 (w), 786 (m), 701 (m) cm^{-1}; HRMS (ESI-TOF) *m/z:* $[M + H]^+$ Calcd for $C_{23}H_{19}Br_2O_2$ 484.9751; Found 484.9756.

3-Phenyl-1,5-bis(2-pyridyl)pentane-1,5-dione (4aw). The standard procedure 2 was followed by use of Ca@SiO_2 (75.6 mg, 40.0 wt%, for Ca: 30.2 mg, 4.2 equiv) in 2-MeTHF (1.0 mL), benzaldehyde (**1a**, 18.9 mg, 0.178 mmol, 1.0 equiv), and 2-acetylpyridine (**2w**, 47.5 mg, 0.392 mmol, 2.2 equiv) in 2-MeTHF (1.0 mL). After the reaction mixture was stirred at 25 °C for 18 h, the crude product was purified by use of column chromatography (25% EtOAc in hexanes as the eluent) to give the desired 1,5-diketone **4aw** (44.3 mg, 0.134 mmol) in 75% yield as white solids: mp (recrystallized from EtOH) 148.6–149.9 °C; TLC R_f 0.28 (25% EtOAc in hexanes as the eluent); ^{1}H NMR ($CDCl_3$, 400 MHz) δ 8.61 (d, J = 4.4 Hz, 2 H), 7.92 (d, J = 8.0 Hz, 2 H), 7.75 (t, J = 7.2 Hz, 2 H), 7.40 (t, J = 6.0 Hz, 2 H), 7.35 (d, J = 7.6 Hz, 2 H), 7.21 (t, J = 8.0 Hz, 2 H), 7.10 (t, J = 7.4 Hz, 1 H), 4.17–4.10 (m, 1 H), 3.74 (dd, J = 17.6 Hz, 7.6 Hz, 2 H,), 3.61 (dd, J = 17.6 Hz, 6.8 Hz, 2); HRMS (ESI-TOF) *m/z:* $[M + H]^+$ Calcd for $C_{21}H_{19}N_2O_2$ 331.1446; Found 331.1448. The spectroscopic data are in accordance with literature data.[23]

Calculation of Atom Economy and Atom Efficiency

Table S3. Calculation of Atom Economy and Atom Efficiency for the Enones 3

compound	mol. wt.	yield (%)	atom economy (%)	atom efficiency (%)
**3ao**	208.26	85	92.0	78.2
**3ho**	266.30	76	93.7	71.2
**3ap**	222.29	86	92.5	79.6
**3aq**	238.29	71	92.9	66.0
**3as**	287.16	90	94.1	84.7
**3bs**	301.18	87	94.4	82.1
**3cs**	317.18	88	94.6	83.3
**3io**	268.31	79	93.7	74.0
**3ku**	426.10	71	95.9	68.1
**3jo**	252.27	85	93.3	79.3
**3lo**	258.32	79	93.5	73.9
**3mo**	198.22	81	91.7	74.3
**3no**	214.28	76	92.2	70.1
**3aw**	209.25	75	92.1	69.1

Table S4. Calculation of Atom Economy and Atom Efficiency for the Diketones 4

compound	mol. wt.	yield (%)	atom economy (%)	atom efficiency (%)
**4ao**	328.41	82	94.8	77.7
**4bo**	342.44	79	95.0	75.1
**4co**	358.44	77	95.2	73.3
**4do**	346.40	88	95.1	83.7
**4eo**	362.85	73	95.3	69.5
**4fo**	407.31	85	95.8	81.4
**4go**	373.41	73	95.4	69.6
**4ho**	386.45	76	95.5	72.6
**4io**	388.46	78	95.6	74.5
**4ku**	625.15	80	97.2	77.8
**4jo**	372.42	83	95.4	79.2
**4lo**	378.47	82	95.5	78.3
**4mo**	318.37	80	94.6	75.7
**4no**	334.43	67	94.9	59.8
**4ap**	356.47	85	95.2	80.9
**4ar**	388.46	78	95.6	74.5
**4au**	486.20	81	96.4	78.1
**4av**	378.43	76	95.5	72.5
**4aq**	388.46	80	95.6	76.5
**4at**	486.20	78	96.4	75.2
**4aw**	330.39	75	94.8	71.1

References

(1) Caschili, S.; Delogu, F.; Concas, A.; Pisu, M.; Cao, G. Mechanically induced self-propagating reactions: Analysis of reactive substrates and degradation of aromatic sulfonic pollutants. *Chemosphere* **2006**, *63*, 987–995.

(2) Deidda, C.; Doppiu, S.; Monagheddu, M.; Cocco, G. A direct view of the self combustion behaviour of TiC system under milling. *J. Metast. Nanocryst. Mater.* **2003**, *15–16*, 215–220.

(3) Cao, G.; Doppiu, S.; Monagheddu, M.; Orrù, R.; Sannia, M.; Cocco, G. Thermal and mechanochemical self-propagating degradation of chloro-organic compounds: the case of hexachlorobenzene over calcium hydride. *Ind. Eng. Chem. Res.* **1999**, *38*, 3218–3224.

(4) Li, L.; Stimac, J. C.; Geary, L. M. Synthesis of olefins via a Wittig reaction mediated by triphenylarsine. *Tetrahedron Lett.* **2017**, *58*, 1379–1381.

(5) Kazi, I.; Guha, S.; Sekar, G. CBr_4 as a halogen bond donor catalyst for the selective activation of benzaldehydes to synthesize α,β-unsaturated ketones. *Org. Lett.* **2017**, *19*, 1244–1247.

(6) Mameda, N.; Peraka, S.; Kodumuri, S.; Chevella, D.; Banothu, R.; Amrutham, V.; Nama, N. Synthesis of α,β-unsaturated ketones from alkynes and aldehydes over Hβ zeolite under solvent-free conditions. *RSC Adv.* **2016**, *6*, 58137–58141.

(7) Wu, Y.; Zhou, G.; Meng, Q.; Tang, X.; Liu, G.; Yin, H.; Zhao, J.; Yang, F.; Yu, Z.; Luo, Y. Visible light-induced aerobic epoxidation of α,β-unsaturated ketones mediated by amidines. *J. Org. Chem.* **2018**, *83*, 13051–13062.

(8) Jiang, Q.; Jia, J.; Xu, B.; Zhao, A.; Guo, C.-C. Iron-facilitated oxidative radical decarboxylative cross-coupling between α-oxocarboxylic acids and acrylic acids: an approach to α,β-unsaturated carbonyls. *J. Org. Chem.* **2015**, *80*, 3586–3596.

(9) Zhang, M.; Xi, J.; Ruzi, R.; Li, N.; Wu, Z.; Li, W.; Zhu, C. Domino-fluorination–protodefluorination enables decarboxylative cross-coupling of α-oxocarboxylic acids with styrene via photoredox catalysis. *J. Org. Chem.* **2017**, *82*, 9305–9311.

(10) Fei, X.-D.; Zhou, Z.; Li, W.; Zhu, Y.-M.; Shen, J.-K. Buchwald–hartwig coupling/michael addition reactions: one-pot synthesis of 1,2-disubstituted 4-quinolones from chalcones and primary amines. *Eur. J. Org. Chem.* **2012**, 3001–3008.

(11) Thomson, C. J.; Barber, D. M.; Dixon, D. J. One-pot catalytic enantioselective synthesis of 2-pyrazolines. *Angew. Chem., Int. Ed.* **2019**, *58*, 2469–2473.

(12) Lee, D.; Kim, K. H.; Moon, S. W.; Lee, H.; Kang, K. S.; Lee, J. W. Synthesis and biological evaluation of chalcone analogues as protective agents against cisplatin-induced cytotoxicity in kidney cells. *Bioorg. Med. Chem. Lett.* **2015**, 25, 1929–1932.

(13) Chan, C.-K.; Tsai, Y.-L.; Chang, M.-Y. $Bi(OTf)_3$ catalyzed disproportionation reaction of cinnamyl alcohols. *Tetrahedron* **2017**, *73*, 3368–3376.

(14) Larionov, V. A.; Markelova, E. P.; Smol'yakov, A. F.; Savel'yeva, T. F.; Maleev, V. I.; Belokon, Y. N. Chiral octahedral complexes of Co(iii) as catalysts for asymmetric epoxidation of chalcones under phase transfer conditions. *RSC Adv.* **2015**, *5*, 72764–72771.

(15) Charvieux, A.; Giorgi, J. B.; Duguet, N.; Métay, E. Solvent-free direct α-alkylation of ketones by alcohols catalyzed by nickel supported on silica–alumina. *Green Chem.* **2018**, *20*, 4210–4216.

(16) Li, Z.; Wen, G.; He, L.; Li, J.; Jia, X.; Yang, J. Copper-catalyzed synthesis of 1,3,5-triarylpentane-1,5-diones from α,β-unsaturated ketones. *RSC Adv.* **2015**, *5*, 52121–52125.

(17) Liu, B.; Wang, J.; Pang, Y.; Ge, Z.; Li, R. Unexpected synthesis of 1,3,5-triarly-1,5-diketones from aryl ketones via di-enamine mechanism. *Tetrahedron* **2014**, *70*, 9240–9244.

(18) Koppolu, S. R.; Balamurugan, R. In situ formed acetals facilitated direct Michael addition of unactivated ketones. *New J. Chem.* **2017**, *41*, 1186–1192.

(19) Frank, R. L.; Seven, R. P. Pyridines. IV. a study of the chichibabin synthesis. *J. Am. Chem. Soc.* **1949**, *71*, 2629–2635.

(20) Yanagisawa, A.; Takahashi, H.; Arai, T. One-pot synthesis of 1,5-diketones catalyzed by barium isopropoxide. *Tetrahedron* **2007**, *63*, 8581–8585.

(21) Liu, H.; Liu, X.; Liu, L.; Zhang, X.; Li, C. Practical aqueous reactions leading to skeletally diverse carbohydrate-derived ketones. *RSC Adv.* **2015**, *5*, 11831–11836.

(22) Sodhi, R. K.; Paul, S.; Gupta, V. K.; Kant, R. Conversion of α,β-unsaturated ketones to 1,5-diones via tandem retro-Aldol and Michael addition using $Co(acac)_2$ covalently anchored onto amine functionalized silica. *Tetrahedron Lett.* **2015**, *56*, 1944–1948.

(23) Mondal, P. C.; Manna, A. K. Synthesis of heteroleptic terpyridyl complexes of Fe(ii) and Ru(ii): optical and electrochemical studies. *New J. Chem.* **2016**, *40*, 5775–5781.

Spectra of Compounds

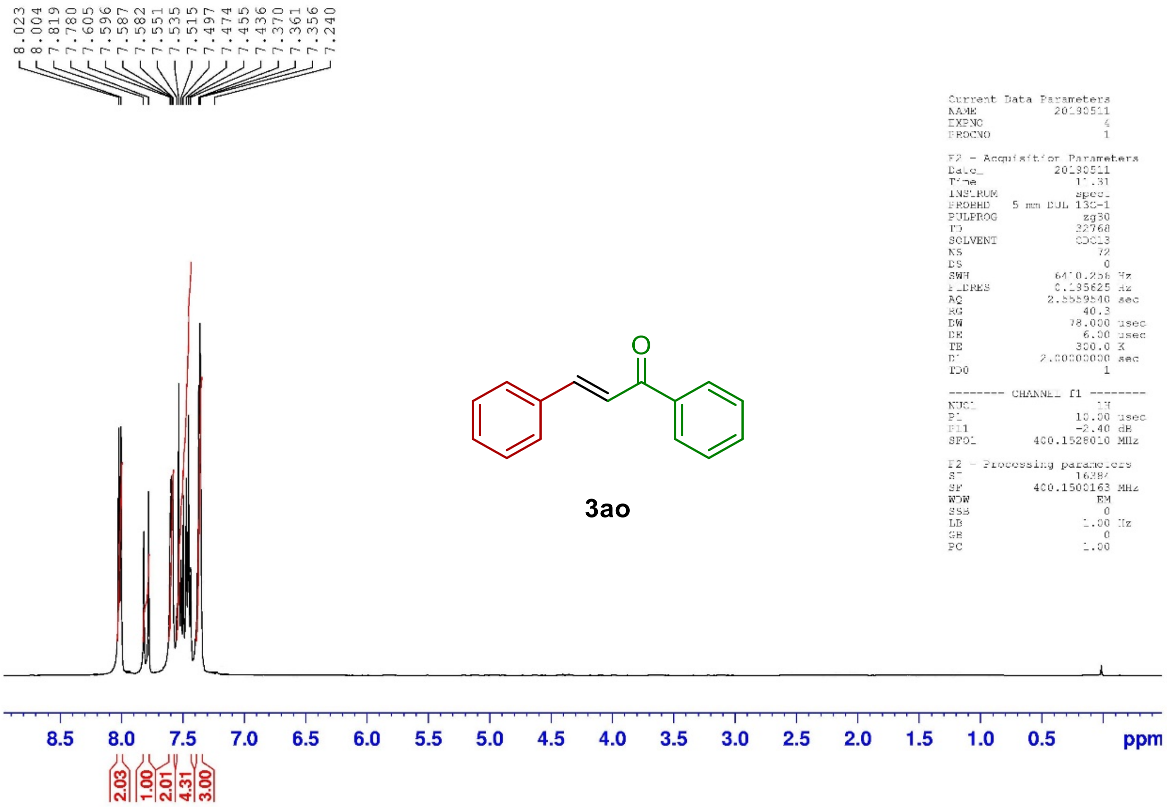

^{1}H NMR (400 MHz, $CDCl_3$) spectrum of compound **3ao**

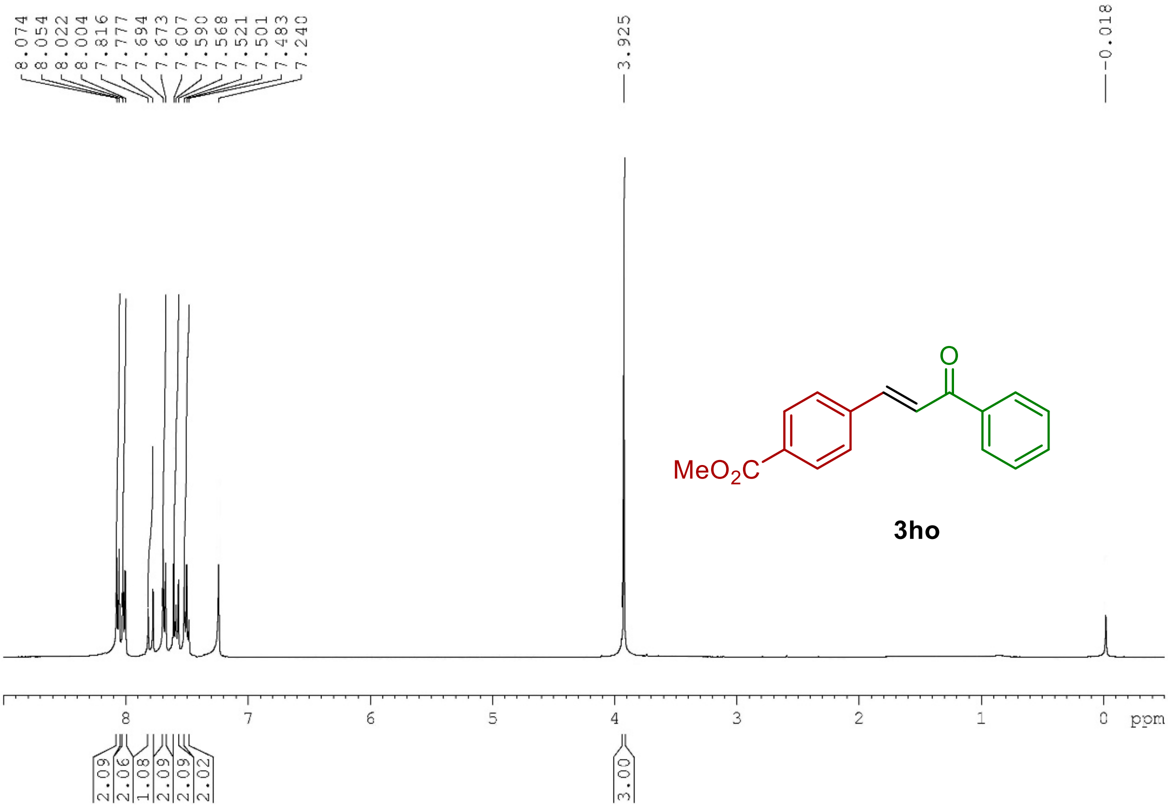

^{1}H NMR (400 MHz, $CDCl_3$) spectrum of compound **3ho**

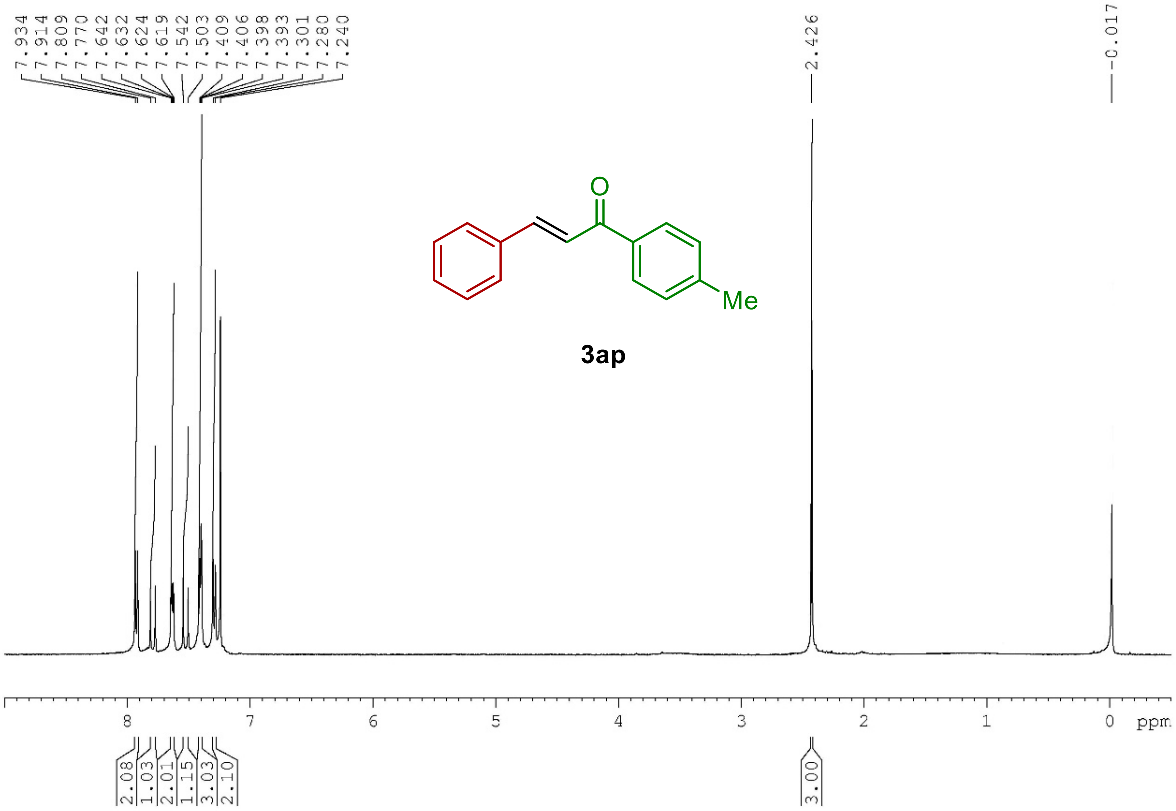

^{1}H NMR (400 MHz, $CDCl_3$) spectrum of compound **3ap**

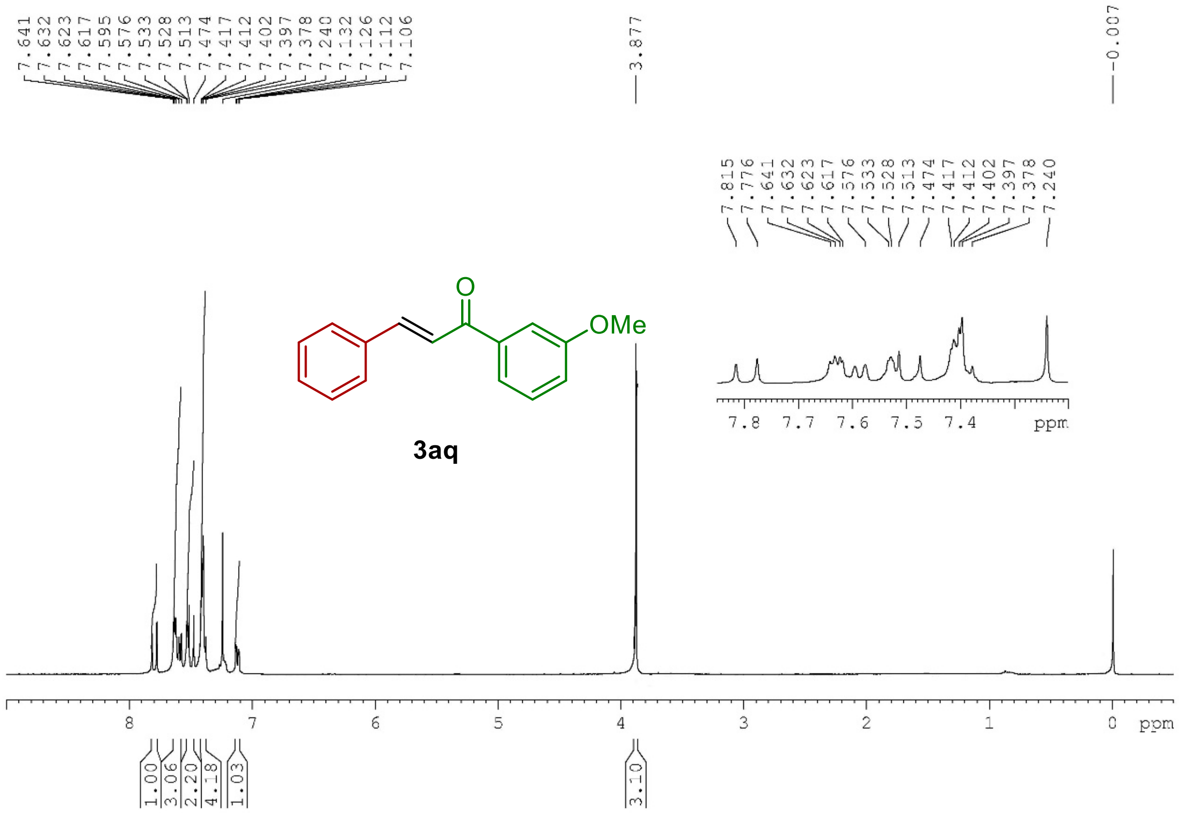

^{1}H NMR (400 MHz, $CDCl_3$) spectrum of compound **3aq**

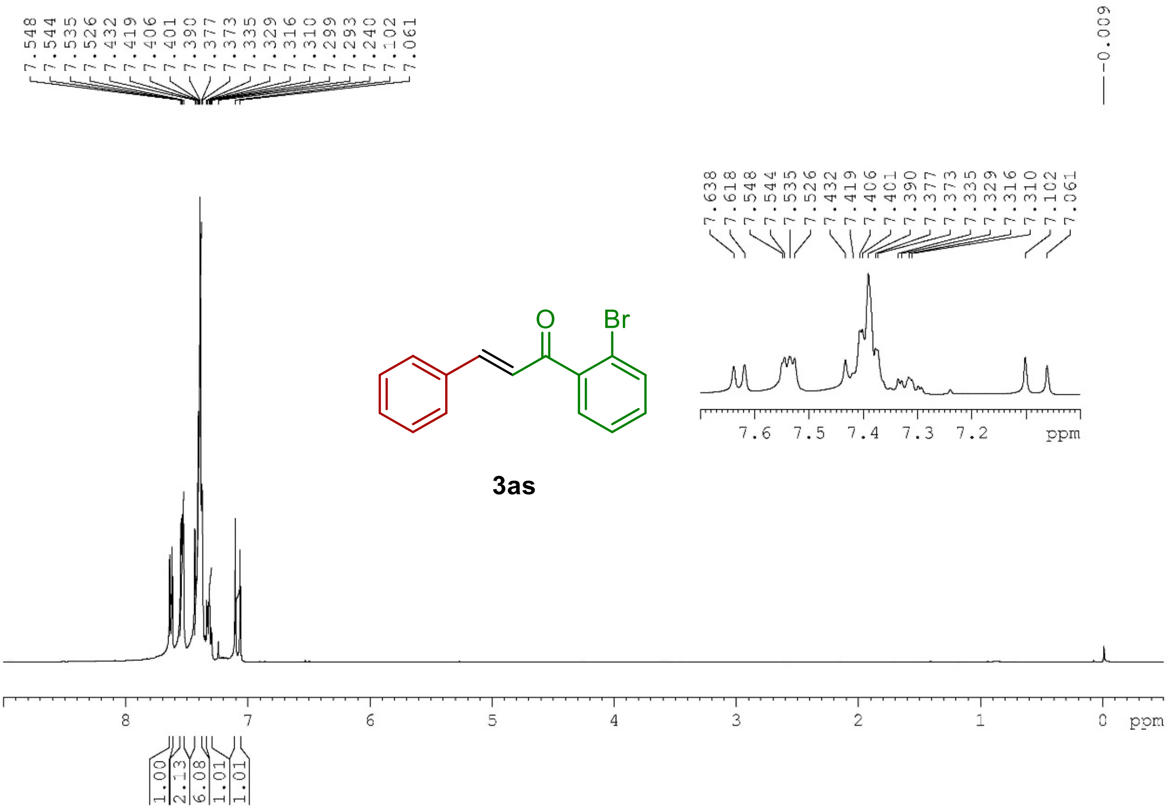

^{1}H NMR (400 MHz, $CDCl_3$) spectrum of compound **3as**

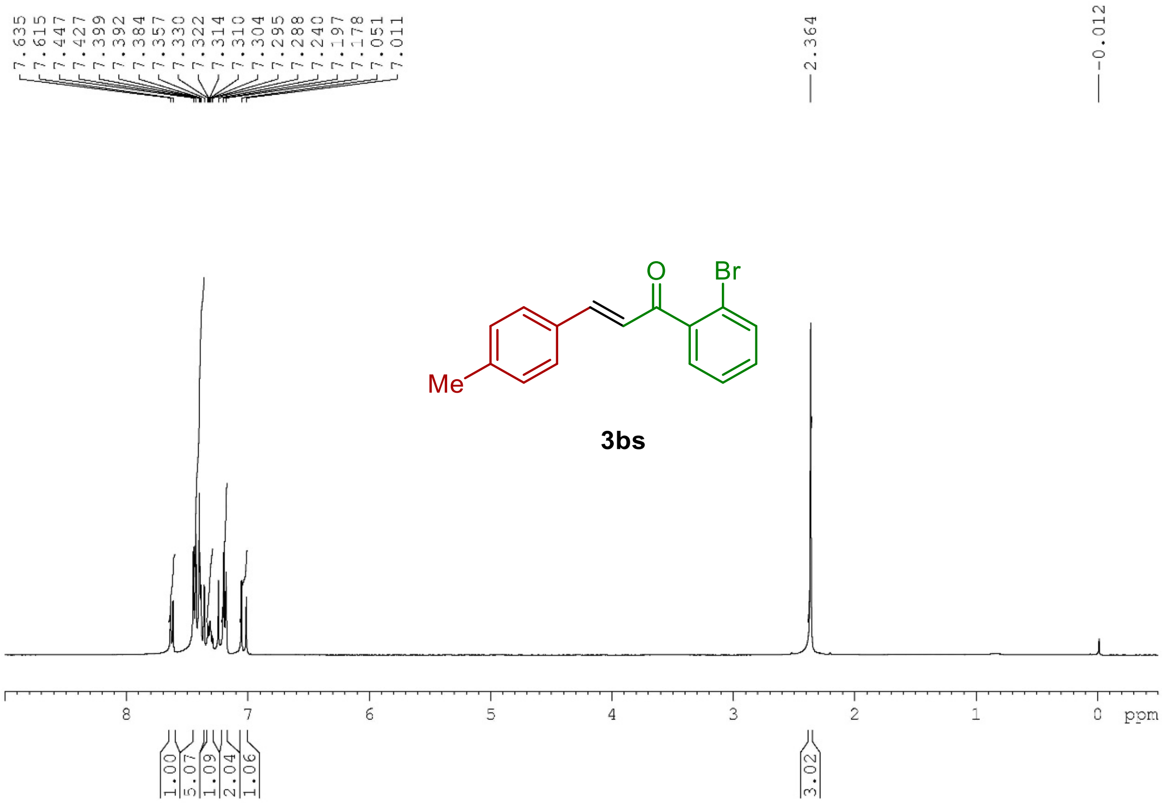

^{1}H NMR (400 MHz, $CDCl_3$) spectrum of compound **3bs**

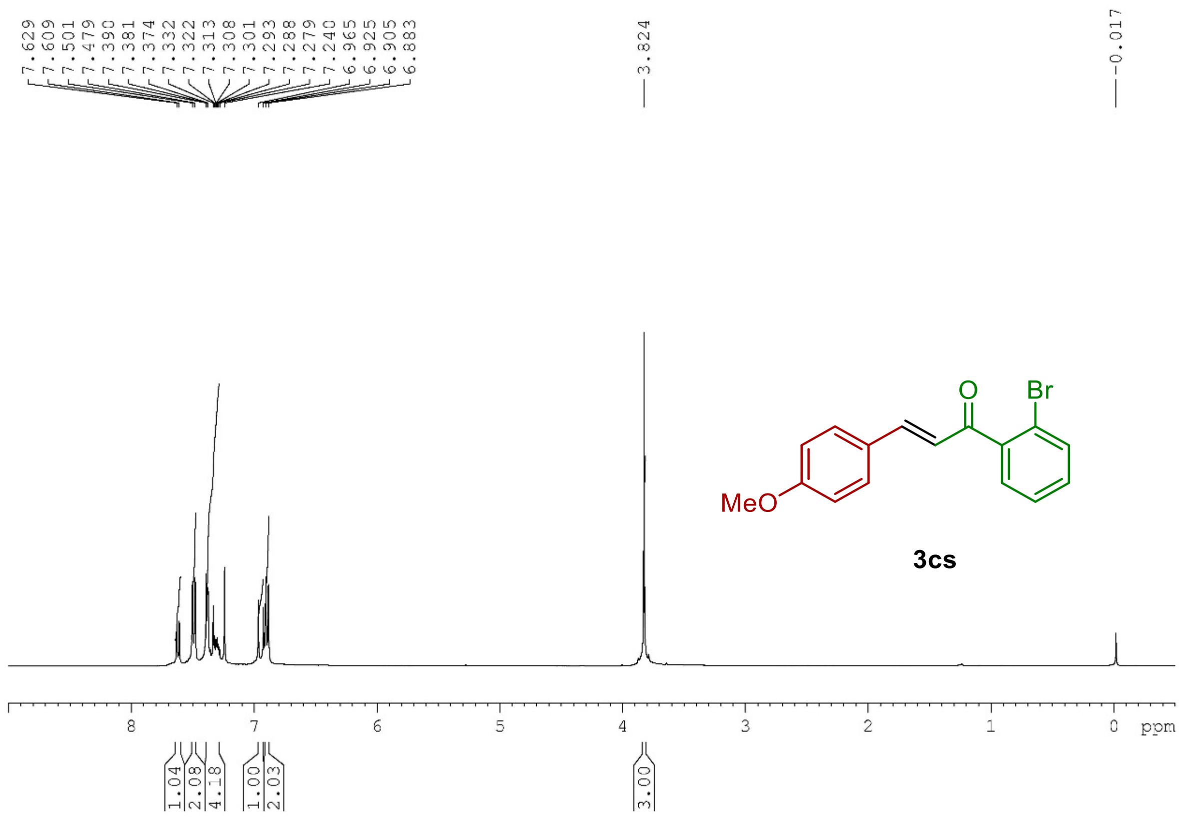

^{1}H NMR (400 MHz, $CDCl_3$) spectrum of compound **3cs**

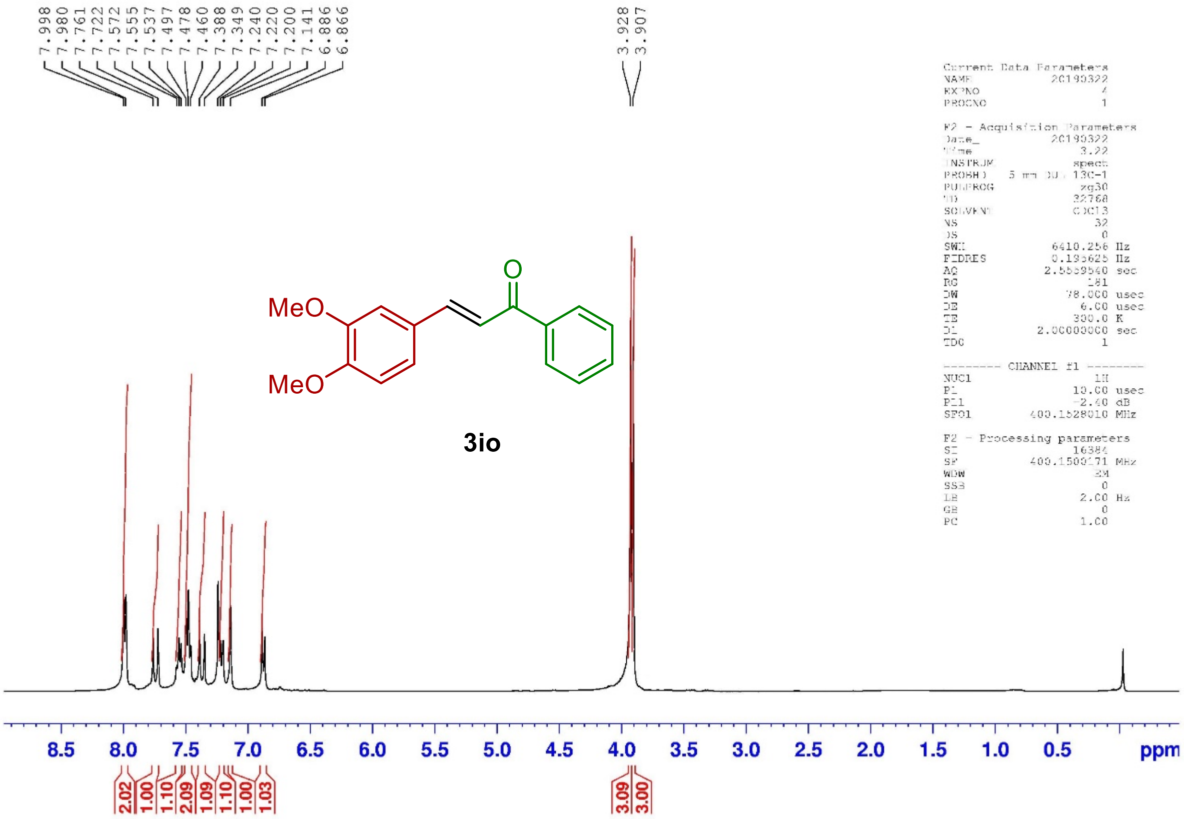

^{1}H NMR (400 MHz, $CDCl_3$) spectrum of compound **3io**

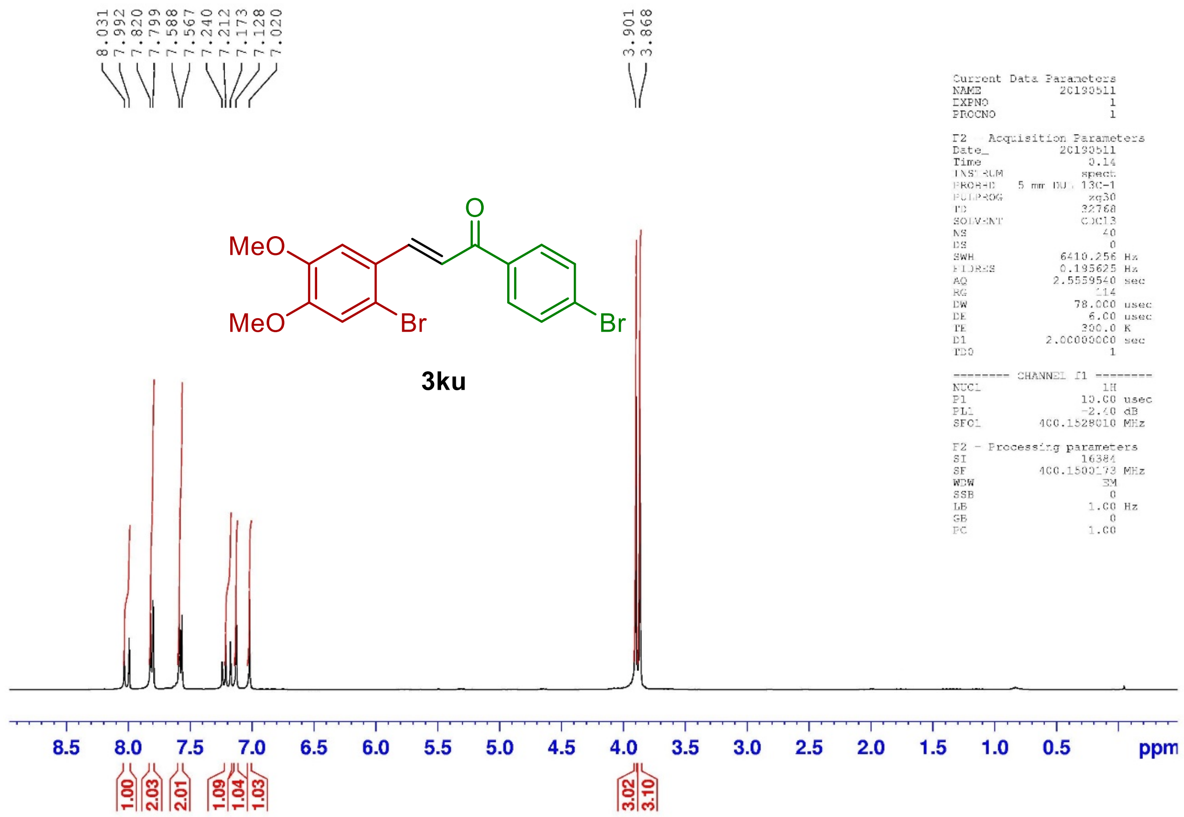

^{1}H NMR (400 MHz, $CDCl_3$) spectrum of compound **3ku**

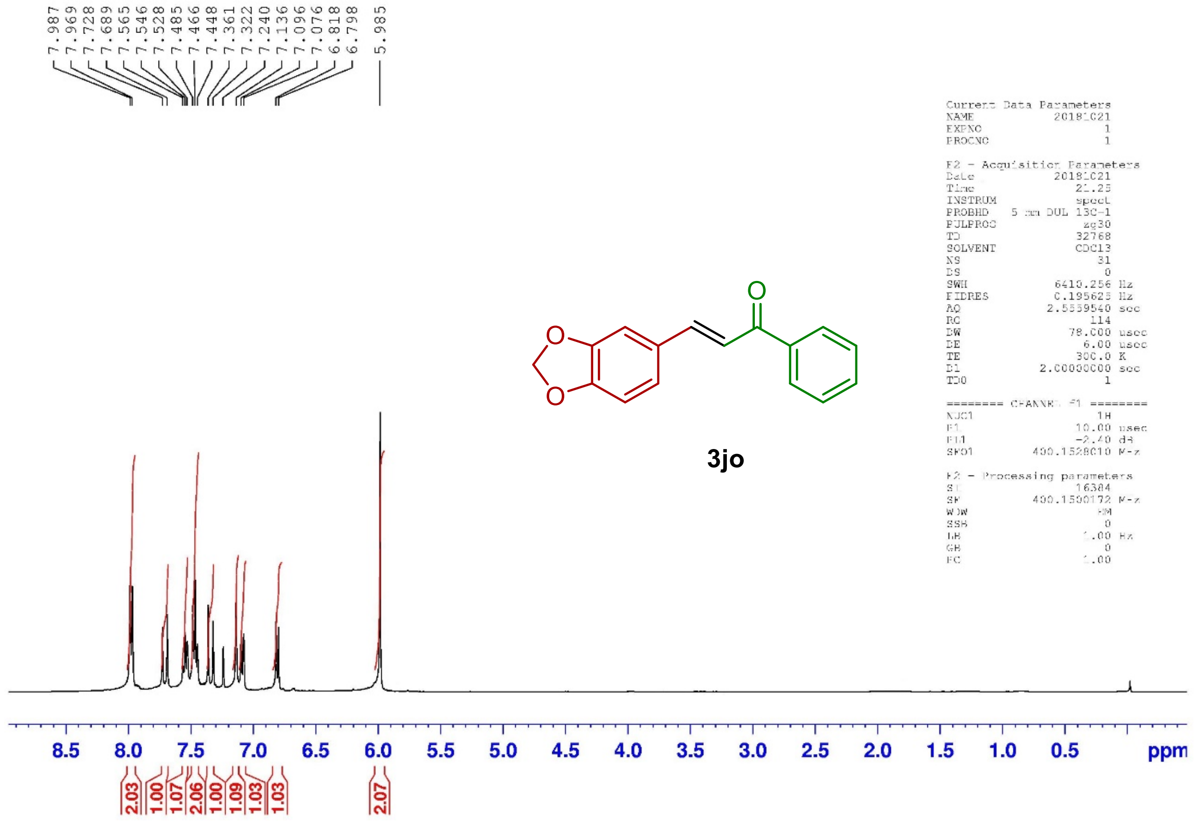

^{1}H NMR (400 MHz, $CDCl_3$) spectrum of compound **3jo**

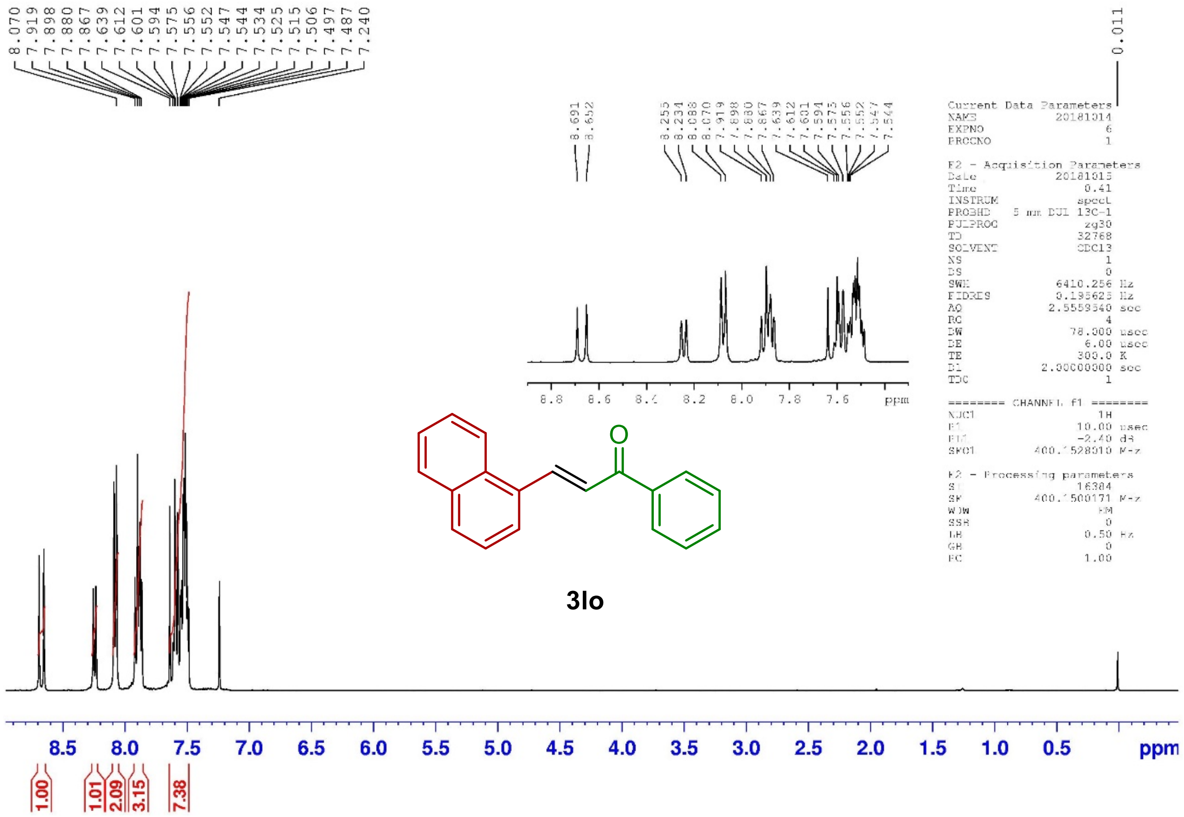

^{1}H NMR (400 MHz, $CDCl_3$) spectrum of compound **3lo**

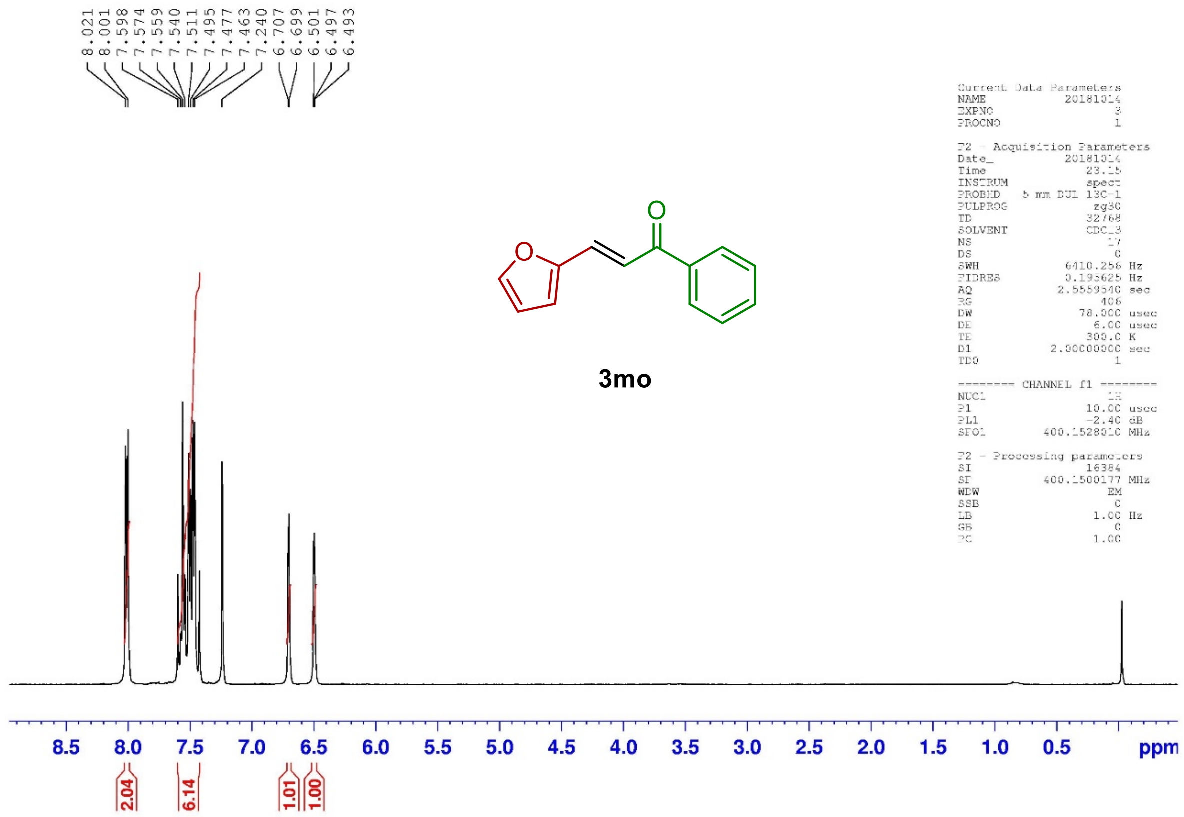

^{1}H NMR (400 MHz, $CDCl_3$) spectrum of compound **3mo**

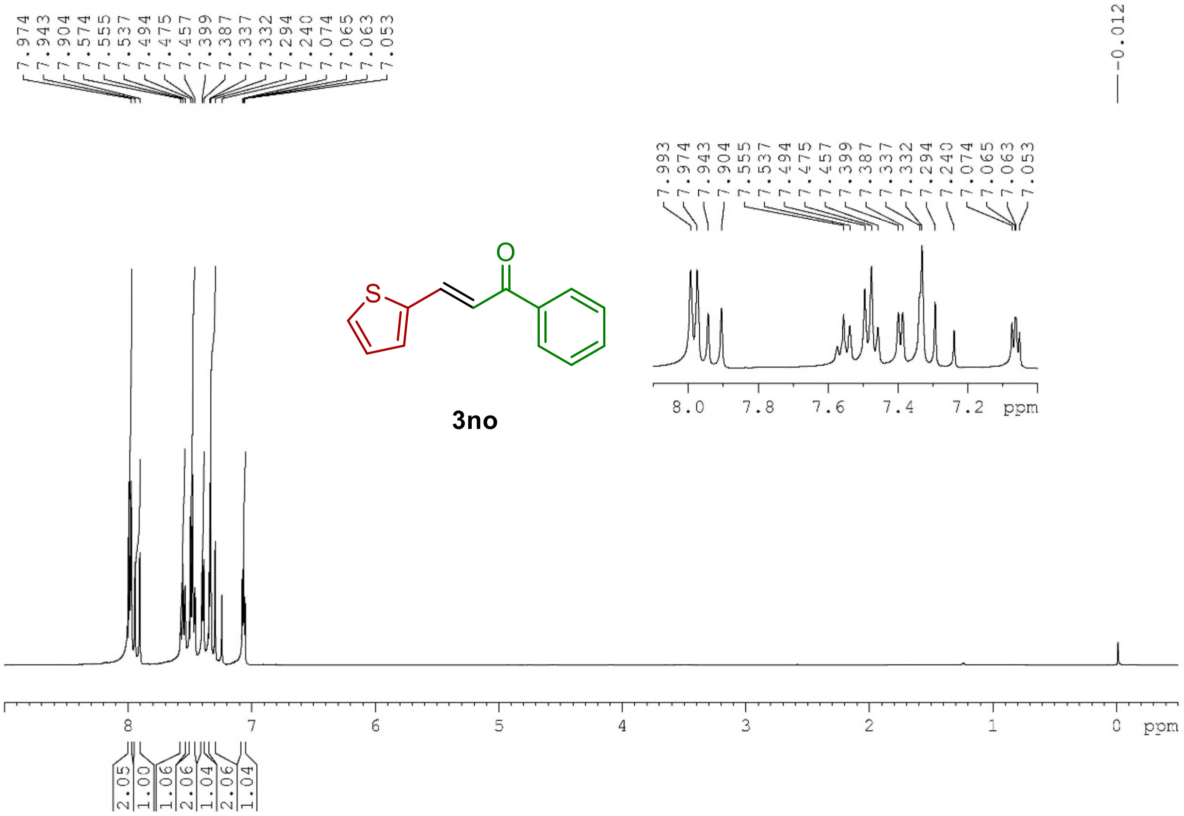

^{1}H NMR (400 MHz, $CDCl_3$) spectrum of compound **3no**

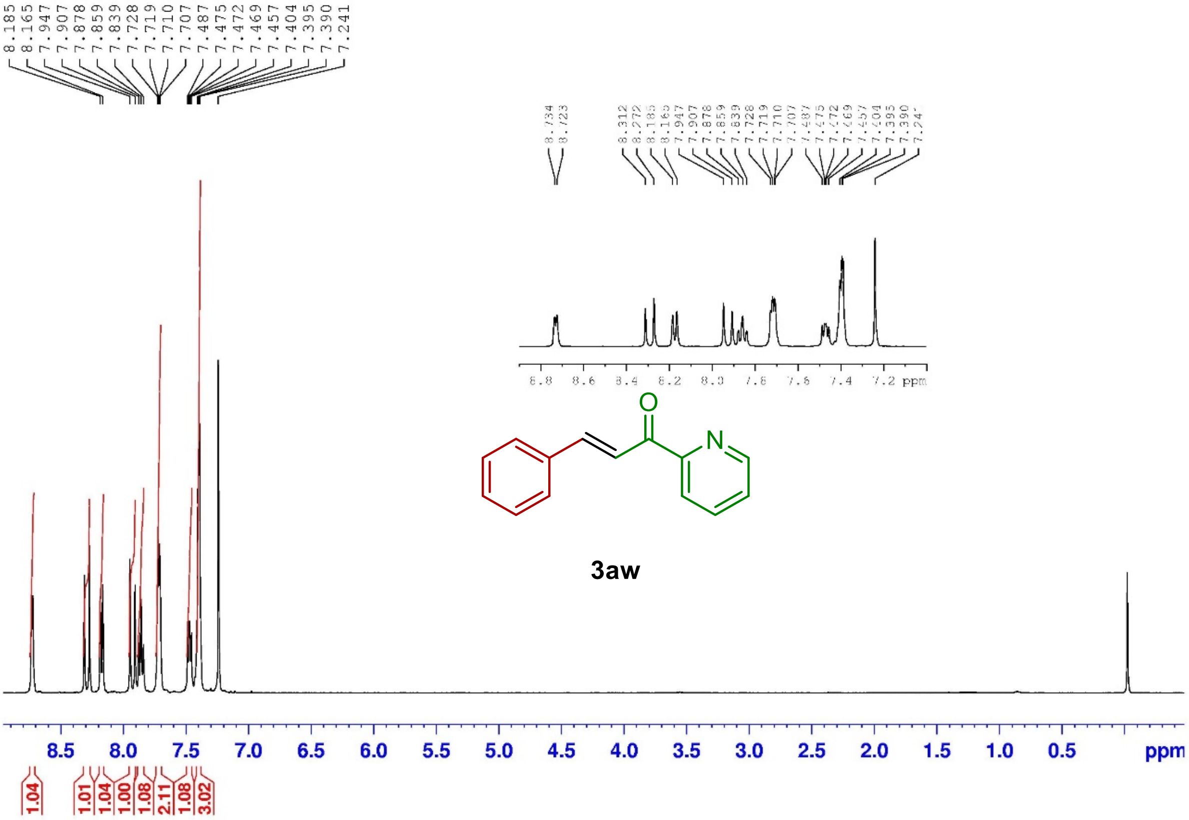

^{1}H NMR (400 MHz, $CDCl_3$) spectrum of compound **3aw**

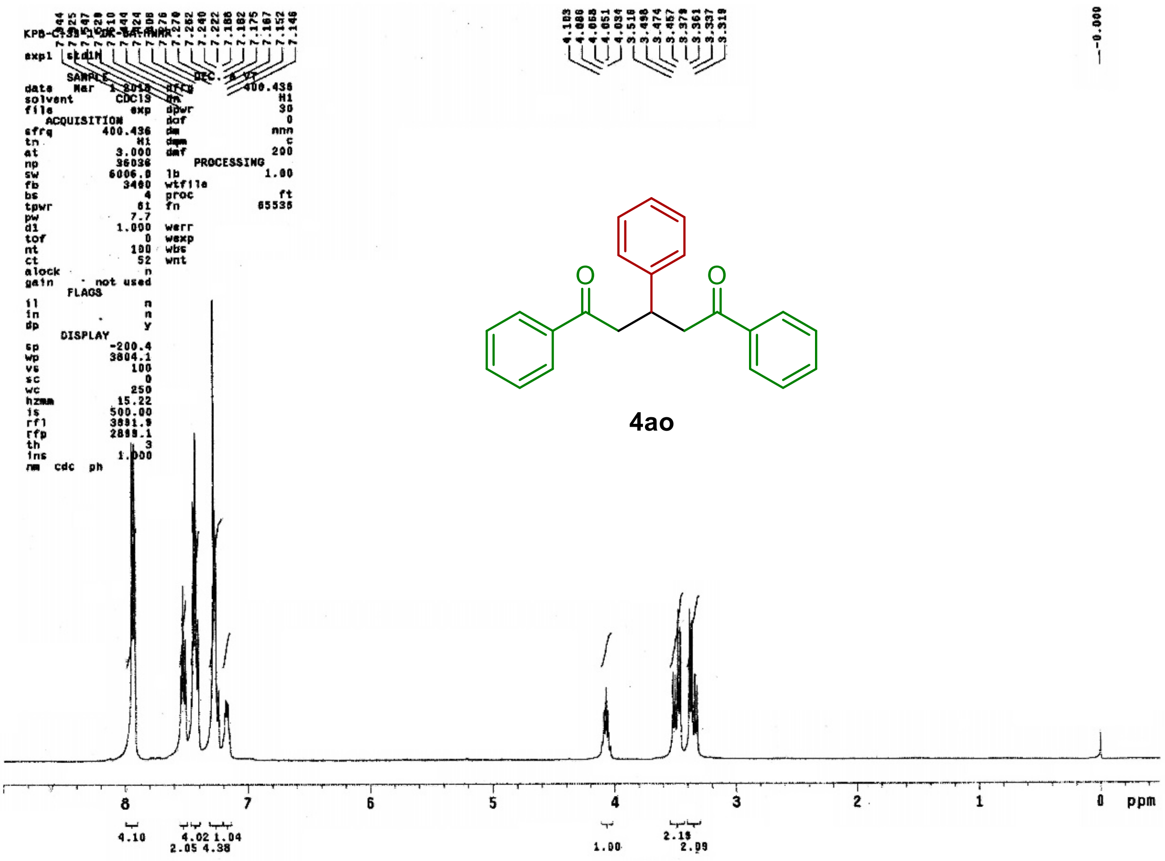

^1H NMR (400 MHz, $CDCl_3$) spectrum of compound **4ao**

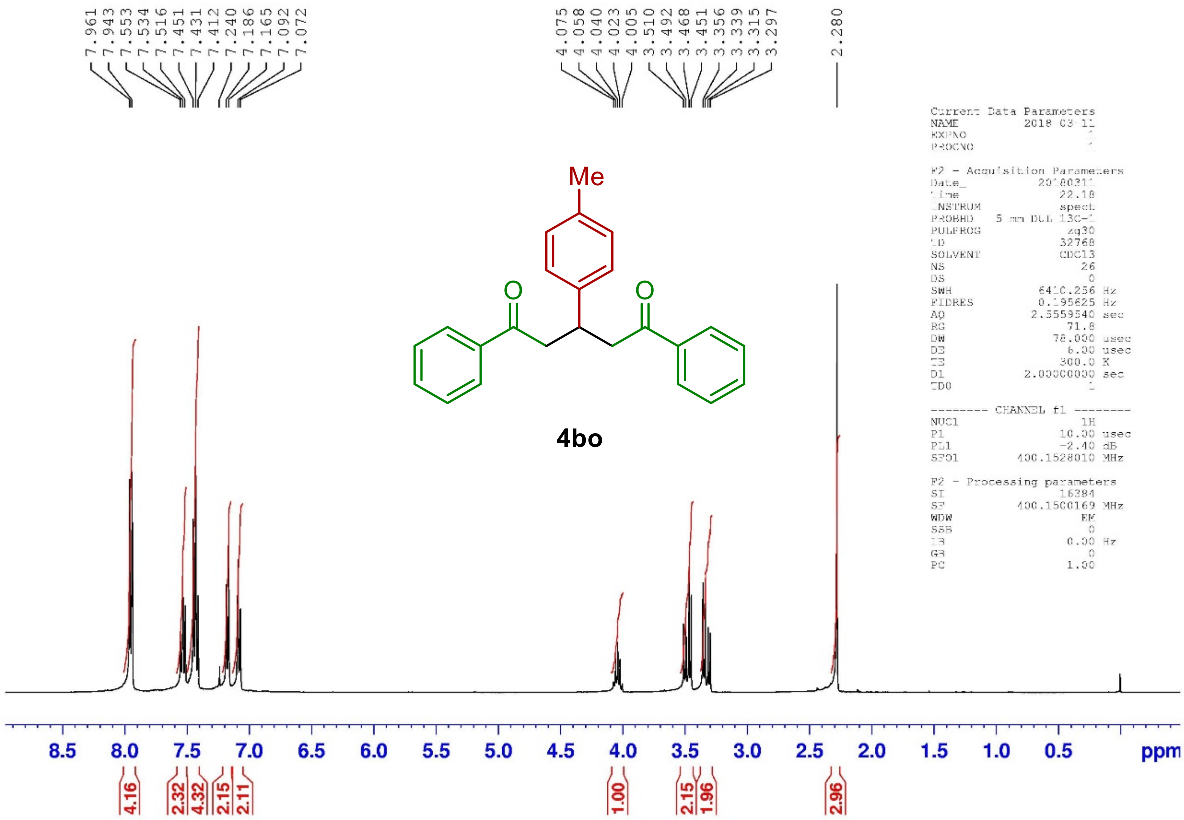

^1H NMR (400 MHz, $CDCl_3$) spectrum of compound **4bo**

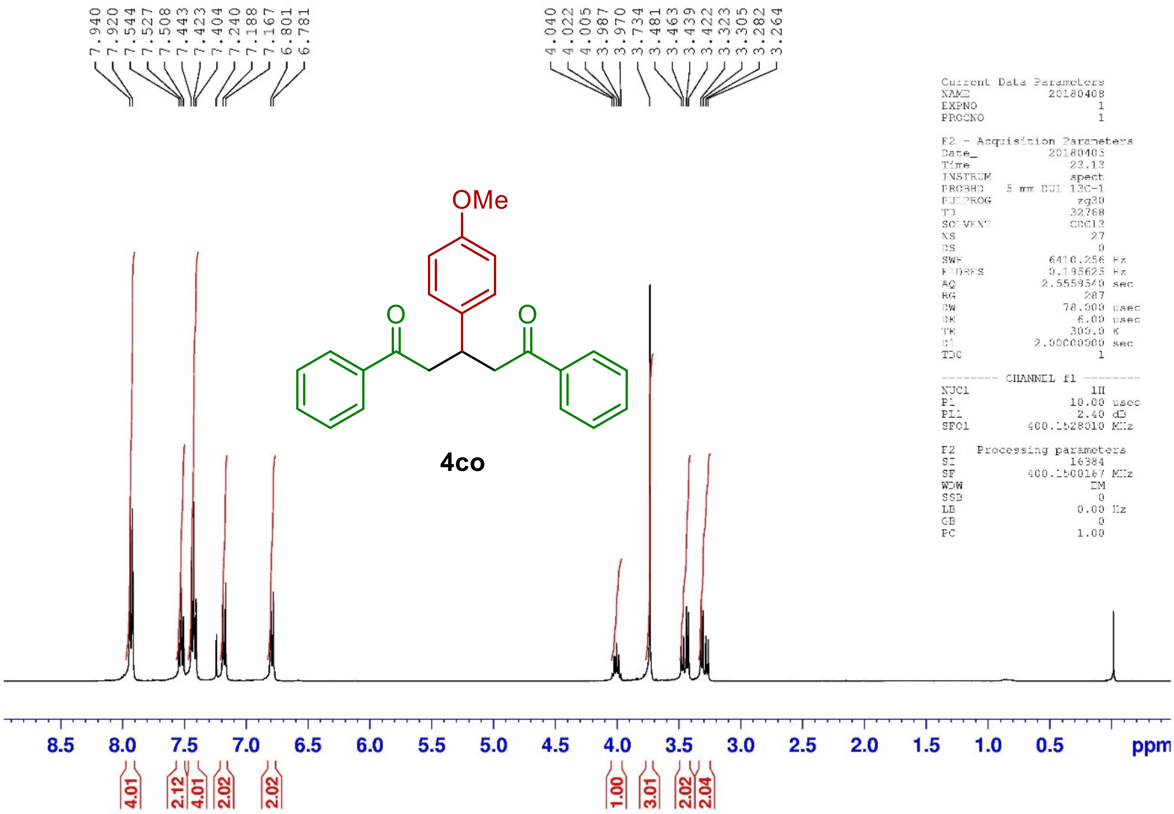

^{1}H NMR (400 MHz, CDCl$_3$) spectrum of compound **4co**

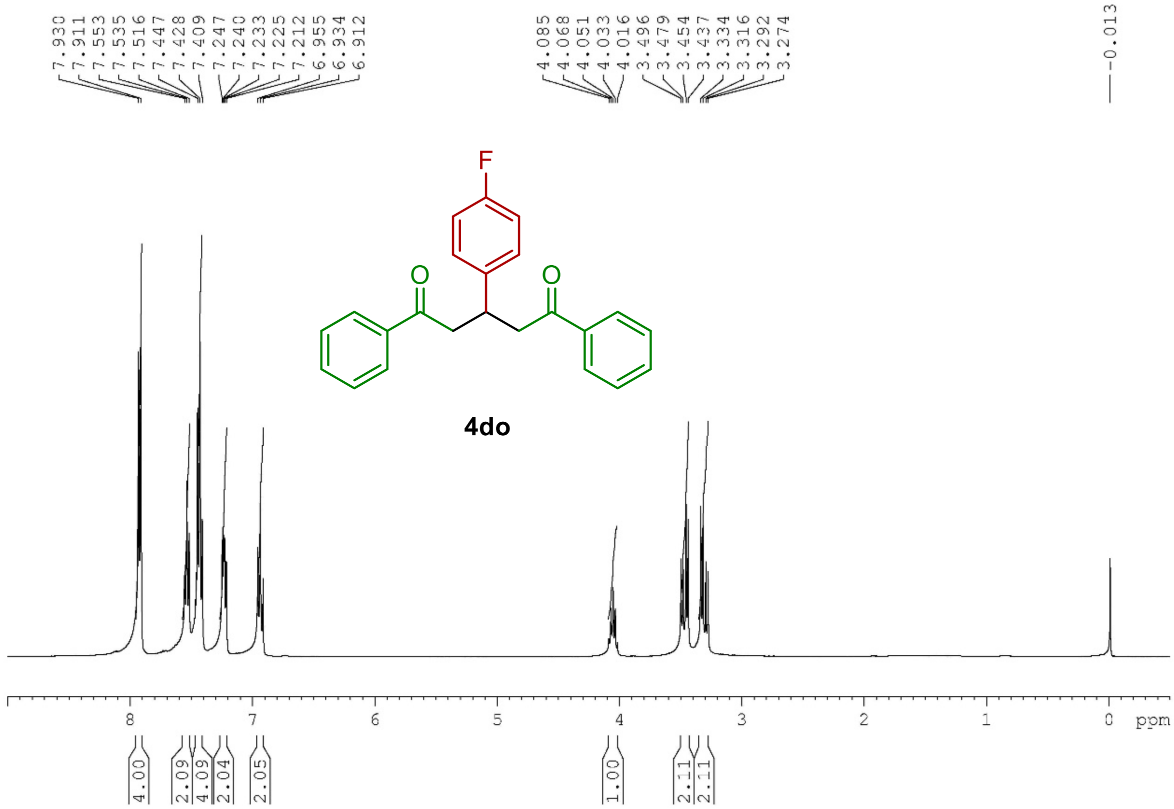

^{1}H NMR (400 MHz, CDCl$_3$) spectrum of compound **4do**

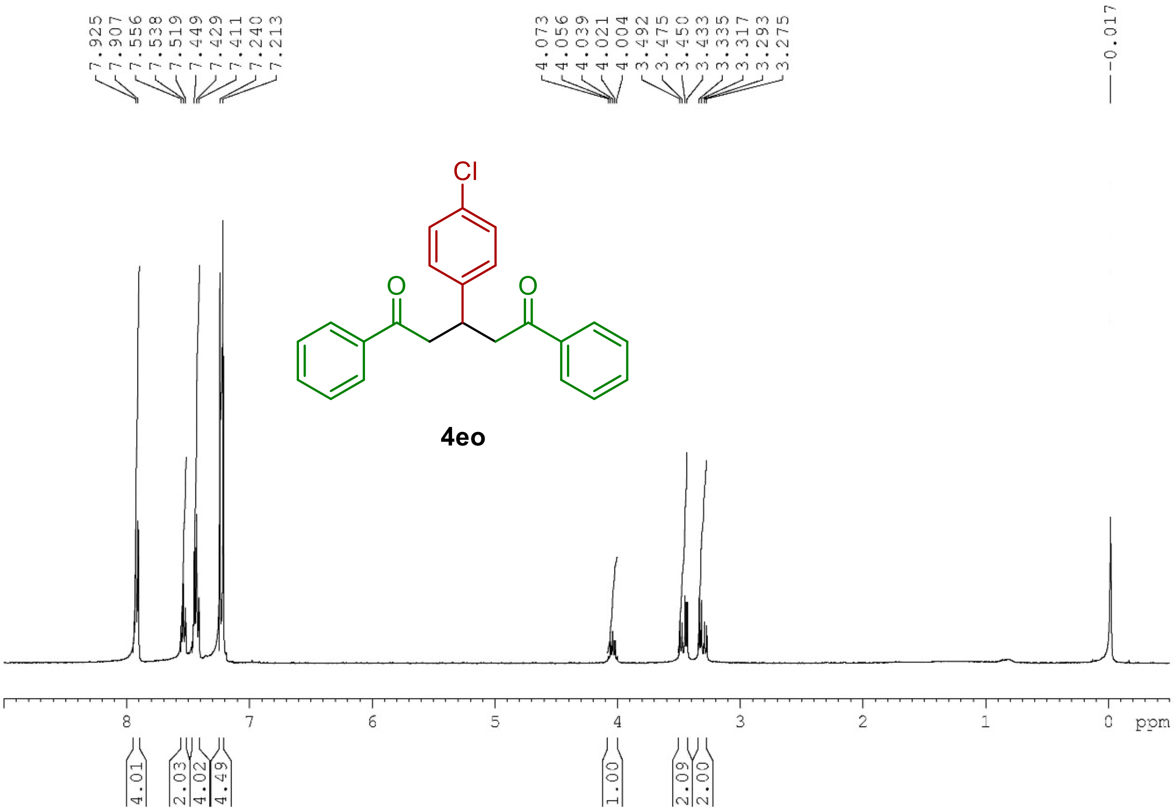

^{1}H NMR (400 MHz, $CDCl_3$) spectrum of compound **4eo**

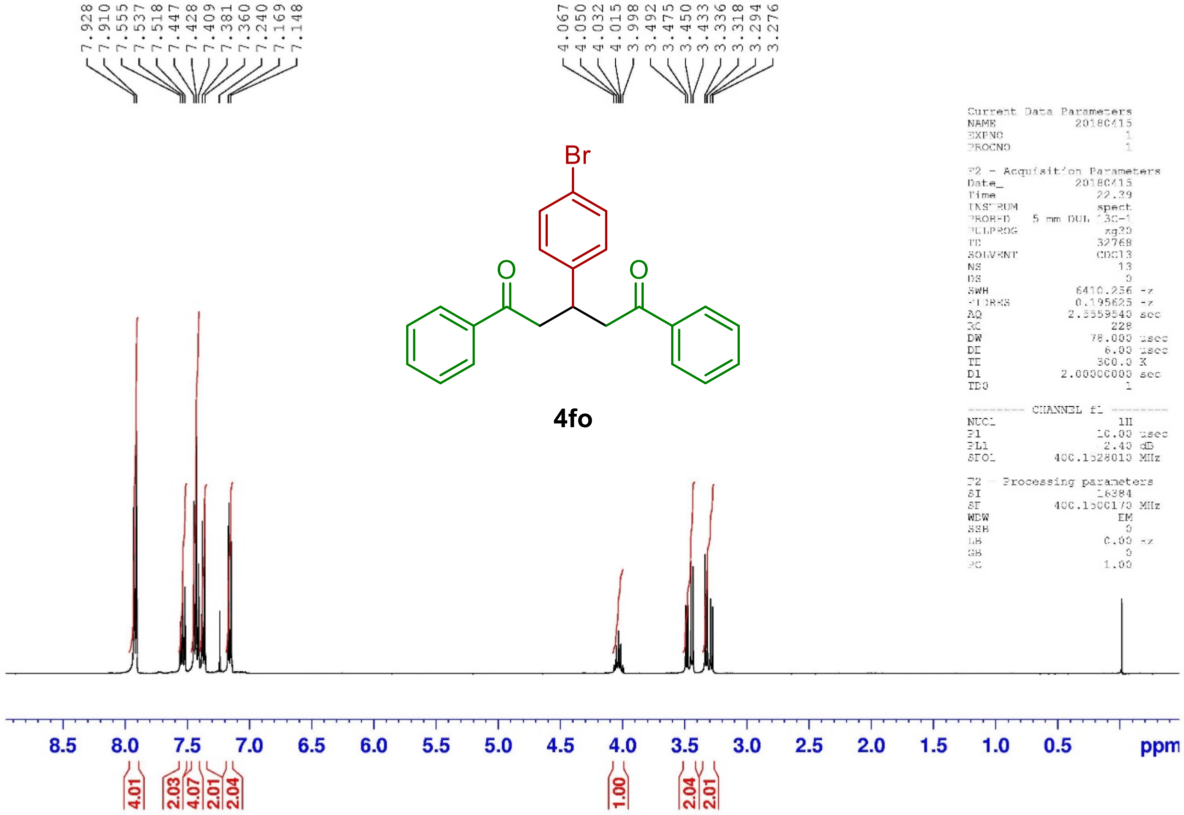

^{1}H NMR (400 MHz, $CDCl_3$) spectrum of compound **4fo**

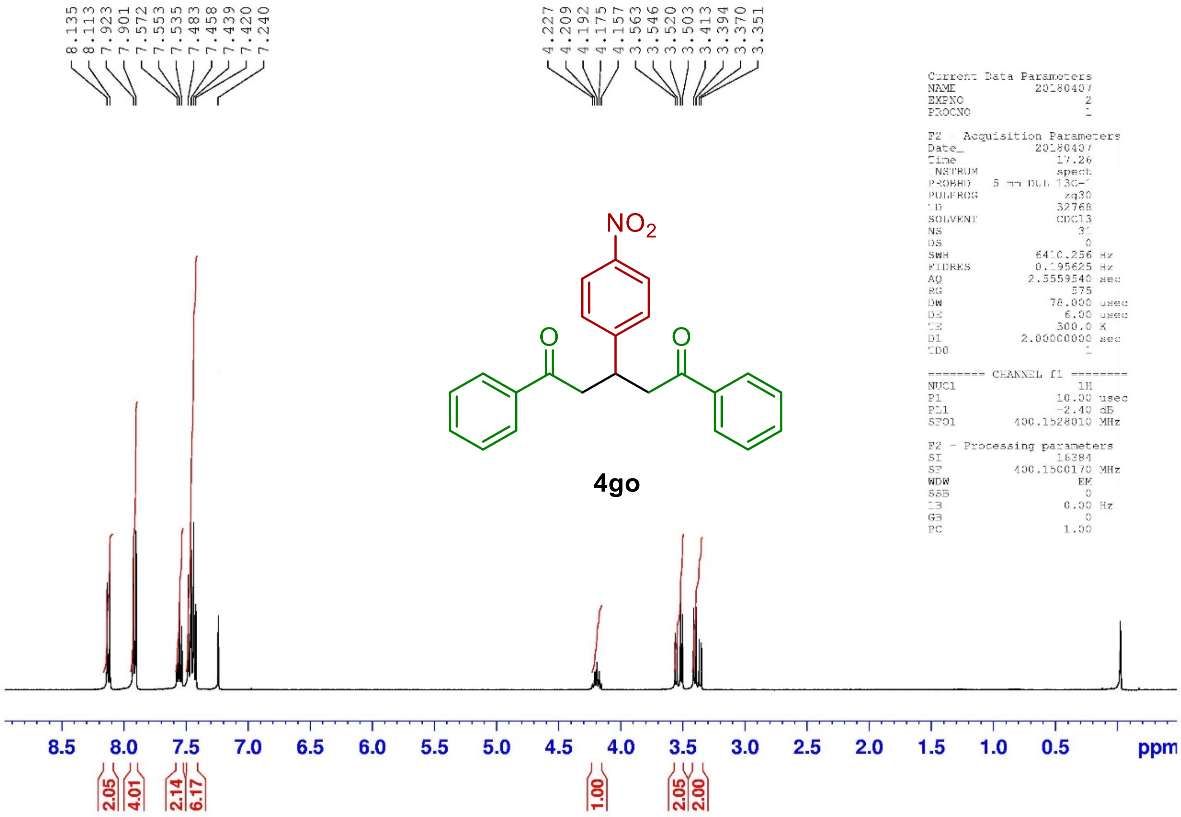

^{1}H NMR (400 MHz, $CDCl_3$) spectrum of compound **4go**

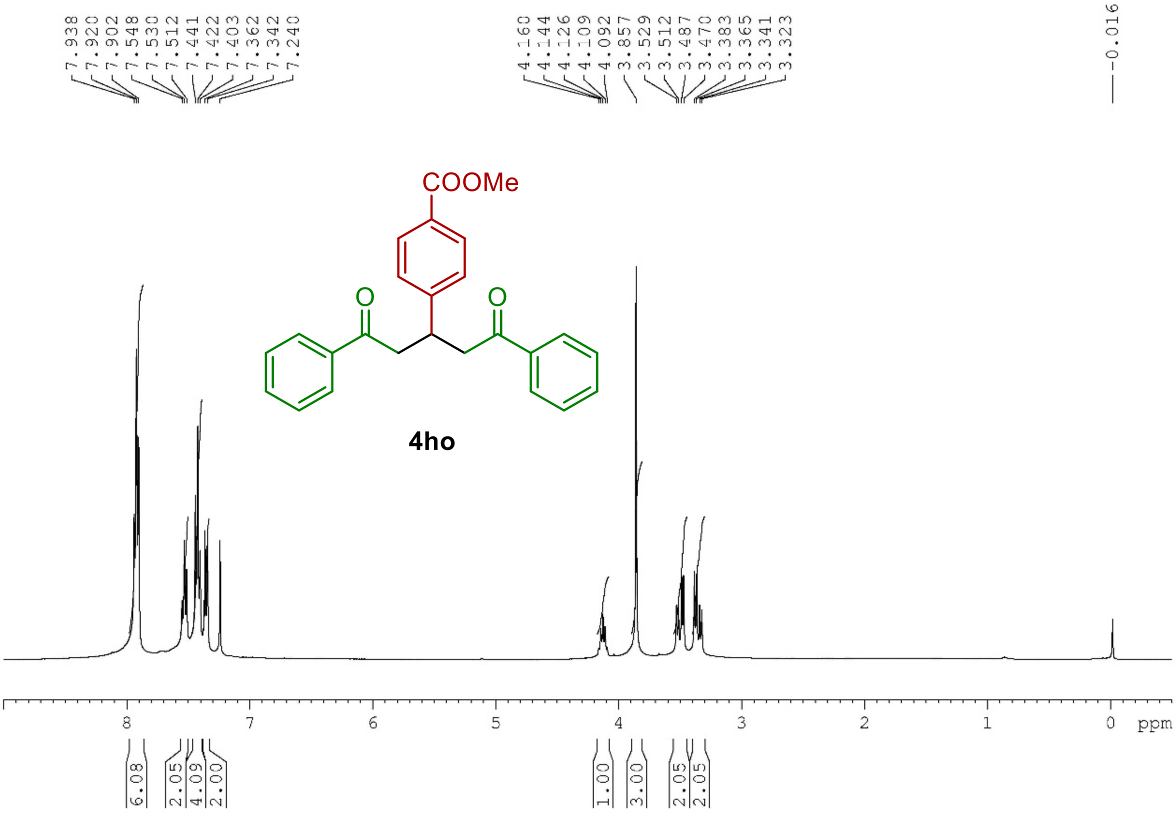

^{1}H NMR (400 MHz, $CDCl_3$) spectrum of compound **4ho**

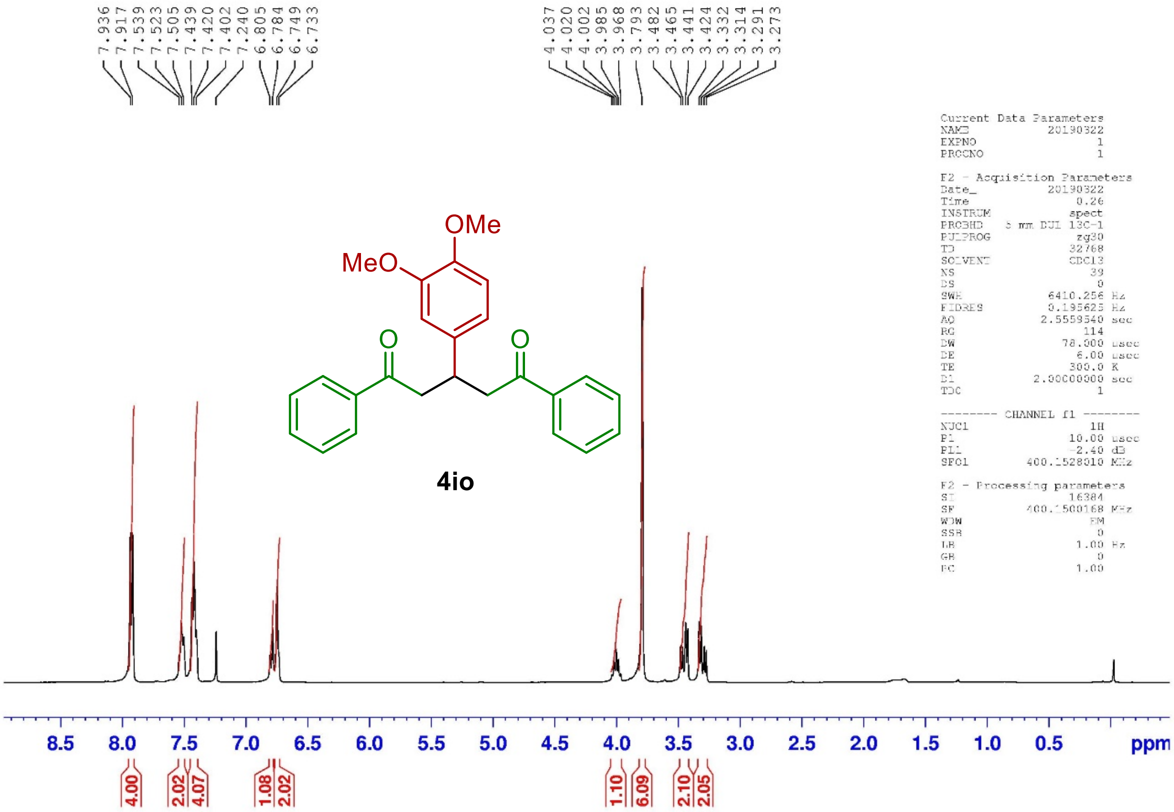

^{1}H NMR (400 MHz, $CDCl_3$) spectrum of compound **4io**

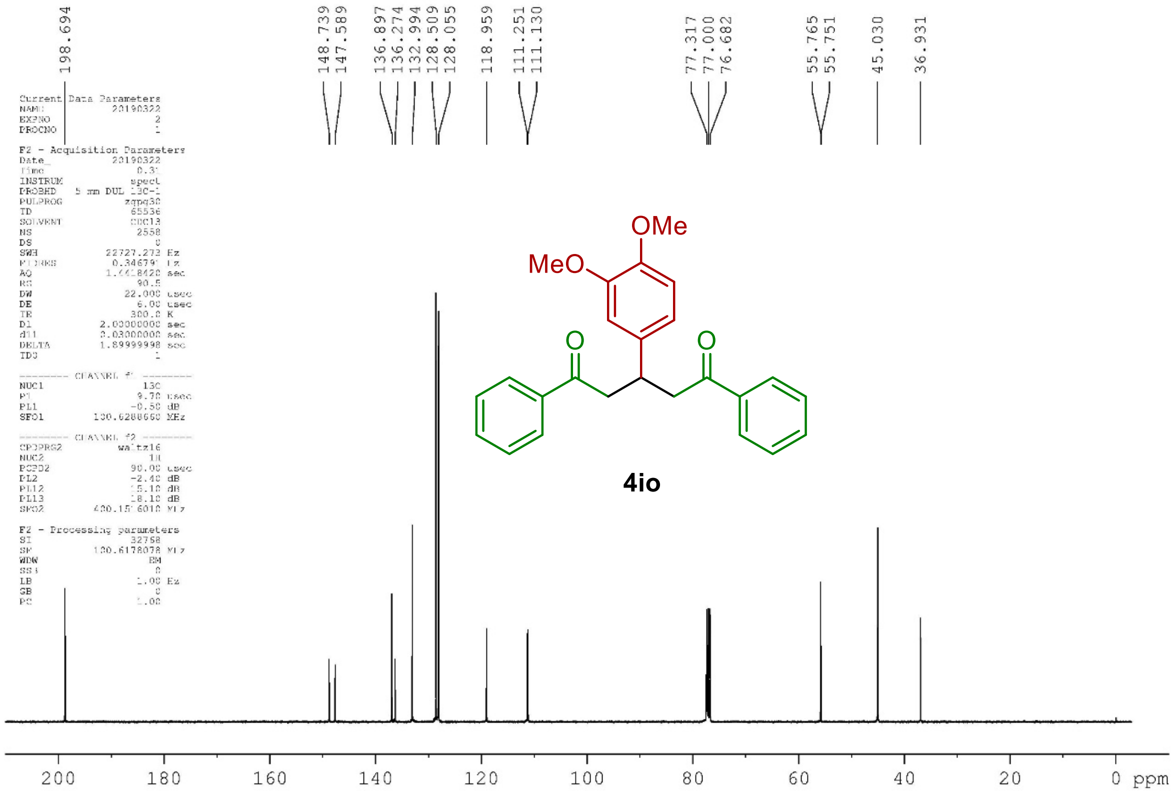

^{13}C{^{1}H} NMR (100 MHz, $CDCl_3$) spectrum of compound **4io**

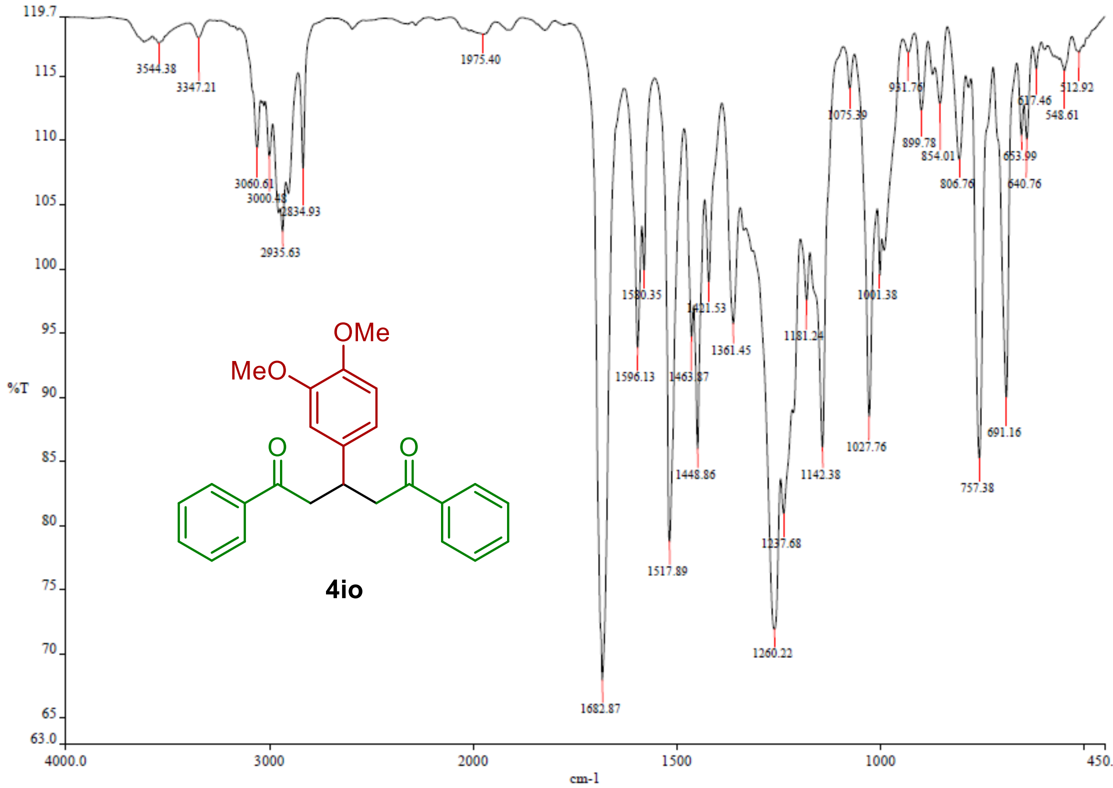

IR spectrum of compound **4io**

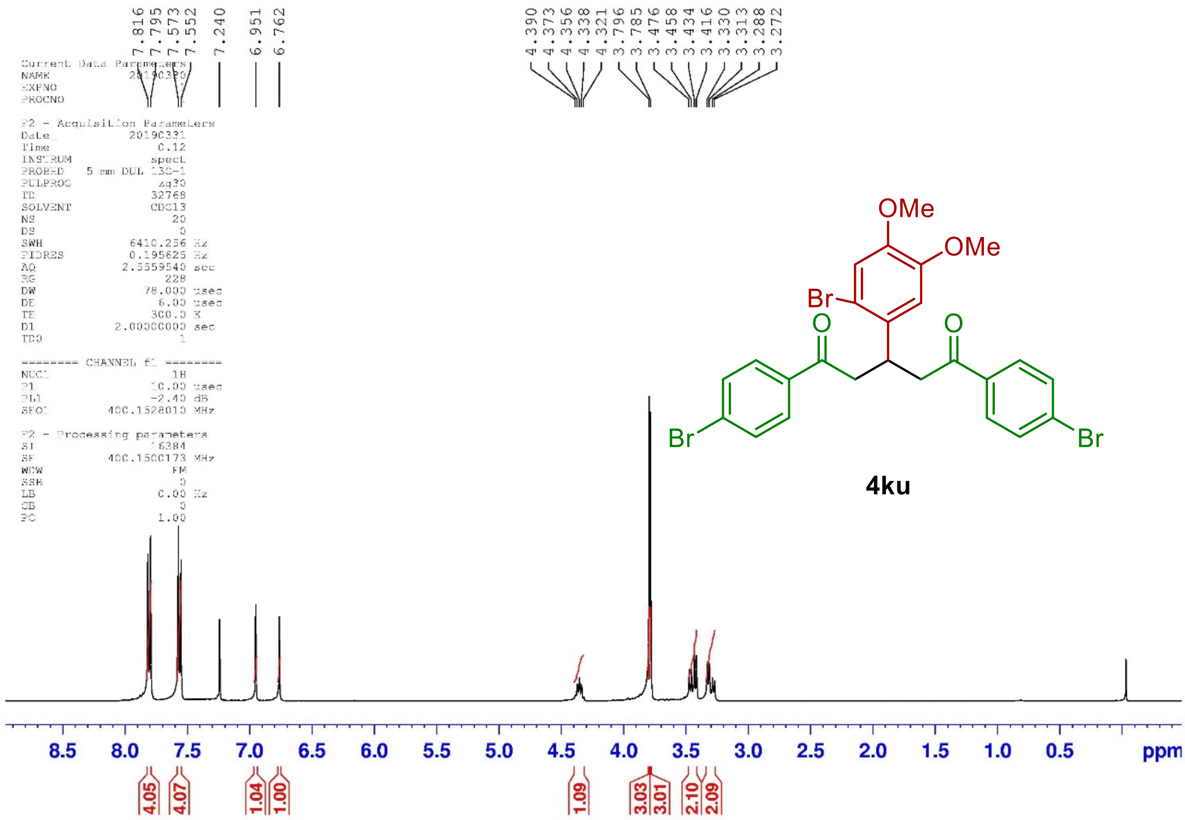

^{1}H NMR (400 MHz, $CDCl_3$) spectrum of compound **4ku**

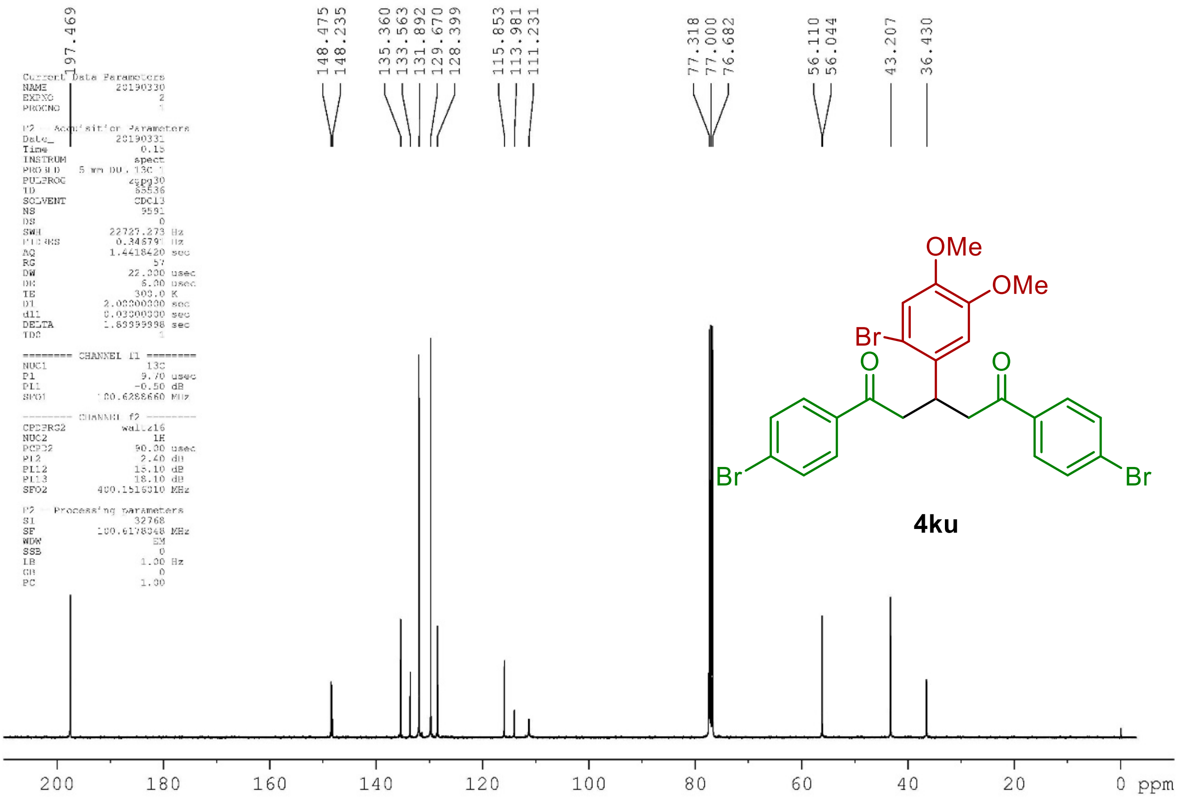

$^{13}C\{^{1}H\}$ NMR (100 MHz, $CDCl_3$) spectrum of compound **4ku**

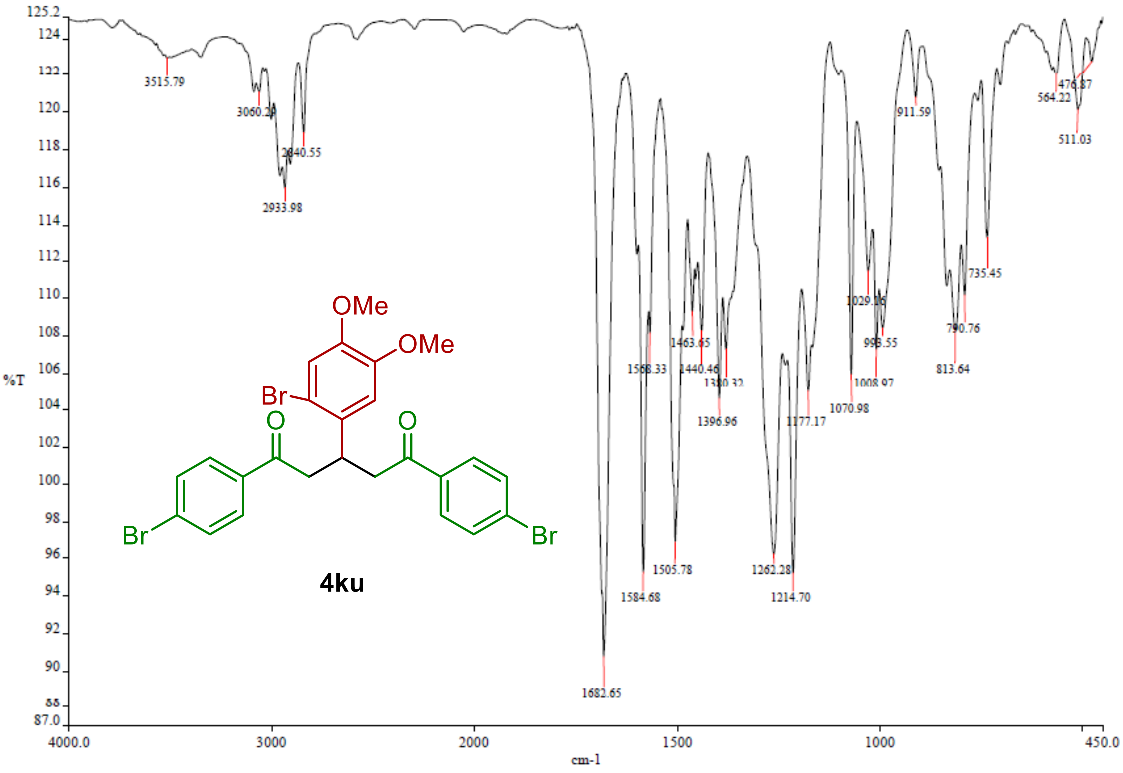

IR spectrum of compound **4ku**

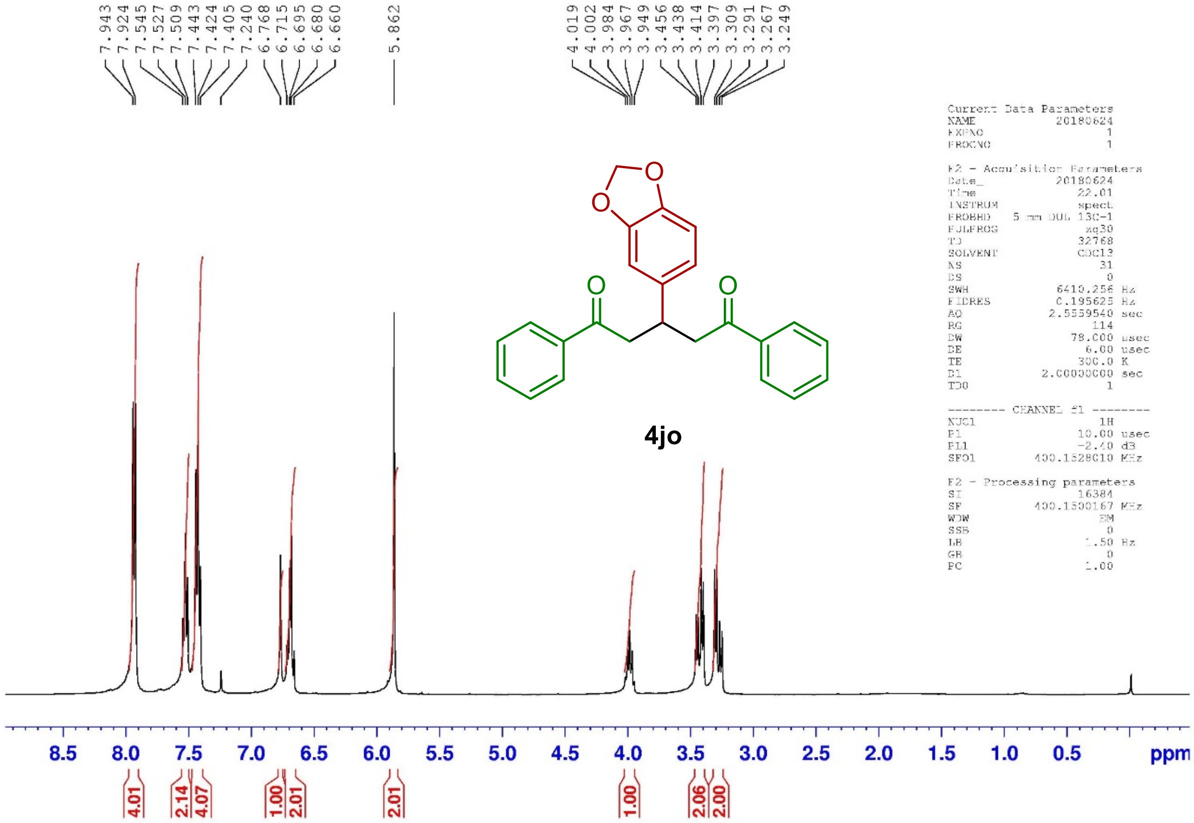

^{1}H NMR (400 MHz, $CDCl_3$) spectrum of compound **4jo**

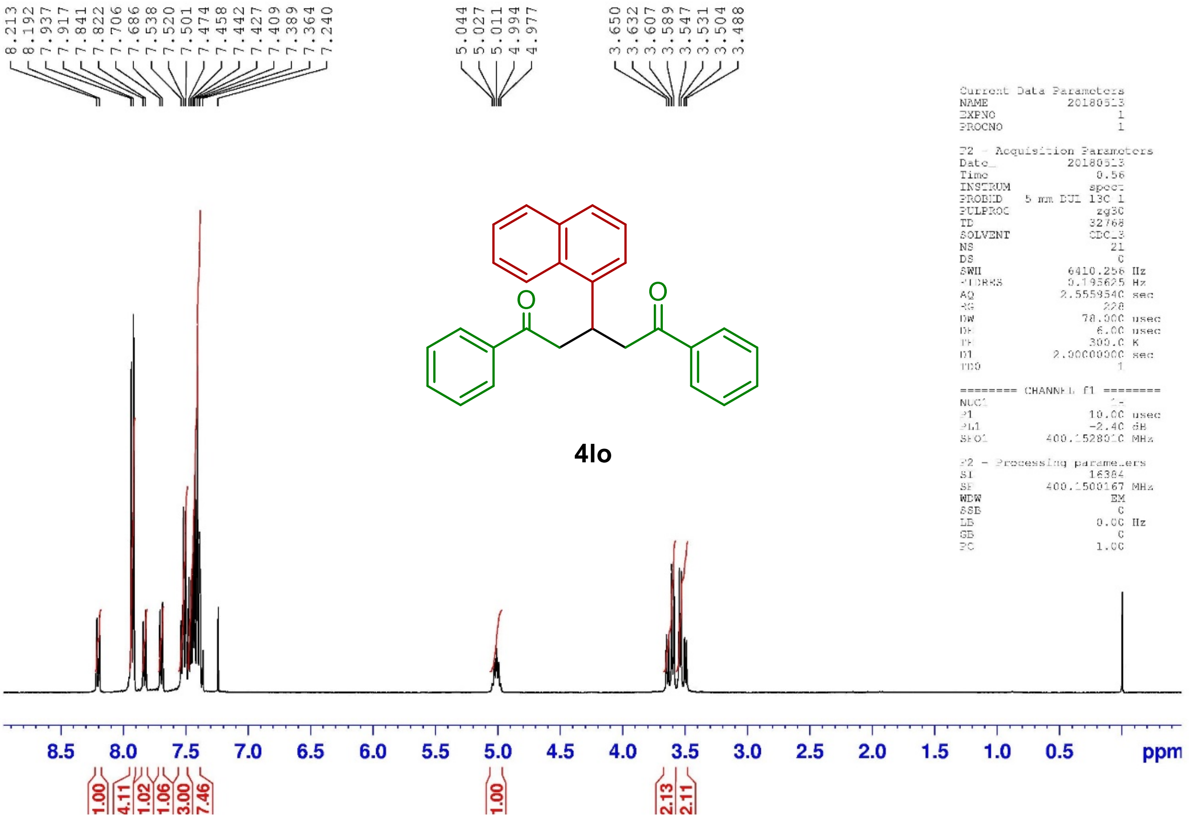

^{1}H NMR (400 MHz, $CDCl_3$) spectrum of compound **4lo**

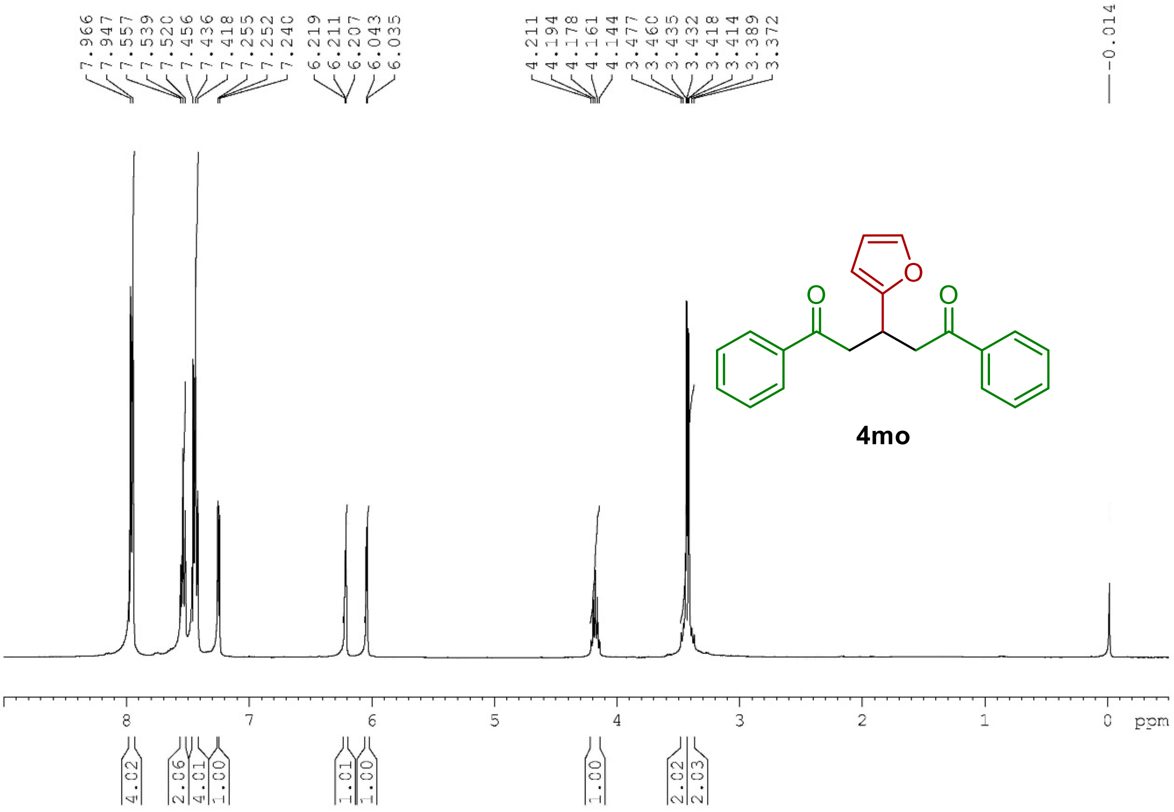

^{1}H NMR (400 MHz, $CDCl_3$) spectrum of compound **4mo**

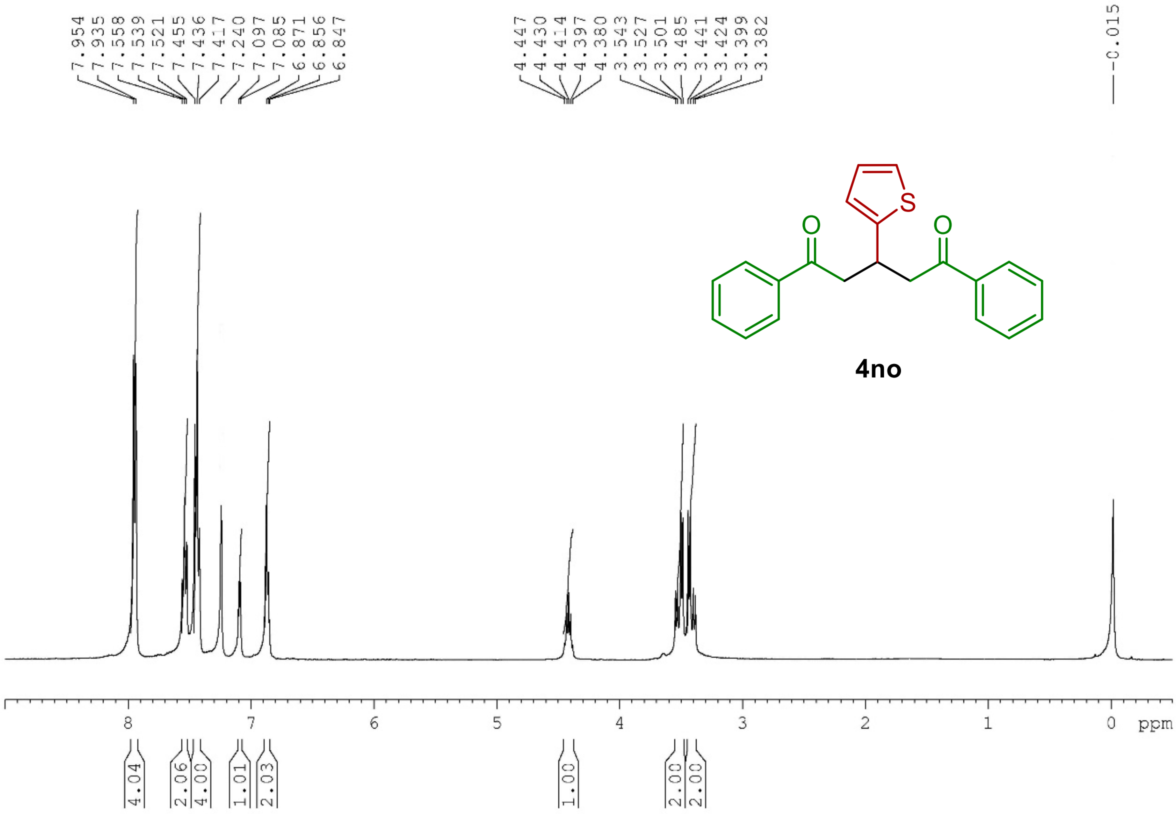

^{1}H NMR (400 MHz, $CDCl_3$) spectrum of compound **4no**

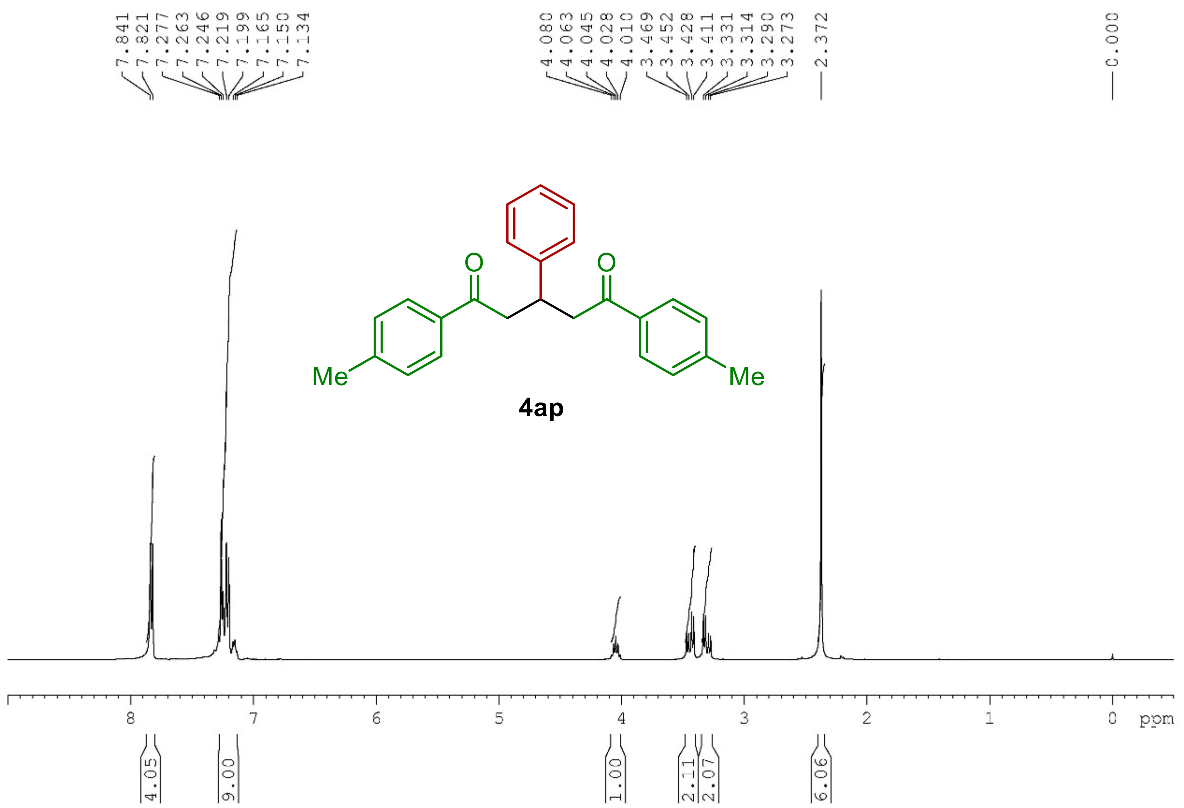

^{1}H NMR (400 MHz, $CDCl_3$) spectrum of compound **4ap**

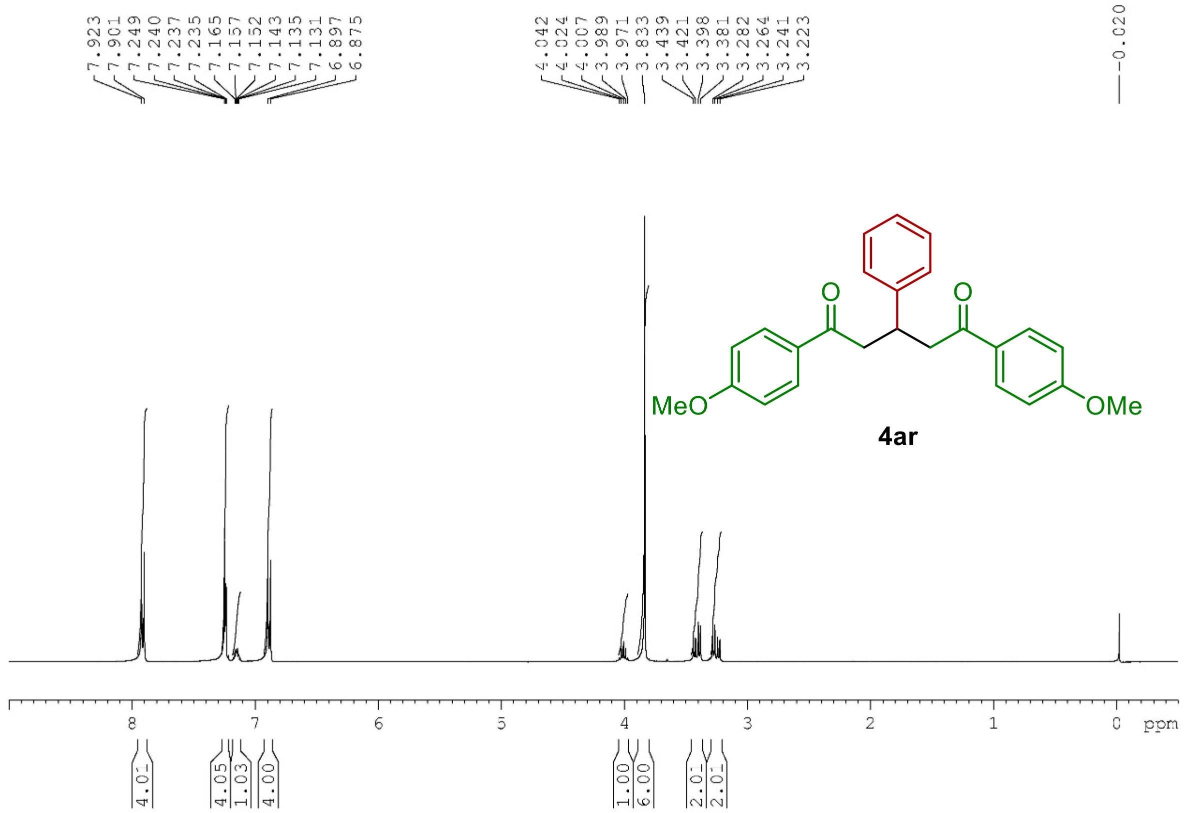

^{1}H NMR (400 MHz, $CDCl_3$) spectrum of compound **4ar**

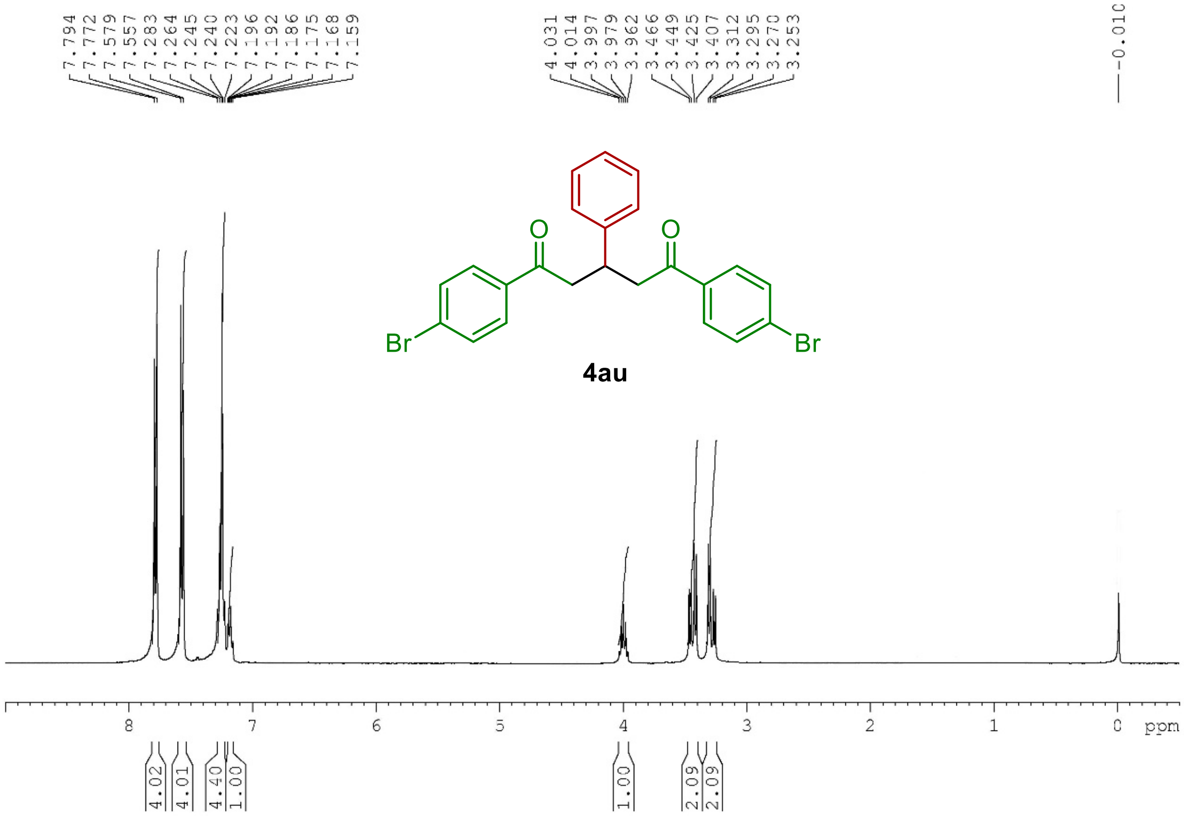

^{1}H NMR (400 MHz, $CDCl_3$) spectrum of compound **4au**

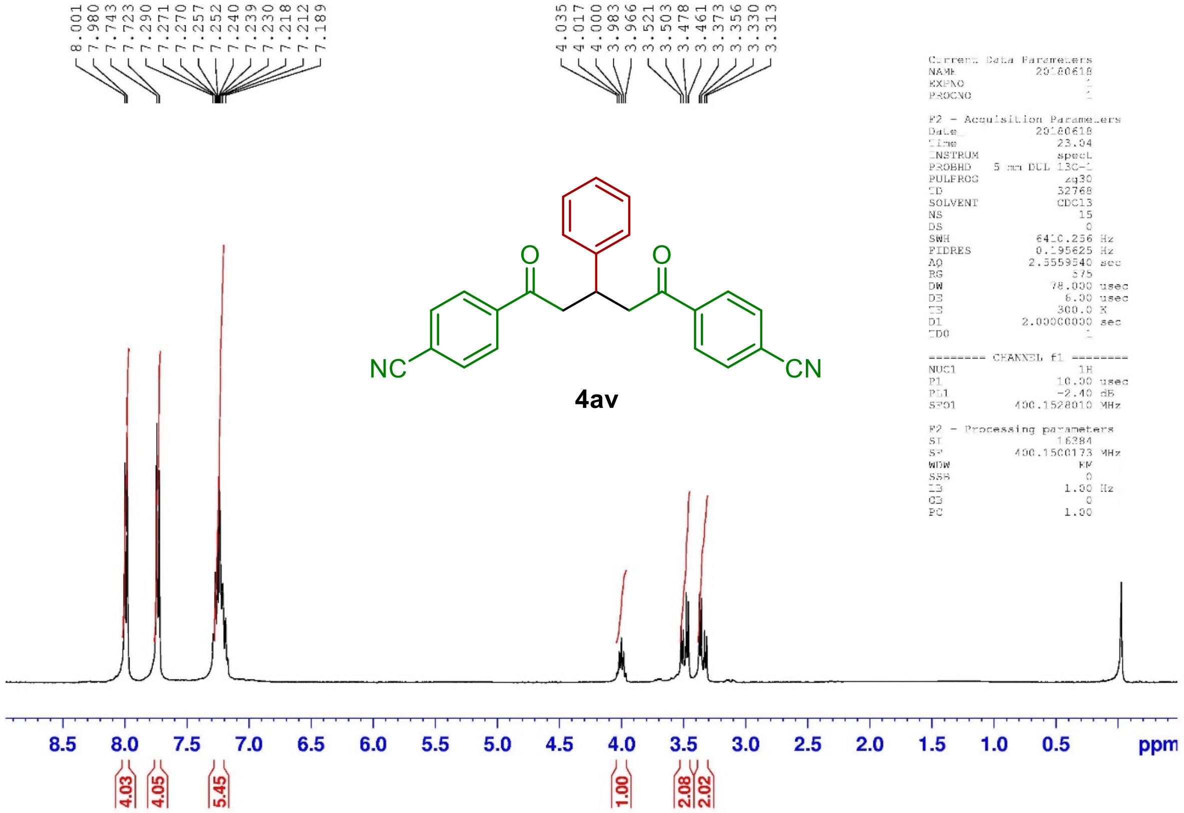

^{1}H NMR (400 MHz, $CDCl_3$) spectrum of compound **4av**

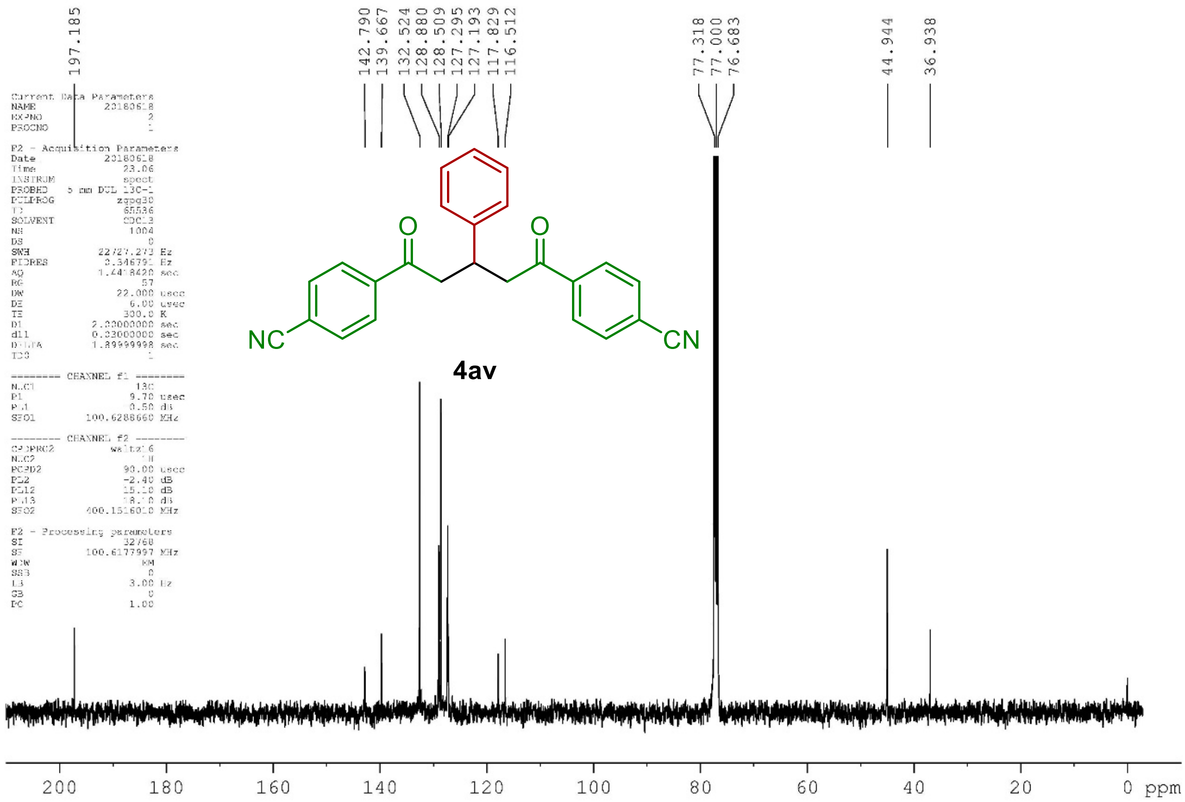

$^{13}C\{^1H\}$ NMR (100 MHz, $CDCl_3$) spectrum of compound **4av**

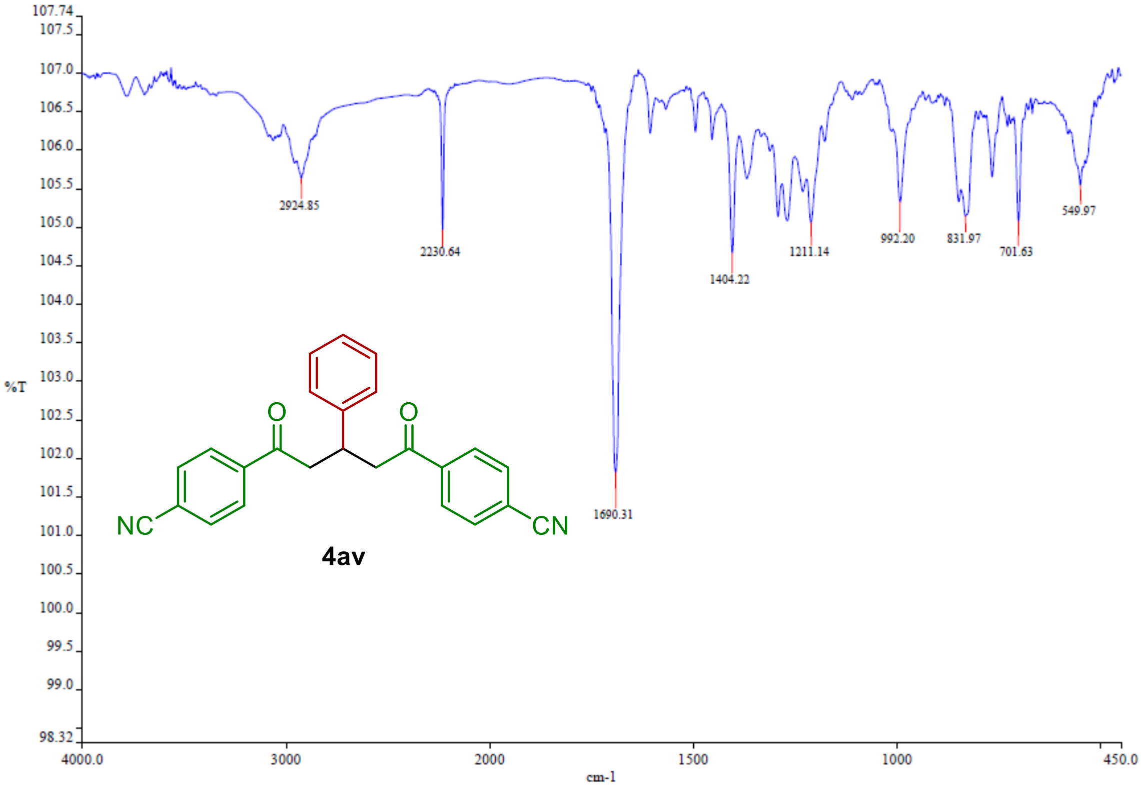

IR spectrum of compound **4av**

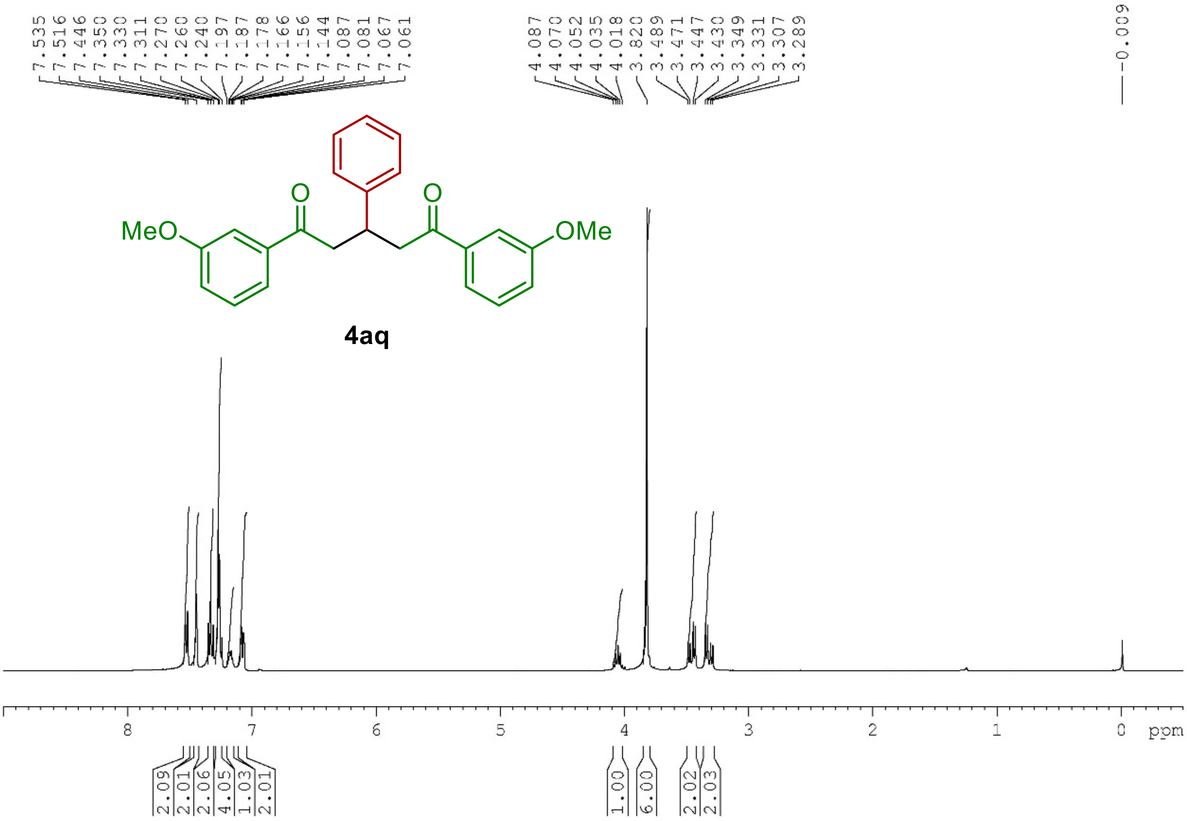

^{1}H NMR (400 MHz, CDCl$_3$) spectrum of compound **4aq**

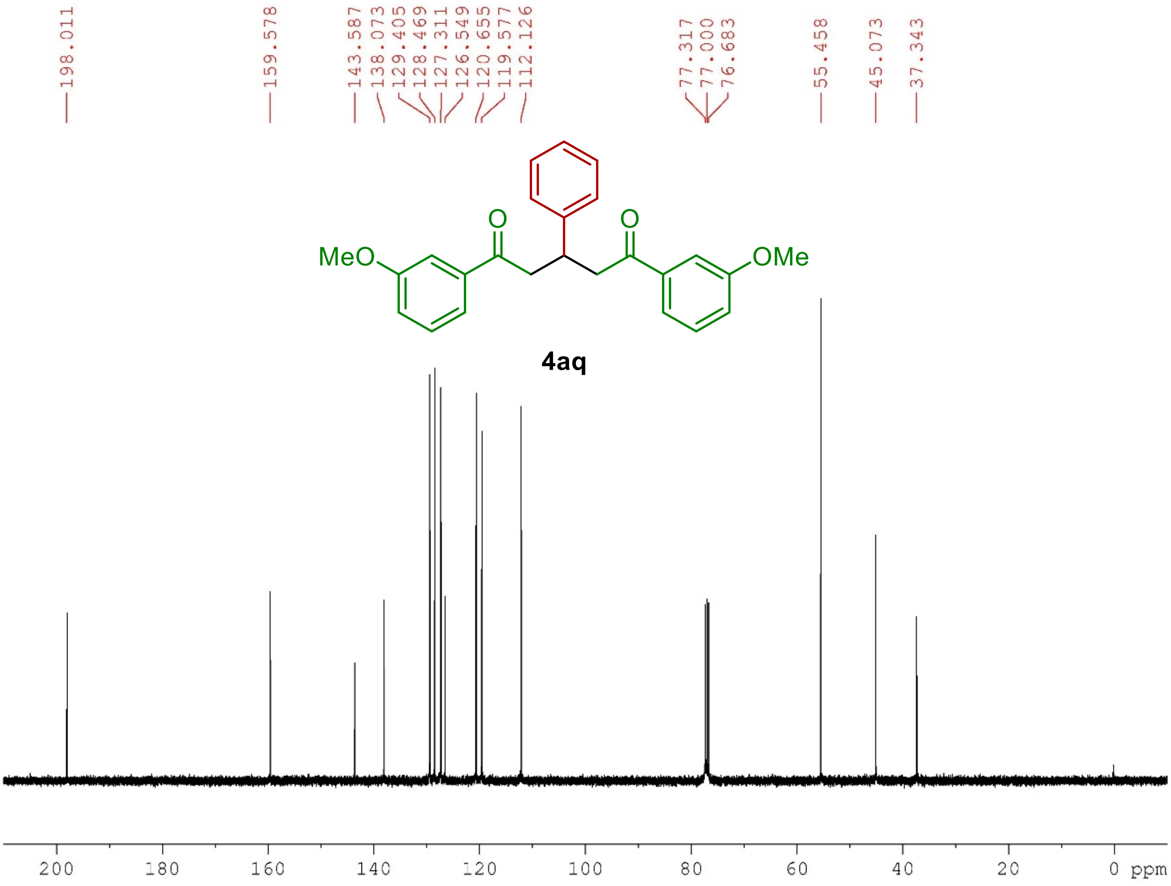

^{13}C{^{1}H} NMR (100 MHz, CDCl$_3$) spectrum of compound **4aq**

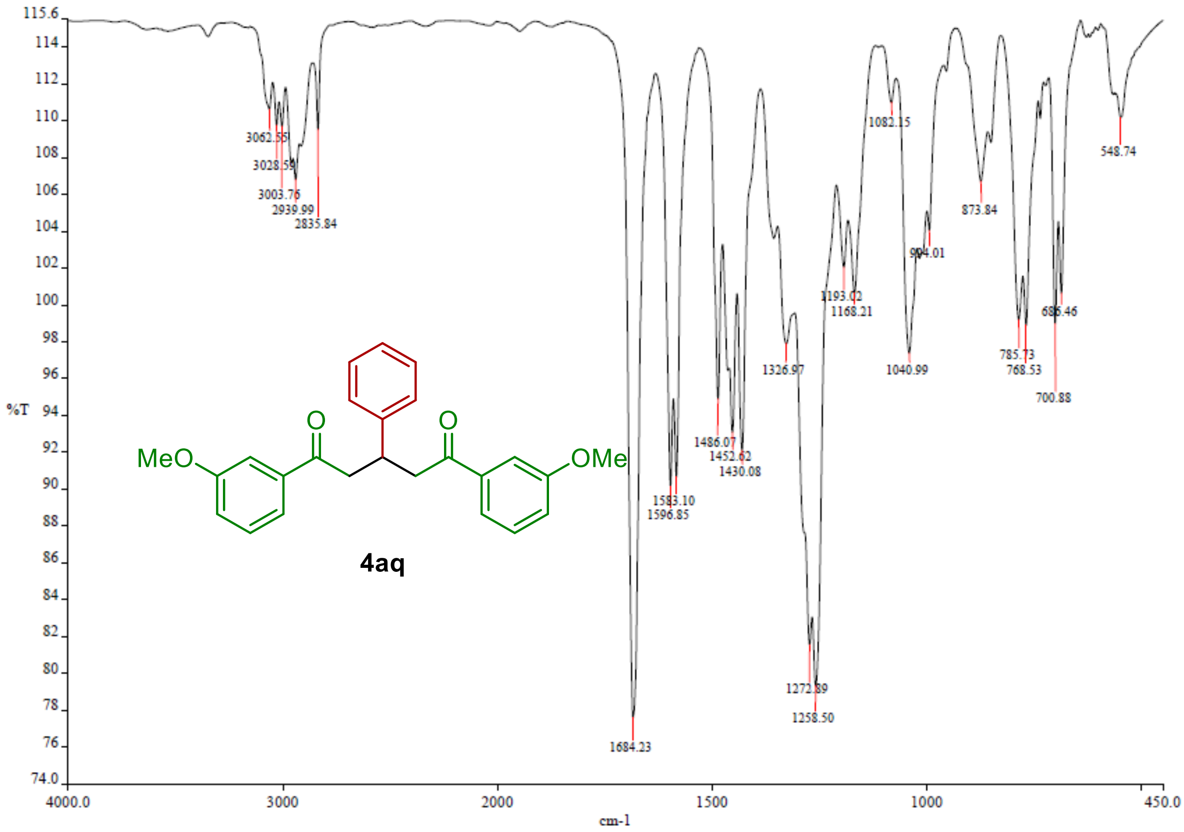

IR spectrum of compound **4aq**

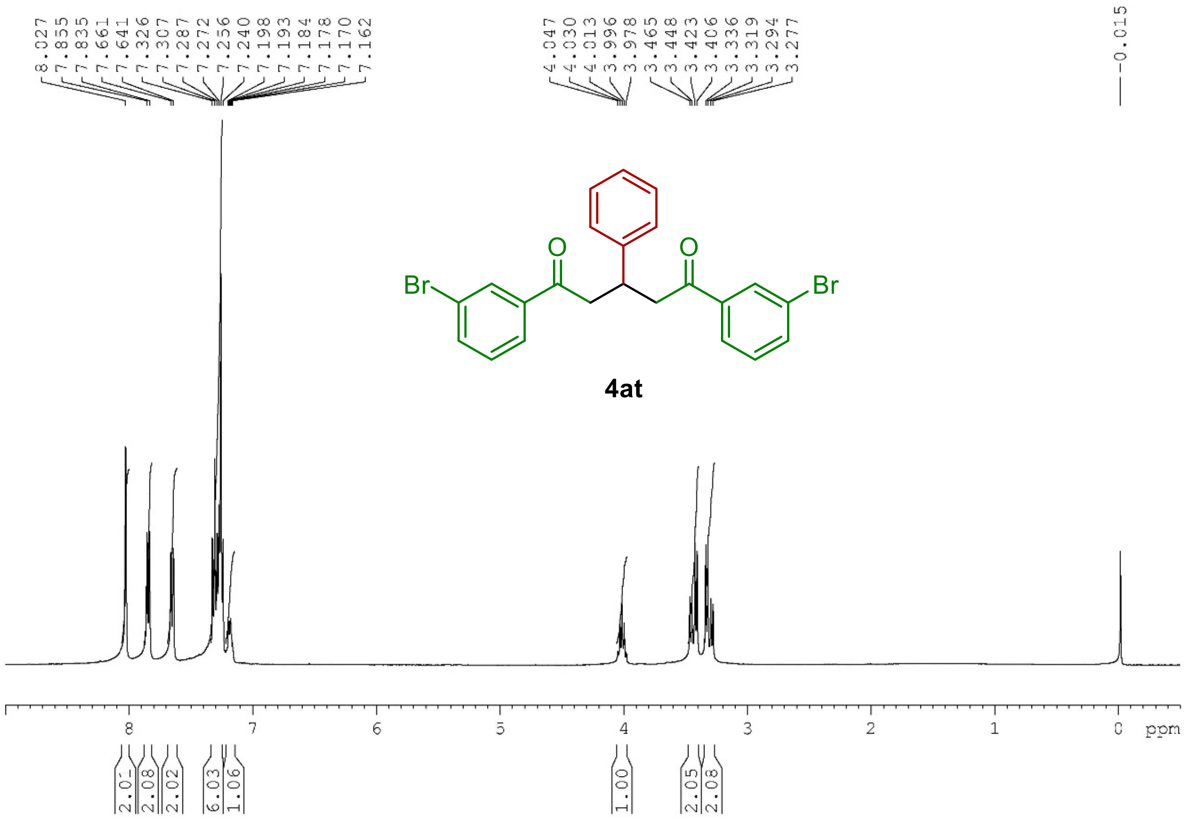

^{1}H NMR (400 MHz, $CDCl_3$) spectrum of compound **4at**

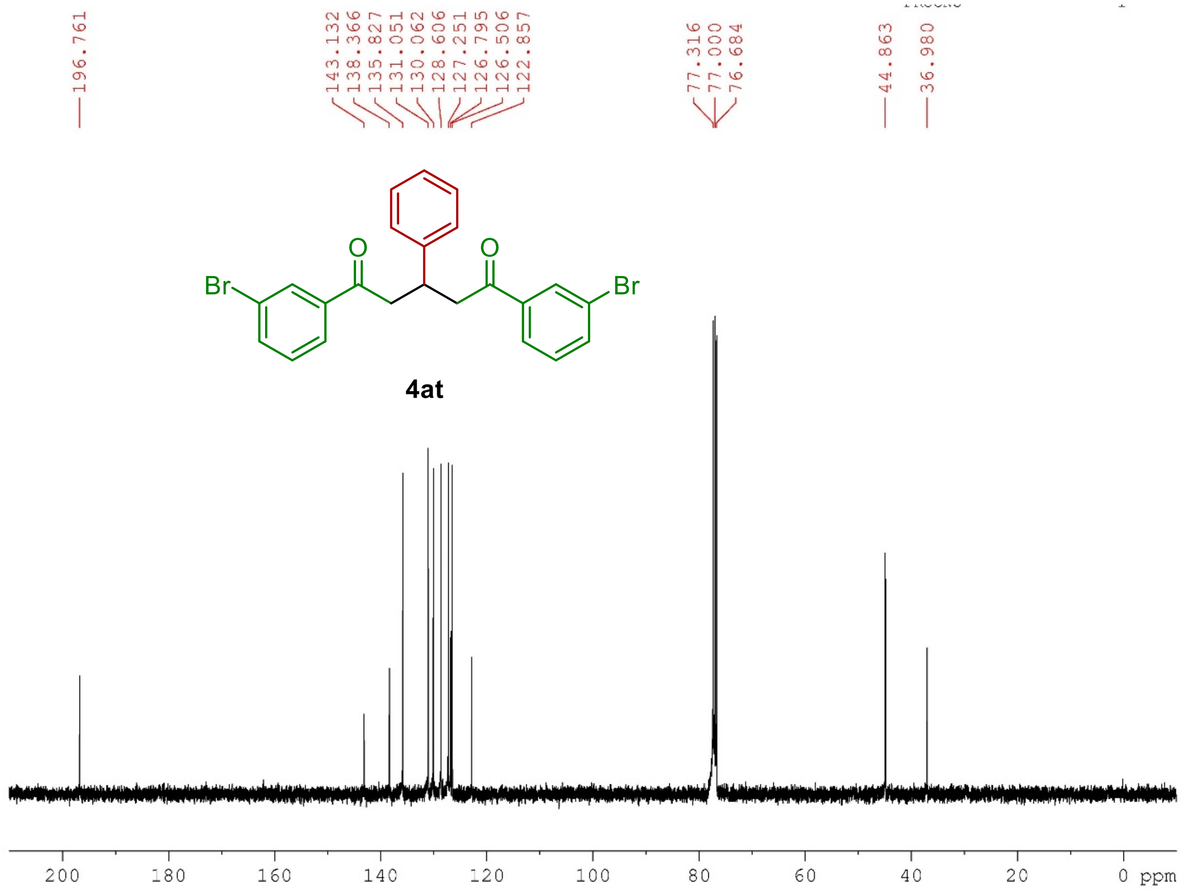

$^{13}C\{^{1}H\}$ NMR (100 MHz, $CDCl_3$) spectrum of compound **4at**

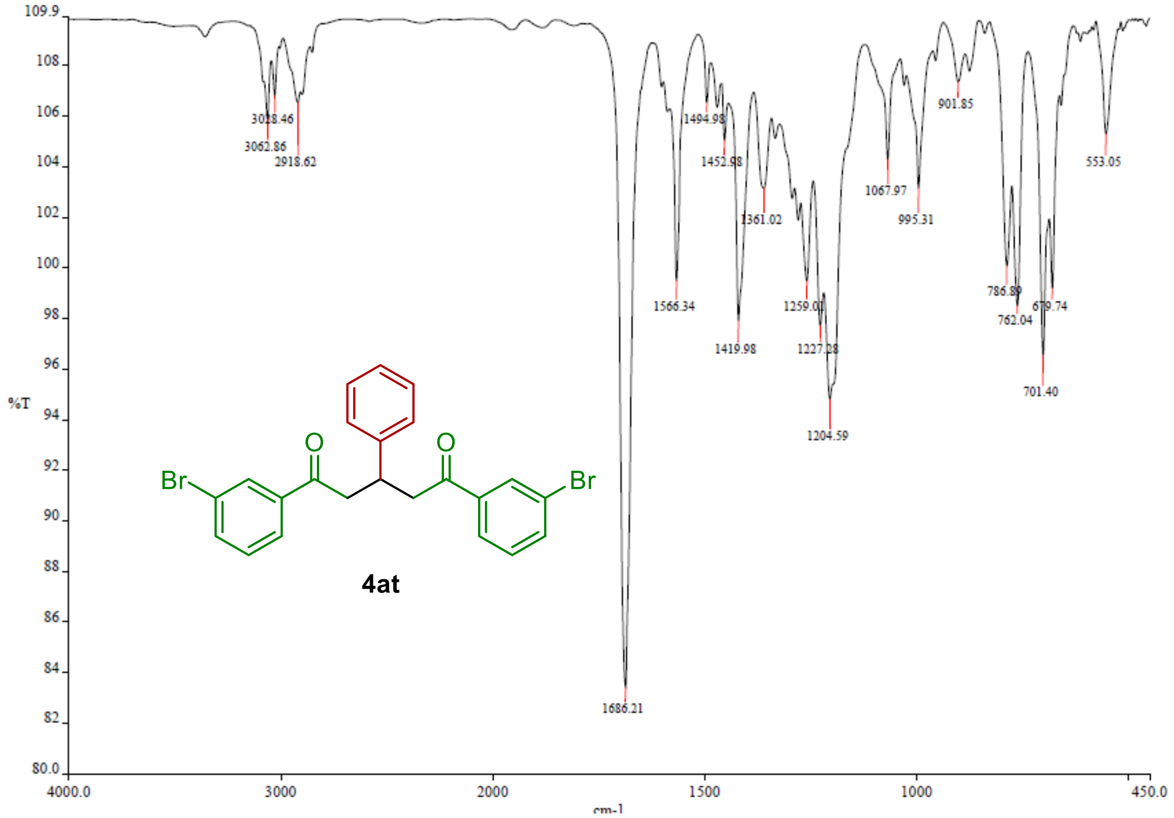

IR spectrum of compound **4at**

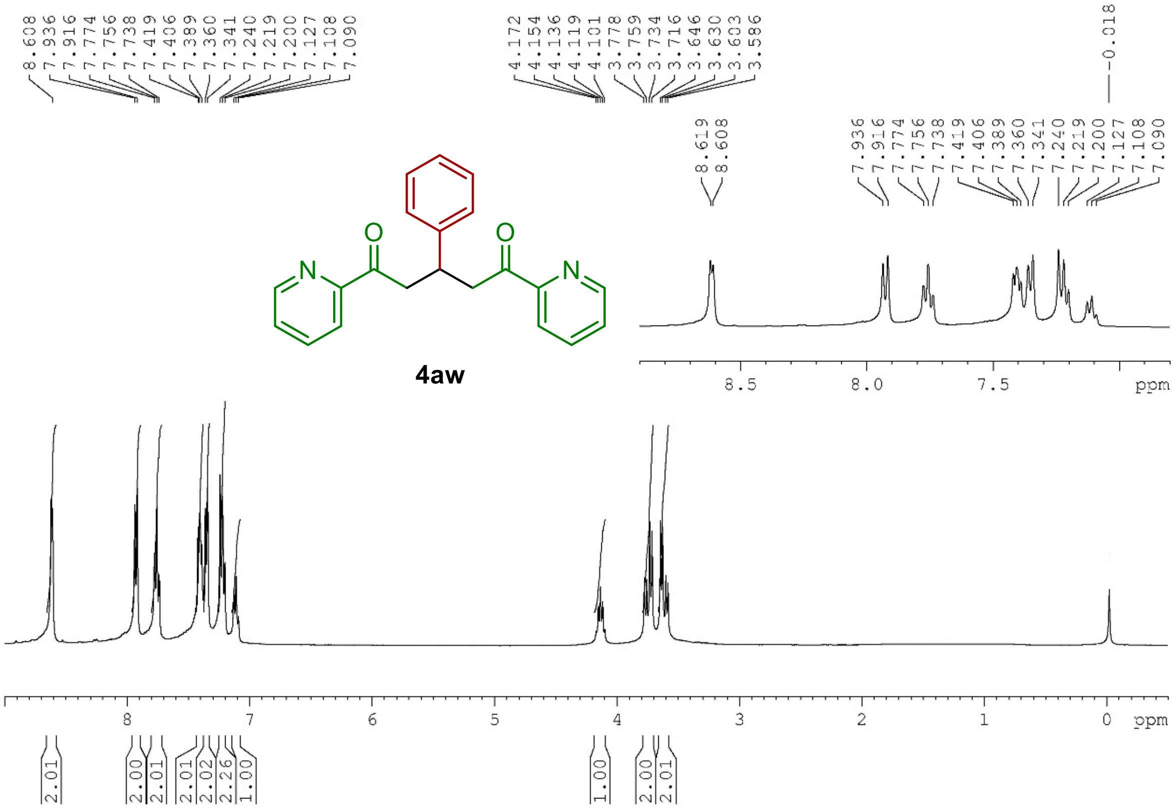

^1H NMR (400 MHz, $CDCl_3$) spectrum of compound **4aw**
